# Electron‐Rich EDOT Linkers in Tetracationic bis‐Triarylborane Chromophores: Influence on Water Stability, Biomacromolecule Sensing, and Photoinduced Cytotoxicity

**DOI:** 10.1002/chem.202201130

**Published:** 2022-07-04

**Authors:** Matthias Ferger, Chantal Roger, Eva Köster, Florian Rauch, Sabine Lorenzen, Ivo Krummenacher, Alexandra Friedrich, Marta Košćak, Davor Nestić, Holger Braunschweig, Christoph Lambert, Ivo Piantanida, Todd B. Marder

**Affiliations:** ^1^ Institut für Anorganische Chemie and Institute for Sustainable Chemistry & Catalysis with Boron Julius-Maximilians-Universität Würzburg Am Hubland 97074 Würzburg Germany; ^2^ Institut für Organische Chemie Julius-Maximilians-Universität Würzburg Am Hubland 97074 Würzburg Germany; ^3^ Division of Organic Chemistry and Biochemistry Ruđer Bošković Institute Bijenicka c. 54 10000 Zagreb Croatia; ^4^ Division of Molecular Biology Ruđer Bošković Institute Bijenicka c. 54 10000 Zagreb Croatia

**Keywords:** boranes, DNA/RNA sensors, fluorescent probes, singlet oxygen, theranostics

## Abstract

Three novel tetracationic bis‐triarylboranes with 3,4‐ethylenedioxythiophene (EDOT) linkers, and their neutral precursors, showed significant red‐shifted absorption and emission compared to their thiophene‐containing analogues, with one of the EDOT‐derivatives emitting in the NIR region. Only the EDOT‐linked trixylylborane tetracation was stable in aqueous solution, indicating that direct attachment of a thiophene or even 3‐methylthiophene to the boron atom is insufficient to provide hydrolytic stability in aqueous solution. Further comparative analysis of the EDOT‐linked trixylylborane tetracation and its bis‐thiophene analogue revealed efficient photo‐induced singlet oxygen production, with the consequent biological implications. Thus, both analogues bind strongly to ds‐DNA and BSA, very efficiently enter living human cells, accumulate in several different cytoplasmic organelles with no toxic effect but, under intense visible light irradiation, they exhibit almost instantaneous and very strong cytotoxic effects, presumably attributed to singlet oxygen production. Thus, both compounds are intriguing theranostic agents, whose intracellular and probably intra‐tissue location can be monitored by strong fluorescence, allowing switching on of the strong bioactivity by well‐focused visible light.

## Graphical abstract

**Tetracationic bis‐triarylboranes** with EDOT linkers, show red‐shifted absorption and emission compared to their thiophene‐linked analogues, with one derivative emitting in the NIR. A water‐stable EDOT‐linked tetracation and its bis‐thiophene analogue efficiently photo‐induce ^1^O_2_ production. Both bind to ds‐DNA and BSA, enter living human cells, accumulate in several different cytoplasmic organelles with no toxic effect but, under visible light irradiation, exhibit rapid, strong cytotoxic effects.

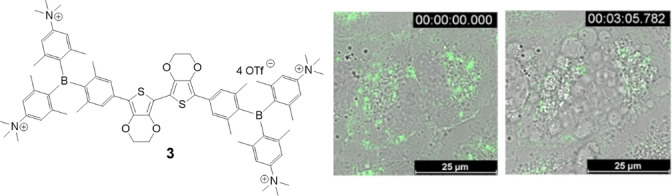

## Introduction

**Figure 1:**
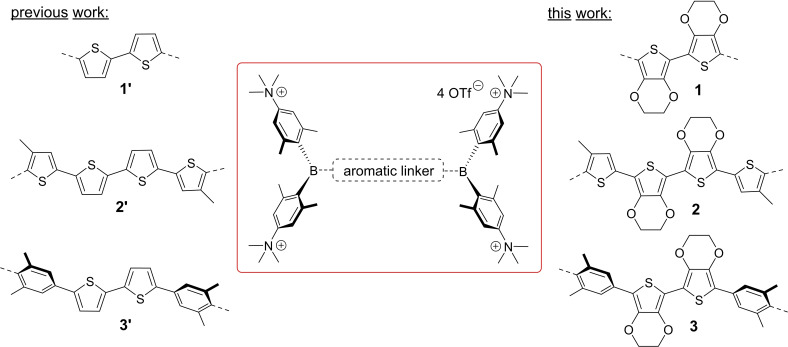
General structure of our water‐soluble A‐*π*‐A chromophores with different aromatic linkers including the target molecules **1**, **2**, and **3** of this study.

Increasing research interest has been focused on triarylboranes over the last three decades and the structural motif has been incorporated in numerous functional materials.[^1^, ^2^, ^3^, ^4^, ^5^, ^6^, ^7^, ^8^, ^9^, ^10^, ^11^] More recently, several triarylboryl‐containing chromophores have been successfully employed in different biological applications.[^12^, ^13^, ^14^, ^15^, ^16^, ^17^, ^18^, ^19^, ^20^, ^21^, ^22^, ^23^, ^24^, ^25^, ^26^, ^27^, ^28^, ^29^, ^30^, ^31^, ^32^, ^33^] The empty p_z_‐orbital at boron makes it a strong *π*‐acceptor and strong Lewis acid and needs to be protected sufficiently to obtain robust materials. A common way to achieve this is via kinetic stabilization by use of sterically demanding substituents, a concept first investigated by Krause and co‐workers.[^34^, ^35^, ^36^, ^37^] In 1957, Brown and Dodson reported BMes_3_ (Mes=mesityl=2,4,6‐trimethylphenyl) as the first air‐stable triarylborane^[38]^ and, later, it was found that, in many cases, two mesityl groups provide enough steric protection to obtain compounds that are stable under ambient conditions in the solid state and in typical organic solvents.[^39^, ^40^, ^41^, ^42^, ^43^, ^44^, ^45^, ^46^, ^47^, ^48^] Electronic effects also play an important role in the stability of triarylboranes. In 1955, Wittig and co‐workers reported tri‐4‐(*N*,*N*‐dimethylamino)phenylborane, lacking any steric protection from the 2‐ and 6‐ positions of the phenyl substituents, and found that it was air‐stable as a solid for ca. one week.^[49]^ The stability was explained by the electronic +M‐effect of the amine substituents. In contrast, when enhancing the Lewis acidity of boranes by introducing electron‐withdrawing groups, stability issues have to be carefully considered. The 2,4,6‐tris‐(trifluoromethyl)phenyl (^F^Mes) group was shown to be a convenient and very efficient aryl moiety that is both very strongly electron‐withdrawing and sufficiently bulky giving highly electron deficient and stable boranes.[^50^, ^51^, ^52^, ^53^] A systematic study by our group on donor‐acceptor thienyl‐BAr_2_ compounds demonstrated the influence of electron‐withdrawing groups on the reduction potential of the systems.^[54]^ For the thienyl‐BMes_2_ compound, sterically protected by four *ortho*‐methyl groups, a reduction potential of −2.23 V (*unless otherwise noted, all reduction potentials discussed are referenced* vs. *ferrocene/ferrocenium (Fc/Fc^+^)*) was reported, that is increased to −2.04 V by replacing the *para*‐methyl groups with either C_6_F_5_ or 3,5‐(CF_3_)_2_−C_6_H_3_.^[54]^ In the analogous thienyl‐B^F^Mes_2_ compound, the reduction potential was further increased to −1.61 V. Song and co‐workers used *para*‐cyano substituents in triarylboranes in which the boron was sterically protected by six *ortho*‐methyl groups, and reported reduction potentials of ca. −1.8 V.[^55^, ^56^] Gabbaϊ and co‐workers obtained water‐soluble and strongly Lewis acidic triarylboranes by stepwise substitution of the *para*‐methyl groups of BMes_3_ with trimethylammonium cations.^[57]^ The reduction peak potential was increased by 0.26 V for each substitution. Two substitutions were found to be sufficient to obtain a water‐soluble triarylborane with a reduction potential of −2.09 V. Despite its strong Lewis acidity, this compound was water‐stable, even in the low concentration range required for photophysical measurements. We combined this approach with our experience with acceptor‐*π*‐acceptor (A‐*π*‐A) chromophores[^58^, ^59^, ^60^] to design compounds with a (4‐(*N*,*N*,*N*‐trimethylammonio)‐2,6‐dimethylphenyl)_2_B‐(linker)‐B(4‐(*N*,*N*,*N*‐trimethylammonio)‐2,6‐dimethylphenyl)_2_ structural motif (Figure 1), and reported several chromophores of this type with applications in live‐cell imaging[^17^, ^24^, ^25^] and biomacromolecule sensing.[^30^, ^31^, ^32^] The boron atoms in our chromophores are strongly Lewis acidic, due to the electron‐withdrawing effect of the trimethylammonium cations, and thus need to be protected by six *ortho*‐methyl groups (e. g., compound **3’**, Figure 1) in order to be stable in pure water. In further studies, it was found that employing 9,10‐anthracenylene as the linker also provides a water‐stable compound, while the 1,4‐phenylene analogue decomposes within three hours in deuterated methanol under ambient conditions.^[24]^ When a thienyl group is directly adjacent to boron as part of the aromatic linker, the resulting compounds **1’** and **2’** were generally found to be air‐ and moisture‐stable in organic solvents.[^17^, ^24^] Stability was even sufficient for photophysical measurements in acetonitrile under ambient conditions over the course of one day. However, both compounds decompose too quickly at low concentrations in pure water to be applied in aqueous biological environments.

In this study, we substituted the two thiophenes in compounds **1’**, **2’**, and **3’** with similar, but more electron‐rich, 3,4‐ethylenedioxythiophene (EDOT) groups. As a monomeric building block of the widely used and commercially applied conductive polymer poly(3,4‐ethylenedioxythiophene):poly(styrenesulfonic acid) PEDOT:PSS,[^61^, ^62^, ^63^] EDOT itself, and oligomers thereof, are well investigated systems with several applications.[^64^, ^65^, ^66^, ^67^, ^68^, ^69^, ^70^, ^71^, ^72^, ^73^, ^74^, ^75^, ^76^] Compounds **1**, **2**, and **3** should be less Lewis acidic and, thus, possibly more stable than their respective analogues **1’**, **2’**, and **3’**, due to greater electron density at boron. As our group previously observed,^[60]^ a more electron‐rich *π*‐bridge in A‐*π*‐A chromophores is expected to result in significant bathochromic shifts in absorption and emission. The red and near infrared (NIR) region (600–1100 nm) is considered to be the “optically transparent window” of biological tissues and cells, as endogenous molecules do not absorb efficiently in this region.^[77]^ Thus, a more red‐shifted emission is generally a desirable feature for applications of chromophores in biological imaging. In addition, red‐emitting imaging agents are still rather rare, but are desirable for multiplex imaging.[^78^, ^79^]

## Results and Discussion

### Synthesis

**Scheme 1:**
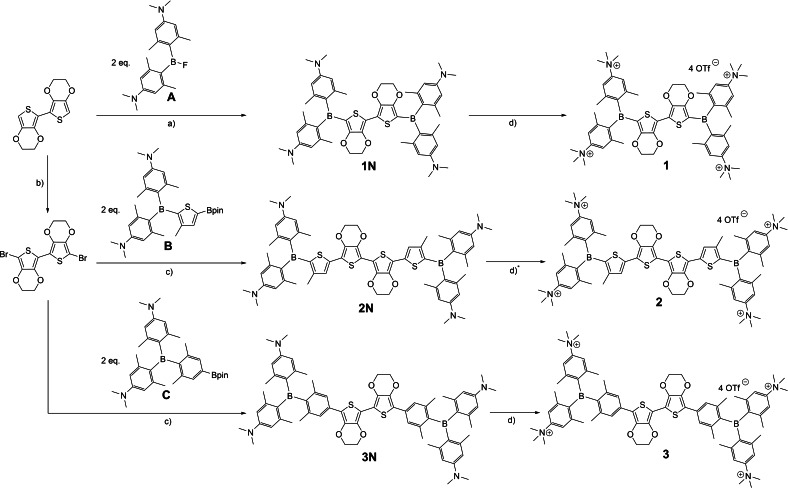
Synthesis of compounds **1N**, **2N**, **3N**, **1**, **2**, and **3**. a) *n*‐BuLi, THF, −78 °C to r. t.; b) NBS, CH_2_Cl_2_, −15 °C; c) Pd_2_(dba)_3_⋅CHCl_3_, SPhos, CsCO_3_, toluene/H_2_O (2/1), 85 °C; d) MeOTf, CH_2_Cl_2_, r. t.; d)* excess MeOTf, unidentified impurity in the compound, see Discussion for further information.

Compounds **1’**, **2’**, and **3’**, and the building blocks **A**, **B**, and **C** were previously reported.[^17^, ^24^] The compound 2,2’‐*bis*(3,4‐ethylenedioxythiophene) was synthesized by Ullman coupling of 2 equivalents of 3,4‐ethylenedioxythiophene according to the literature.^[64]^ Compound **1N** was synthesized by twofold lithiation of *bis*(3,4‐ethylenedioxythiophene) and subsequent reaction with **A** (Scheme 1). The bromination of *bis*(3,4‐ethylenedioxythiophene) with *N*‐bromosuccinimide to obtain 5,5’‐dibromo‐2,2'‐*bis*(3,4‐ethylenedioxythiophene) was attempted according to several literature procedures,[^66^, ^68^, ^80^] often affording an insoluble blue solid instead of the desired product. Based on these literature procedures, we report our optimized reaction conditions, yielding the product reproducibly in 91 % yield on a 2 mmol scale in the Supporting Information.

Compounds **2N** and **3N** were prepared via Suzuki‐Miyaura cross‐coupling reactions of 5,5’‐dibromo‐2,2’‐*bis*(3,4‐ethylenedioxythiophene) with the respective borylated triarylboranes **B** or **C**.[^17^, ^24^] Methylation of the neutral compounds **1N** and **3N** at the amine groups was performed according to our standard methylation protocol[^17^, ^24^, ^25^, ^26^] using 4.5 equivalents of MeOTf in CH_2_Cl_2_, with the tetracationic compounds **1** and **3** precipitating from the reaction mixture. Methylation of compound **2N** under the same conditions yielded a mixture of the twofold and threefold methylated compounds according to high resolution mass spectrometry (HRMS) studies of the precipitate. Performing the reaction in pure MeOTf or with nitromethane as the solvent led to decomposition of the starting material. Using 32 equivalents of MeOTf and a tenfold increase in the amount of solvent led to compound **2**, characterized by ^1^H NMR spectroscopy and HRMS, without any residual twofold or threefold methylated side product. However, an unidentified impurity [^1^H NMR (300 MHz, CD_3_OD): *δ*=4.6 ppm, presumably from decomposition due to excess MeOTf] could not be separated from the product. Therefore, our discussion of the properties of compound **2** will be kept to a minimum and should be treated with caution.

### Linear optical properties of and TD‐DFT calculations on the neutral precursors 1N, 2N, 3N

**Figure 2:**
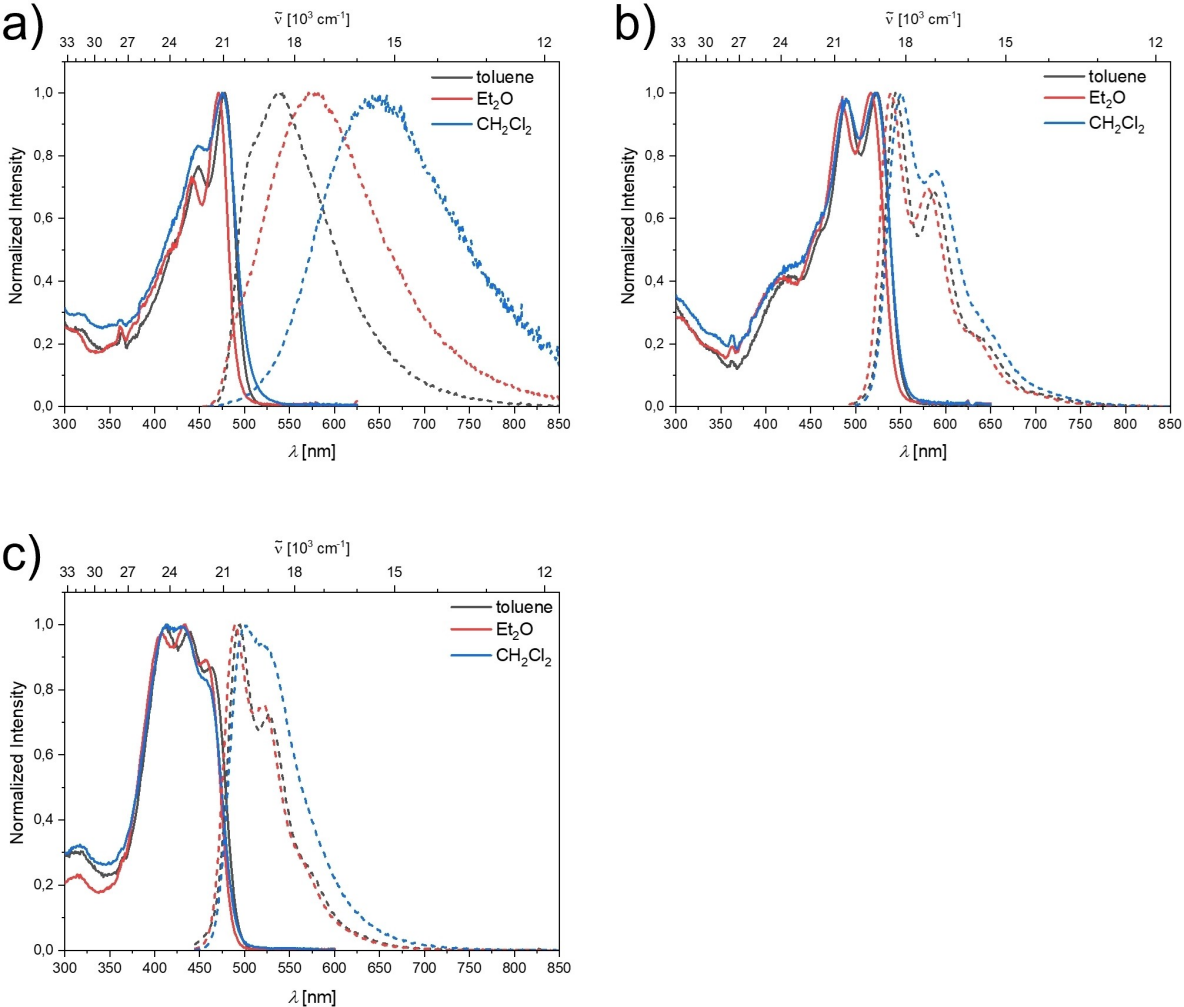
Absorption (solid line) and emission (dashed line) spectra of a) **1N**, b) **2N**, and c) **3N** in various solvents.

Photophysical data for compounds **1N**, **2N**, and **3N** were obtained in different solvents of increasing polarity (Figure 2). No significant dependence of the lowest energy absorption maxima on solvent polarity was observed for any of these compounds (Table 1), which indicates a weakly polarized ground state in all cases.

**Table 1:** Photophysical data for compounds **1N**, **2N**, and **3N** in various solvents.

	solvent	*λ*_abs_ [nm]	*ϵ* [M^−1^ cm^−1^]	*λ*_em_ [nm]	Stokes shift^[a]^ [cm^−1^]	*Φ*_f_	*τ* [ns]	*k*_r_ [10^8^ s^−1^]	*k*_nr_ [10^8^ s^−1^]
	toluene	478	65000	538	2300	0.16	2.0	0.8	4.3
**1N**	Et_2_O	471		578	3900	0.07	1.6	0.4	5.8
	CH_2_Cl_2_	475		650	5700	0.02	0.6	0.4	17.8
	toluene	524	81000	545	700	0.26	0.5	5.3	15.1
**2N**	Et_2_O	517		539	800	0.32	0.7	4.9	10.5
	CH_2_Cl_2_	522		550	1000	0.22	0.5	4.8	17.0
	toluene	463	61000	494	1400	0.17	0.3	6.8	33.2
**3N**	Et_2_O	457		490	1500	0.21	0.3	6.2	23.2
	CH_2_Cl_2_	456		501	2000	0.21	0.5	4.7	17.6

[a] apparent Stokes shift.

**Figure 3:**
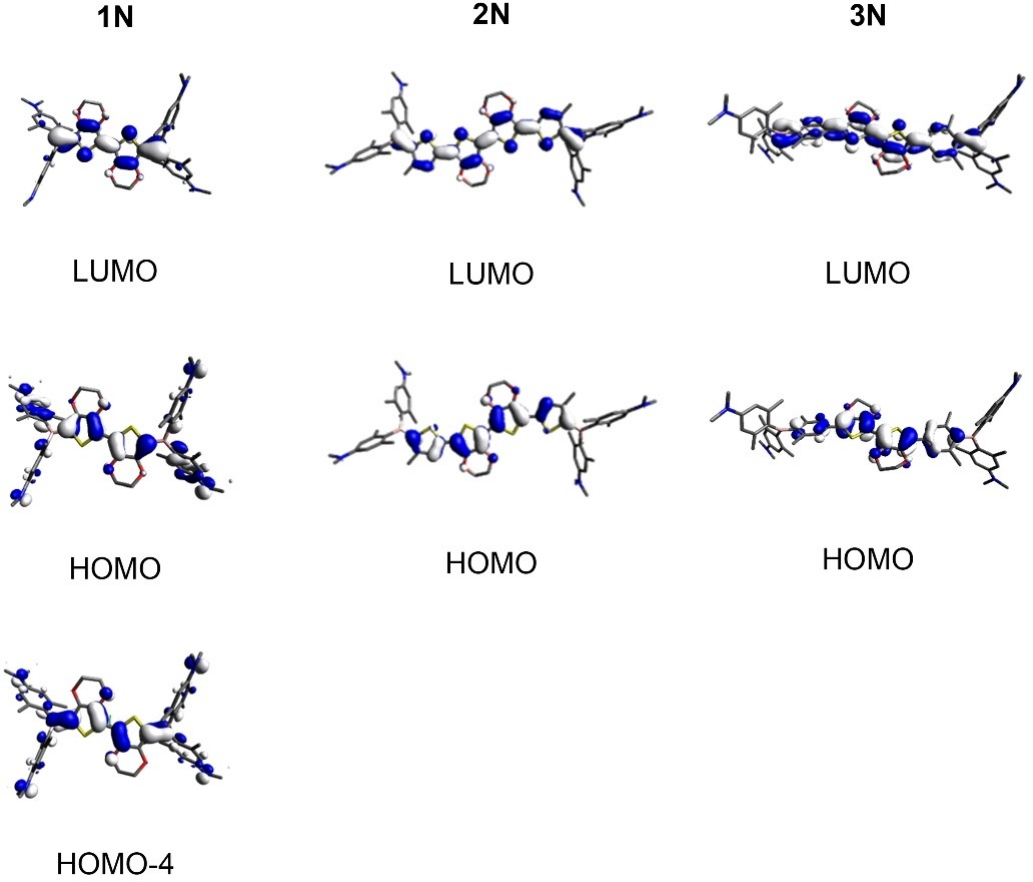
Orbitals relevant to the S_1_←S_0_ transition in toluene for compounds **1N**, **2N**, and **3N** calculated by TD‐DFT at the CAM−B3LYP/6‐31G+(d,p) level of theory.

TD‐DFT calculations were carried out for all molecules at the CAM−B3LYP/6‐31G+(d,p) level of theory (Supporting Information). In **1N** the S_1_←S_0_ transition (calculated in toluene at 422 nm and observed in toluene at 478 nm), can be attributed to a HOMO to LUMO transition with a minor contribution from HOMO‐4 to LUMO (Supporting Information, Tables S10 and S11). Both HOMO and HOMO‐4 are delocalized over the whole *π*‐system of the molecule, including the N,N‐dimethylamino‐2,6‐dimethylphenyl substituents (Figure 3). The LUMO is mainly located at the *π*‐bridge and the boron atoms. A moderate charge transfer (CT) character can thus be attributed to this transition [orbital overlap parameter (*Λ*)=0.63, with $\Lambda =\frac{\sum_{i,a}c_{i,a}^{2}\left\langle {|\phi }_{a}|||\phi_{i}|\right\rangle }{\sum_{i,a}c_{i,a}^{2}}$ resulting in 0≤*Λ*≤1, with 0 corresponding to no overlap and 1 to complete overlap].^[81]^ For most neutral precursors to our tetracationic A‐*π*‐A chromophores, the occupied molecular orbitals relevant to the S_1_←S_0_ transitions are localized only at the N,N‐dimethylamino‐2,6‐dimethylphenyl substituents and the lowest energy absorption corresponds to strong CT from the N,N‐dimethylamino‐2,6‐dimethylphenyl donor to the *π*‐bridge and the boron acceptor.[^25^, ^31^, ^32^] The S_1_←S_0_ transitions in compounds **2N** and **3N** (calculated in toluene at 482 nm and 424 nm and observed in toluene at 524 nm and 463 nm, respectively) can be attributed to HOMO to LUMO transitions in both cases (Supporting Information, Tables S12–S15). Both HOMO and LUMO are delocalized over the *π*‐bridges of the respective molecule (Figure 3) and the lowest energy transitions can therefore be classified as locally excited (LE) transitions (*Λ*=0.76 and 0.71, respectively). Similar behavior in neutral precursors to our tetracationic A‐*π*‐A chromophores has previously only been observed when the much larger (and in case of the former, strongly electron‐donating) bis(phenylthienyl)diketopyrrolopyrrol‐^[25]^ or bis(phenylethynyl)anthracene‐based^[32]^ *π*‐systems were employed as linkers.

The emission maximum of compound **1N** shifts bathochromically with increasing solvent polarity, which results in an increase of the apparent Stokes shift from 2300 cm^−1^ in toluene to 3900 cm^−1^ in Et_2_O, and 5700 cm^−1^ in CH_2_Cl_2_. This positive solvatochromism with increasing solvent polarity suggests large (local) dipole moments in the excited state and is in accordance with the moderate CT character of the lowest energy absorption band determined for this compound. In accordance with a stronger CT character of the S_1_←S_0_ transitions of most analogous, neutral A‐*π*‐A chromophores, the Stokes shifts in these analogues are ca. 1500 to 2000 cm^−1^ larger in each solvent than those of **1N**.[^25^, ^31^, ^32^] The fluorescence quantum yields of **1N** decrease with increasing solvent polarity from 0.16 in toluene to 0.07 in Et_2_O, to 0.02 in CH_2_Cl_2_. Together with increased nonradiative decay rate constants (Table 1), this trend is consistent with the energy gap law,^[82]^ which dictates more efficient internal conversion processes as the energy gap between excited and ground state becomes smaller. Thus, in accordance with the LE character of the lowest energy transitions for **2N** and **3N**, no significant dependence of emission maxima on solvent polarity was observed and the fluorescence quantum yields in all solvents range from 0.2 to 0.3 (Table 1).

### Linear optical properties of and TD‐DFT calculations on the tetracations 1, 2, 3

**Figure 4:**
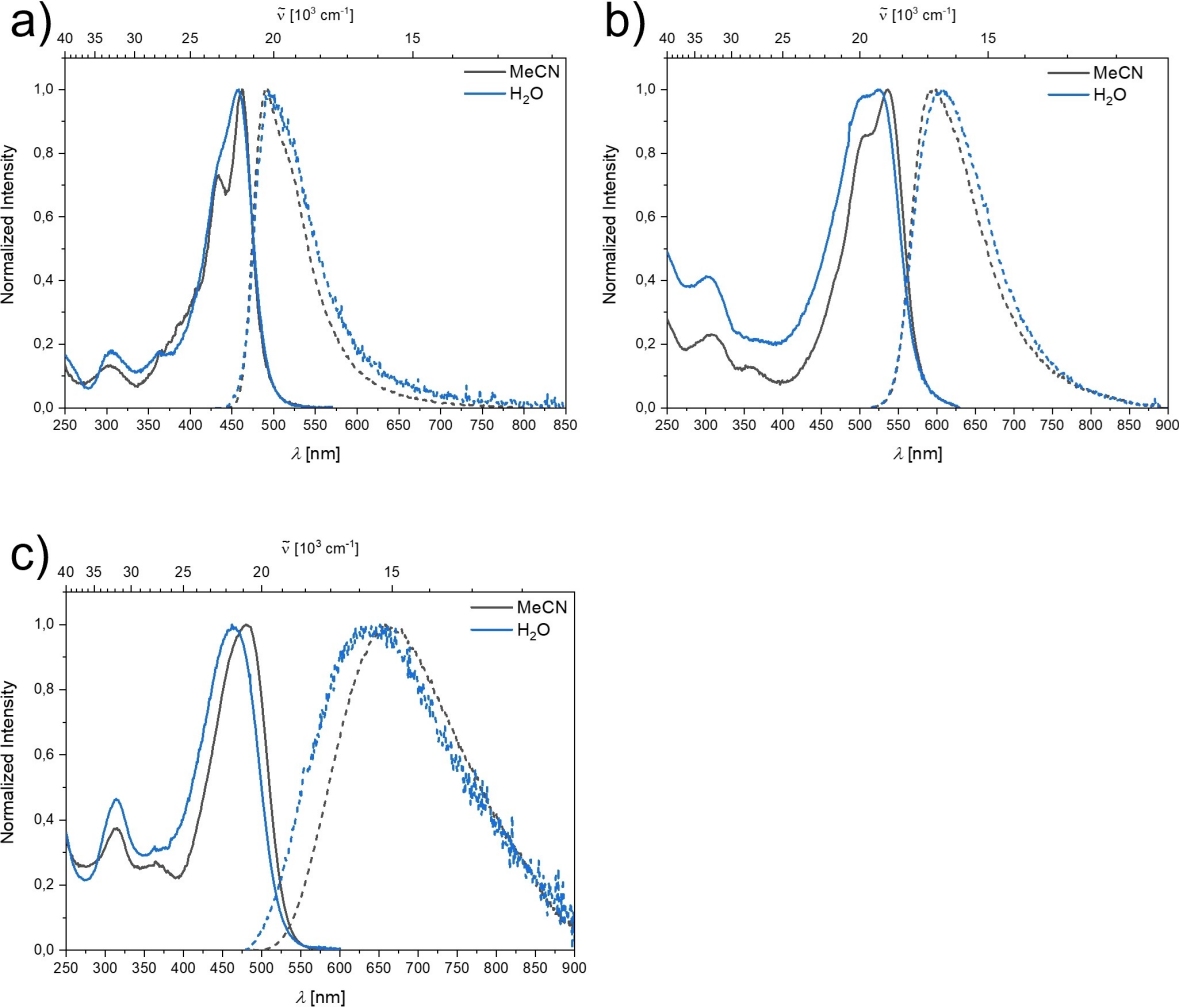
Absorption (solid line) and emission (dashed line) spectra of a) **1**, b) **2**, and c) **3** in various solvents.

**Figure 5:**
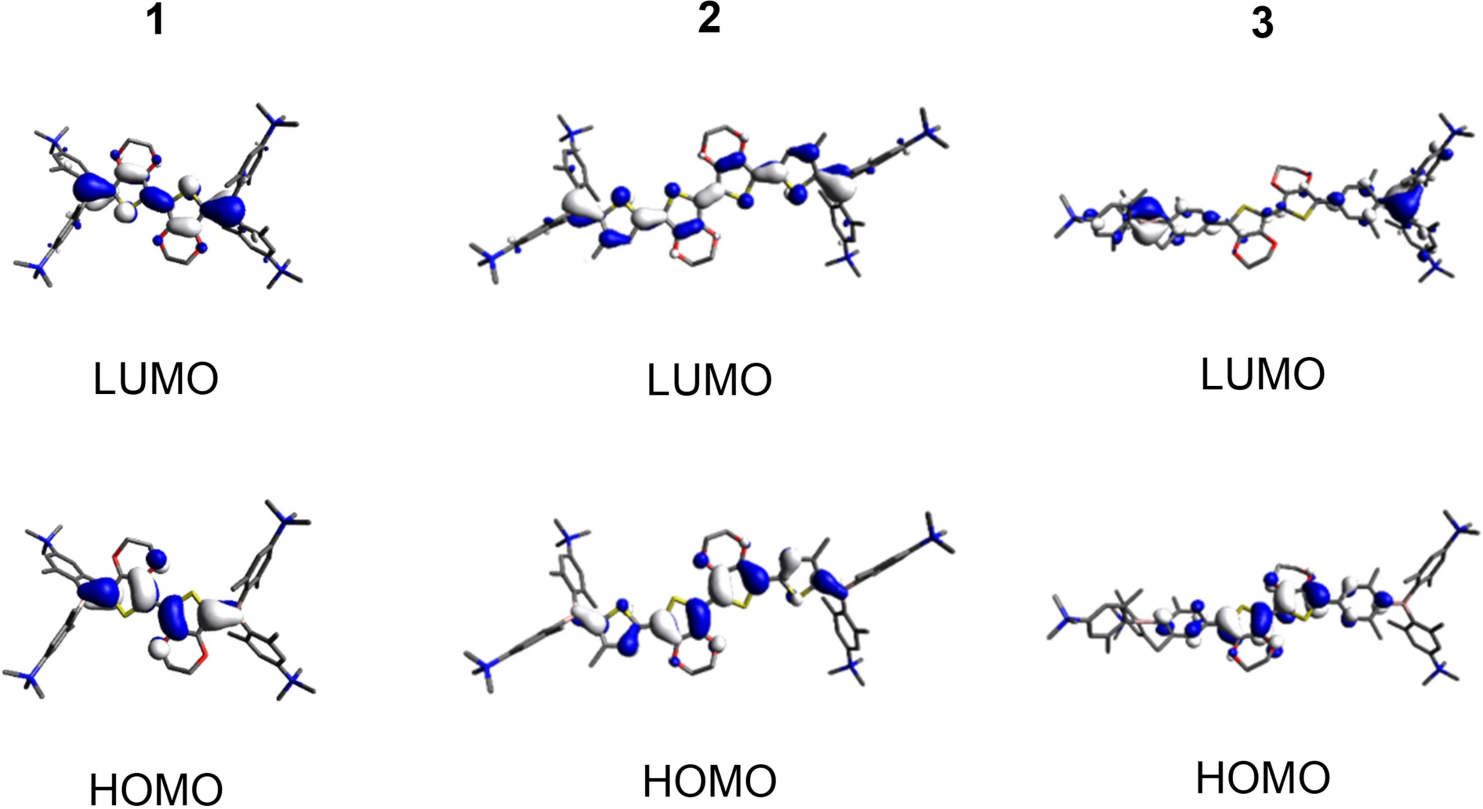
Orbitals relevant to the S_1_←S_0_ transition in MeCN for compounds **1**, **2**, and **3** calculated by TD‐DFT at the CAM−B3LYP/6‐31G+(d,p) level of theory.

Upon methylation of the neutral compounds at the four dimethylamino groups, electron donation from amine to boron is no longer possible and, thus, the acceptor strength of boron is increased and the resulting salts become water‐soluble. Photophysical data for compounds **1**, **2**, and **3** were obtained in MeCN and water (Figure 4). TD‐DFT calculated data suggest, in all cases, that the S_1_←S_0_ transitions are attributed to HOMO to LUMO excitations. HOMO and LUMO of compounds **1** and **2** are delocalized over the respective *π*‐bridges and the boron atoms (Figure 5) and the transitions can thus be classified as LE *π*‐π* transitions (*Λ*=0.71 and 0.67, respectively). The situation is similar for **3**; however, due to the twist introduced by the sterically bulky dimethylphenyl group, the orbital distribution is uneven over the bridge. As the HOMO is mostly located at the EDOT and the LUMO at boron, the S_1_←S_0_ HOMO to LUMO transition of **3** has slight CT character (*Λ*=0.47). The observed trend that the lowest energy absorption energy decreases in the order **1**>**3**>**2** (i. e., observed in MeCN: 462, 482, 536 nm, respectively (Table 2)) is well reproduced by the TD‐DFT calculated data, when solvent effects are included (i. e., calculated in MeCN: 418, 459, 509 nm, respectively (Supporting Information, Tables S17, S19, S21)). As **3** exhibits a higher degree of CT character than **1** and **2**, the influence of the solvent on the transition is more pronounced for **3**, and calculations including solvent correction reproduce the experimental data more accurately.

**Table 2:** Comparison of photophysical data for compounds **1**, **2**, **3** and compounds **1’**, **2’**, **3’**[^17^, ^24^] in MeCN and water.

	solvent	*λ*_abs_ [nm]	*λ*_em_ [nm]	Stokes shift^[a]^ [cm^−1^]	*Φ*_f_
**1**	MeCN	462	492	1300	0.03
	H_2_O	458	495	1600	–^[b]^
**1’**	MeCN	426	448	1200	0.41
	H_2_O	426	451	1300	–^[b]^
**2^c^**	MeCN	536	601	2000	–^[c]^
	H_2_O	526	614	2700	–^[b,c]^
**2’**	MeCN	464	558	3600	0.27
	H_2_O	463	570	4100	0.21
**3**	MeCN	482	662	5600	0.07
	H_2_O^a^	465	651	6100	0.01
**3’**	MeCN	428	554	5300	0.41
	H_2_O^[a]^	425	563	5800	0.10

[a] apparent Stokes shift; [b] not measurable due to rapid decomposition; [c] for **2**, only the spectra are given and they should be interpreted with care due to an unidentified impurity. For the same reason, measurements of *Φ*_f_ were not performed.

The emission maxima of **1**, **2**, and **3** do not shift significantly upon changing the solvent from MeCN to water. The most notable solvent effect is a broadening of the emission spectra in the more polar solvent water for all three compounds as was previously observed for similar systems.^[25]^ In accordance with the slight CT character of the lowest energy transition and suggested (locally) polarized excited state for **3**, large Stokes shifts of ca. 6000 cm^−1^ are observed for this compound in MeCN and water (Table 2), whereas for **1** and **2** they are ca. 1500 and 2500 cm^−1^, respectively.

Due to the large Stokes shift, the emission of **3** [*λ*_em_ (MeCN)=662 nm; *λ*_em_ (H_2_O)=651 nm] is even more bathochromically shifted than that of its bis(phenylthienyl)diketopyrrolopyrrol analogue [*λ*_em_ (MeCN)=617 nm; *λ*_em_ (H_2_O)=620 nm]^[25]^ and is the most redshifted of all tetracationic water‐soluble bis‐triarylborane chromophores reported to date. However, the redshift comes at the expense of the fluorescence quantum yield,^[82]^ which was determined to be 0.07 in MeCN and 0.01 in water.

In Table 2, the photophysical data for EDOT‐containing compounds **1**, **2**, and **3** are compared with their thiophene‐containing analogues **1’**, **2’**, and **3’**. As expected for A‐*π*‐A chromophores,^[60]^ the introduction of a more electron rich *π*‐bridge leads to a bathochromic shift in absorption and emission in all cases. However, as dictated by the energy gap law^[82]^ (see above), a significant decrease of fluorescence quantum yield is also observed in all cases in which fluorescence quantum yield could be measured.

### Cyclic voltammetry and stability

The red‐shifted lowest energy absorption and emission maxima of compounds **1**, **2**, and **3**, compared to **1’**, **2’**, and **3’**, respectively, are consistent with the stronger electron‐donating effect of EDOT compared to thiophene in our systems. As a greater electron density at the boron should result in increased stability against nucleophilic attack, we investigated to what extent the substitution of thiophene by EDOT increases the electron density at the boron centers. A common way to quantify this is by measuring the reduction potential via cyclic voltammetry.[^57^, ^83^, ^84^] All cyclic voltammograms were obtained in MeCN and are referenced to the Fc/Fc^+^ ion couple. Half‐wave reduction potentials were measured for all six compounds even though their reduction events are not always fully chemically reversible. In particular, reductions of the sterically less protected compounds **1**, **1’**, **2**, and **2’** are accompanied by irreversible processes (Supporting Information, Figures S14–S19). In the cyclic voltammograms of **1** vs. **1’**, with comparatively shorter *π*‐bridges, two half‐wave reduction potentials were observed at −1.59 V and −1.93 V vs. −1.46 V and −1.79 V, respectively (Table 3), indicating a pronounced electronic communication between the two boron atoms. For the larger analogues **2** vs. **2’** (−1.78 V vs. −1.71 V) and **3** vs. **3’** (−1.97 V vs. 1.92 V), only one half‐wave reduction potential was observed for each compound, suggesting inefficient communication between the two boron atoms. Taking each pair (i. e., **1** vs. **1’**, **2** vs. **2’**, **3** vs. **3’**) into account, a consistent but rather small shift to more negative reduction potentials is observed for the respective EDOT‐containing compounds when compared with their thiophene analogues.

**Table 3:** Half‐wave potentials of partially reversible reduction processes of compounds **1**, **1’**, **2**, **2’**, **3**, and **3’**. All measurements were performed in acetonitrile with [nBu_4_N][PF_6_] as the electrolyte with a scan rate of 250 mV s^−1^ and are referenced to the Fc/Fc^+^ ion couple.

	**1**	**1’**	**2**	**2’**	**3**	**3’**
1^st^ reduction: *E*_1/2_ [V] vs. Fc/Fc^+^	−1.59	−1.46	−1.78	−1.71	−1.97	−1.92
2^nd^ reduction: *E*_1/2_ [V] vs. Fc/Fc^+^	−1.93	−1.79	–	–	–	–

**Figure 6:**
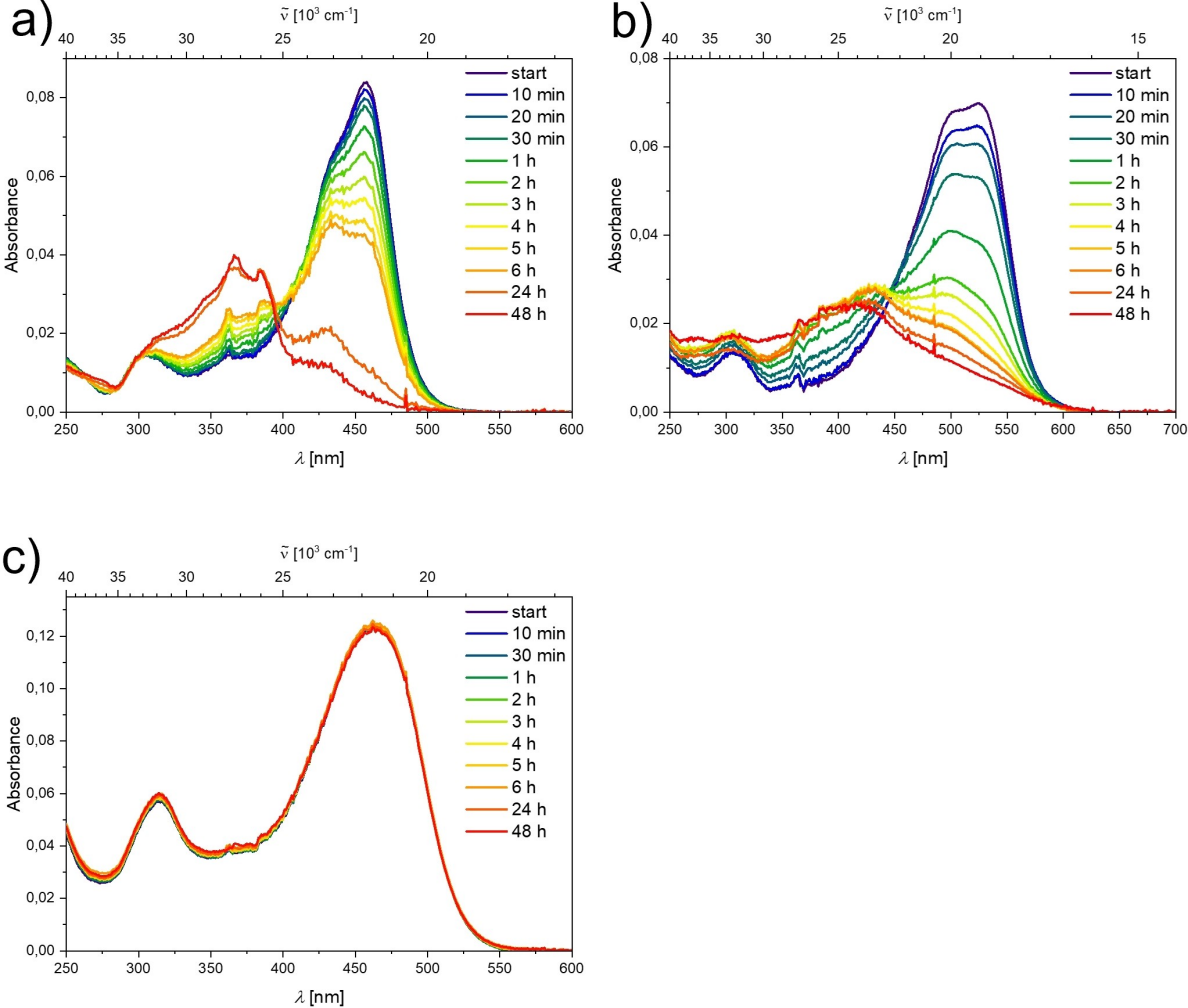
UV/Vis spectra of solutions of a) **1**, b) **2** and c) **3** over a period of 48 h in water. Preparation of stock solutions with the same concentrations of **1**, **2**, and **3** was not possible, as the dilution takes time and a measurement of absorption spectra at *t*=0 min would, thus, not be possible.

The three EDOT‐containing compounds **1**, **2**, and **3** are air and moisture stable in the solid state for several months and are also stable during photophysical measurements at low concentrations in MeCN solutions under ambient conditions, as are their thiophene analogues **1’**, **2’**, and **3’**. However, compounds **1’** and **2’** decomposed quickly in pure water at low concentrations, while **3’** shows no sign of decomposition over a period of 48 h.[^17^, ^24^] Thus, water‐stability was also examined for **1**, **2**, and **3** via UV/Vis spectroscopy at low concentrations (Figure 6). As the sterically less protected compounds **1** and **2** start to decompose within minutes, it is concluded that the slight increase in electron density at the boron does not significantly affect their water‐stability. Only the sterically most protected compound **3** is stable over a period of 48 h.

### Reactivity with oxygen

After *α*‐terthienyl had been identified as the nematicidally‐active species in Tagetes (African marigold) roots,^[85]^ it was demonstrated that its nematicidal activity increases under UV‐light irradiation.^[86]^ Later, it was found that the nematicidal activity of *α*‐terthienyl is due to singlet oxygen generated upon UV‐light irradiation.^[87]^ Since then, it has been well established that thiophene‐ and EDOT‐containing compounds are able to generate singlet oxygen via efficiently populated triplet states.[^71^, ^74^, ^88^, ^89^, ^90^, ^91^] In addition, we recently reported persistent room temperature phosphorescence from simple triarylboranes, sensitive to oxygen quenching in the solid state.^[92]^

The photosensitized production of singlet oxygen via organic molecules has been a very pertinent research field for many decades.[^93^, ^94^, ^95^, ^96^, ^97^, ^98^, ^99^, ^100^, ^101^, ^102^, ^103^, ^104^, ^105^, ^106^] Singlet oxygen has found many applications,^[107]^ for example, as a synthetic reagent,^[108]^ an insecticide^[109]^ and, perhaps most prominently, as a reactive oxygen species (ROS) leading to cell death in unwanted tissue via photodynamic therapy.[^110^, ^111^, ^112^] Still, the design of water‐soluble and biocompatible singlet oxygen‐generating molecules remains a challenge.[^113^, ^114^, ^115^, ^116^] We recently found that the cytotoxicity of several cationic bis‐triarylborane chromophores is significantly increased upon irradiation, presumably due to singlet oxygen formation inside the cells.^[33]^ As **3’** was shown to be water‐soluble, water‐stable and biocompatible,^[17]^ we examined this compound and its water‐soluble and water‐stable analogue **3** for the sensitization of singlet oxygen.

**Figure 7:**
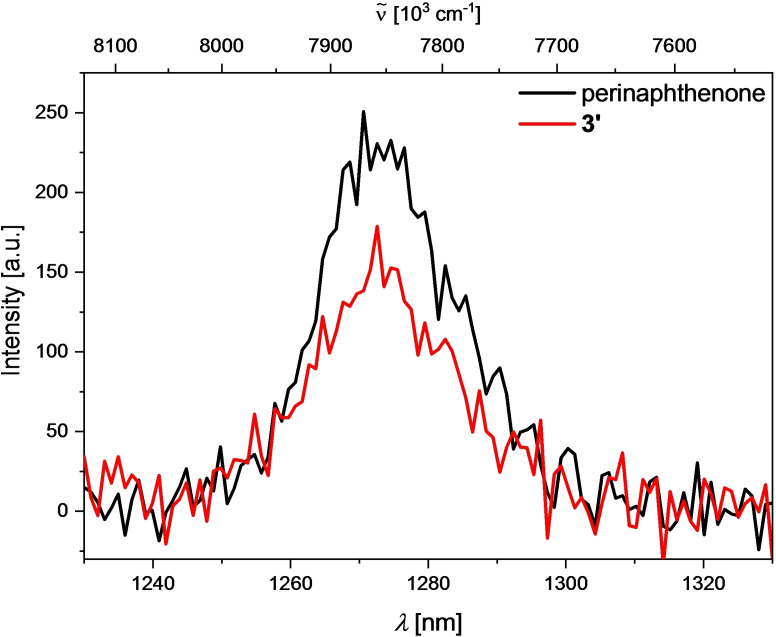
Emission spectrum of singlet oxygen generated from sensitization of a perinaphthenone standard (black) vs. that generated by sensitization by compound **3’** (red) excited at 404 nm in MeCN.

The presence of singlet oxygen in solution can be observed and quantified by its luminescence at 1272 nm.[^104^, ^117^] Upon excitation of an O_2_‐saturated solution of compound **3’** in MeCN, emission at 1272 nm was detected (Figure 7).

The quantum yield for singlet oxygen formation (*Φ*_Δ_) (Table 4) was determined to be 0.6 by comparison to the standard perinaphthenone which is known to sensitize singlet oxygen with an efficiency close to unity in MeCN.^[100]^ A comparatively small radiative rate constant of 0.45 s^−1[118]^ for the luminescence of singlet oxygen at 1272 nm in MeCN explains the very low signal to noise ratio obtained. A measurement of singlet oxygen generation in water was not possible, as an even lower radiative rate constant of 0.16 s^−1[118]^ in water precluded detection of singlet oxygen luminescence with our experimental setup, even after sensitization by perinaphthenone.

**Table 4:** Fluorescence quantum yield (*Φ*_f_)^[17]^ and quantum yield for singlet oxygen formation (*Φ*_Δ_) of compound **3’** in MeCN.

	*Φ*_f_	*Φ*_Δ_^[a]^
**3’**	0.41	0.6

[a] due to the very low signal to noise ratio an error of ±0.1 is assumed.

**Figure 8:**
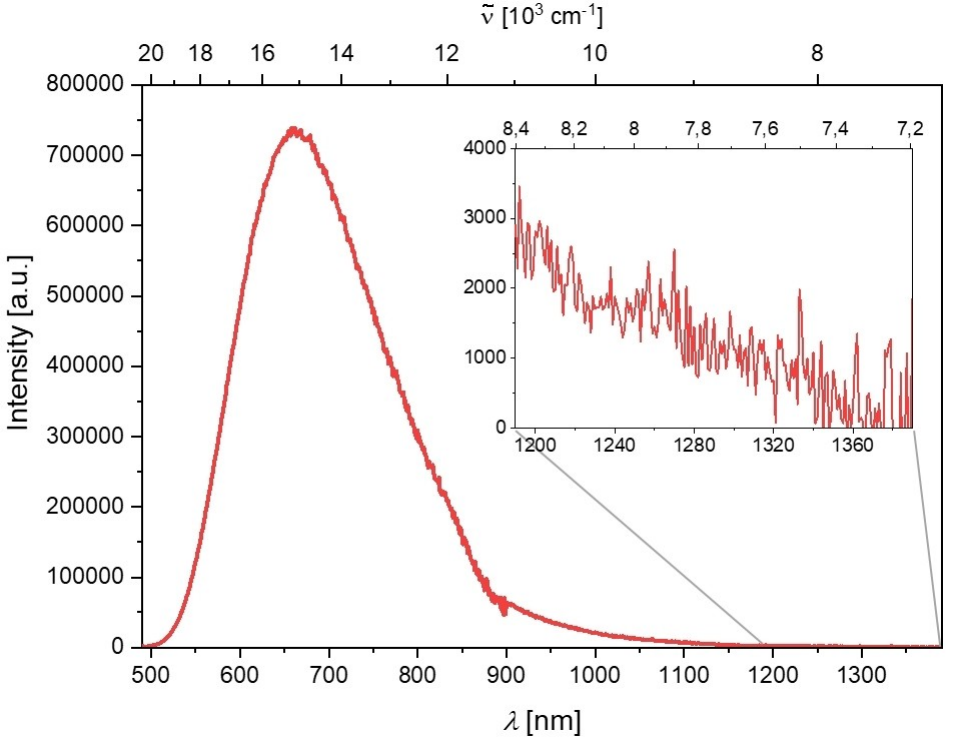
Emission spectrum of compound **3** in MeCN; insert: enlarged section from the spectrum, showing detectable emission until ca. 1360 nm.

For compound **3**, the detection of singlet oxygen luminescence was not possible. The emission of the compound is bathochromically shifted compared to **3’** with a maximum at 662 nm in MeCN. However, due to its very broad emission band, it is still detectable well into the NIR range, possibly overlapping with the weak phosphorescence of singlet oxygen (Figure 8). An attempt to time‐gate the detection, to exclude the short‐lived fluorescence, using a μF920 pulsed 60 W Xenon microsecond flashlamp for excitation, gave no significant signal at 1272 nm even after sensitization by perinaphthenone, presumably due to an even lower signal to noise ratio.

**Figure 9:**
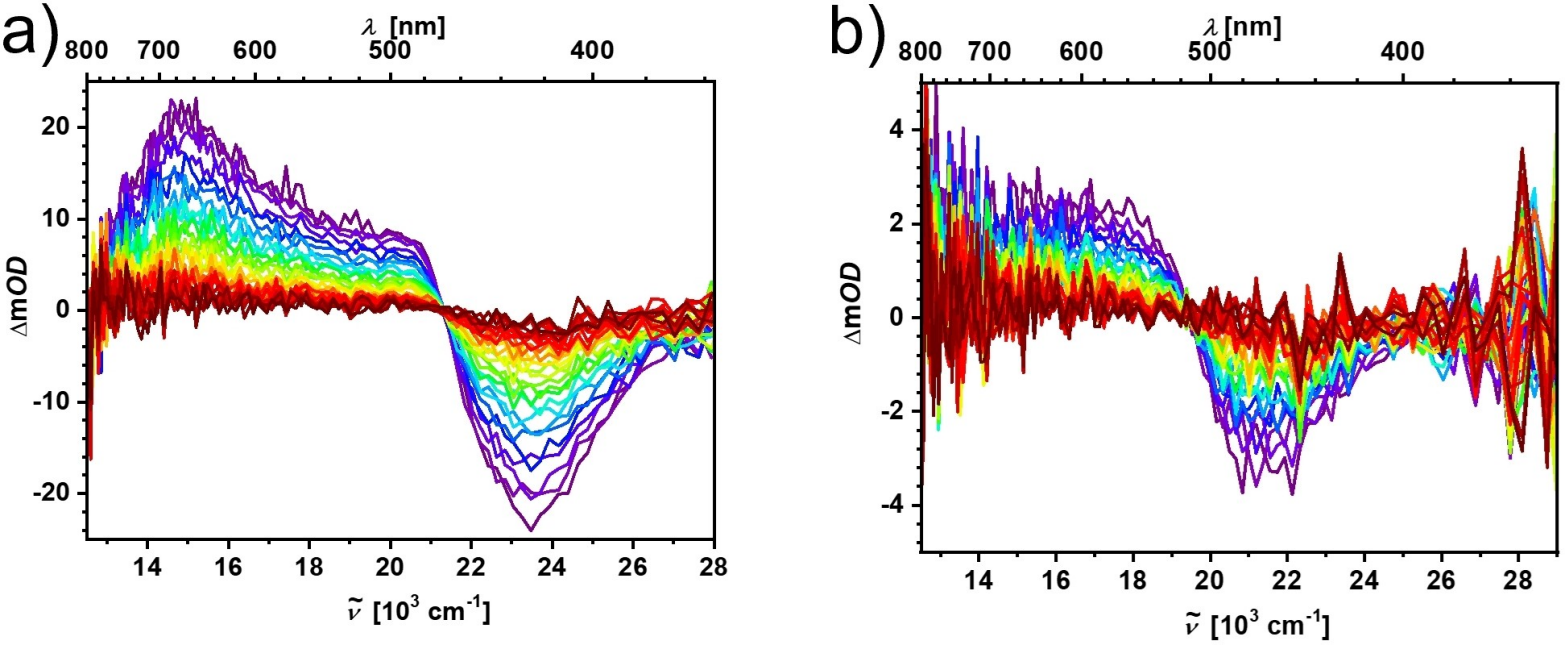
Nanosecond transient absorption spectra of a) 3’ and b) 3 in degassed MeCN solutions.

To confirm the population of possible triplet states, we performed transient absorption measurements on both **3** and **3’**. In both cases, excited state absorption was observed (Figure 9). In case of **3’** the long‐lived excited state absorbs in the range of 480 nm to 800 nm and has a lifetime of 94.6 μs (Supporting Information Figure S20). The absorbance of the long‐lived state of **3**, with a lifetime of 105 μs (Supporting Information Figure S21), is much weaker. The absorbance is observable in the range of 520 nm to 620 nm, but is difficult to distinguish from noise at wavelengths larger than that. Both long‐lived states are completely quenched when oxygen is introduced into the solutions.

### Study of interactions with DNA, RNA, and protein

As we previously reported that the close analogue **3’** strongly interacts with DNA/RNA and protein^[30]^ and confirmed in further studies that tetracationic bis‐triarylboranes, in general, possess excellent DNA/RNA sensing capabilities,[^31^, ^32^, ^33^] we also studied the interactions of **3** with some biorelevant targets, i. e., *ct*‐DNA as an example double stranded (ds) DNA, pApU as an example ds‐RNA, and BSA as an example protein.

**Figure 10:**
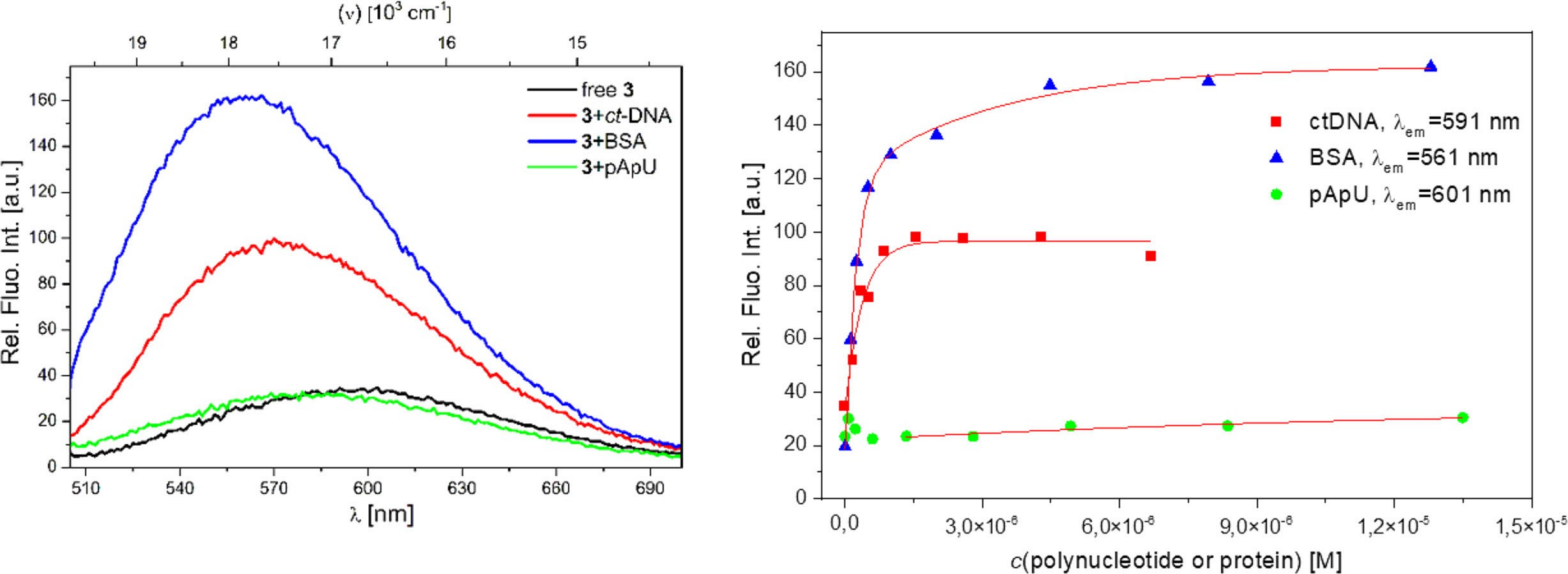
a) Comparison of the last titration points in fluorescence titrations of **3** (*c*=5.0×10^−8^ M) with *ct*‐DNA (*c*=6.7×10^−6^ M), BSA (*c*=1.3×10^−5^ M) and pApU (*c*=1.4×10^−5^ M) at *λ*_exc_=471 nm. b) Changes in fluorescence of **3** (*λ*_exc_=471 nm, *c*=5.00×10^−8^ M) upon addition of polynucleotides and BSA. All measurements were made at pH 7.0 in sodium cacodylate buffer, *I*=0.05 M.

**Figure 11:**
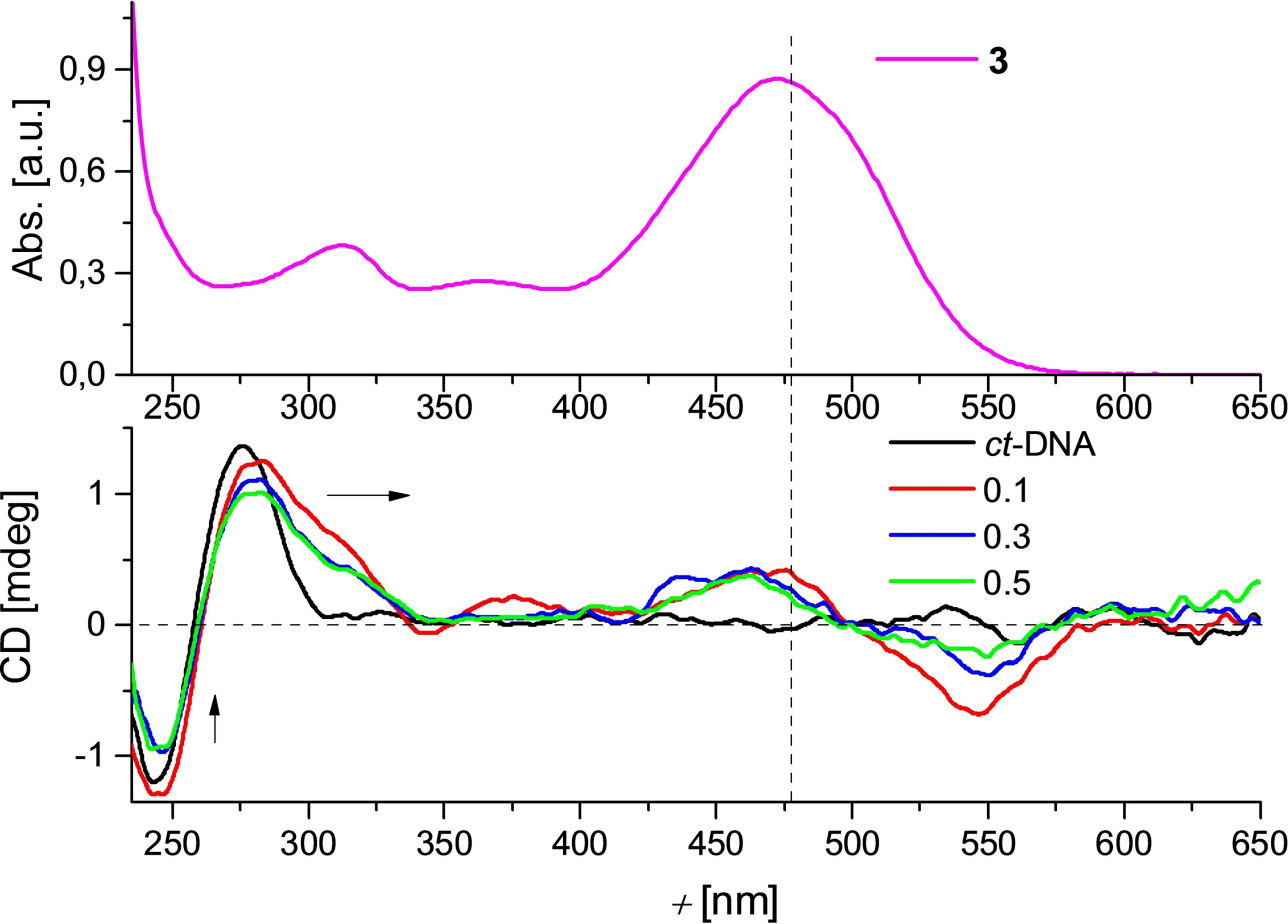
CD titrations of *ct*‐DNA (*c*=2×10^−5^ M) at various ratios *r* [dye]/[polynucleotide] with **3** (bottom). UV‐Vis spectum of **3** (*c*=1.8×10^−6^ M) (top). Done at pH 7.0 in sodium cacodylate buffer, *I*=0.05 M.

Thermal denaturation experiments (Supporting Information, Figures S29 and S30), fluorimetric titrations, (Figure 10, Supporting Information, Figures S25–S27) and CD titrations (Figure 11, Supporting Information, Figure S28) showed that **3** binds strongly to ds‐DNA and BSA, but only negligibly to ds‐RNA, thus showing some distinct differences with respect to parent compound **3’** (Table 5).

**Table 5:** Binding constants^[a]^ (log *Ks*) of **3** and **3’** ^[30]^ with ds‐polynucleotides and BSA calculated by processing fluorimetric titrations and Δ*T*_m_ – values^[c]^ (°C) upon **3** and **3’** ^[30]^ added to ds‐polynucleotides. All measurements were made at pH=7.0 in sodium cacodylate buffer, *I*=0.05 M.

	*ct*‐DNA		pApU		BSA
	log *Ks*	Δ*T*_m_	log *Ks*	Δ*T*_m_	log *Ks*
**3’** ^[30]^	7.0	+7.3	7.0	+9.5	5.9
**3**	7.0	+7.3	–^[b]^	0	5.5

[a] Analyses of titration data by means of the Scatchard equation^[119]^ with von Hippel formalism^[120]^ gave values of the ratio *n*=[bound compound]/[polynucleotide]=0.2–0.3; for easier comparison, all log *Ks* values were re‐calculated for fixed *r*=0.25 (ds‐polynucleotides). Correlation coefficients were >0.99 for all calculated *Ks* values; [b] Negligible emission change did not allow determination of the binding constant; [c] Measurements were made at ratio *r*_[compound]/[polynucleotide]_=0.2; Error of ▵*T*_m_=±0.5 °C.^[121]^

Fluorescence of **3** is strongly enhanced upon binding to ds‐DNA and BSA, but in contrast to **3’**, only changed negligibly upon addition of ds‐RNA (Figure 10). Also, addition of **3** stabilized only ds‐DNA but not ds‐RNA against thermal denaturation, thus leading to the conclusion that **3** binds to ds‐DNA with high selectivity in comparison to ds‐RNA, while **3’** efficiently binds to ds‐DNA, ds‐RNA, and protein (Table 5). This selectivity could be attributed to the higher steric hindrance of the bis‐EDOT bridge of **3** compared to the bis‐thiophene bridge of **3’**, which prevents efficient insertion into the narrow major groove of ds‐RNA (width 3.8 Å, Supporting Information, Table S9), while still inserting efficiently into the minor groove of ds‐DNA (width 6.3 Å, Supporting Information, Table S9).

Another difference between **3’** and **3** is the loss of a fluorimetric response which allows a simultaneous determination of the concentrations of ds‐DNA and BSA in solution by ratiometric analysis in the case of **3’** (see Figures 1 and 2 in Ref. [30]). In the case of **3**, the emission changes are too similar between DNA and protein.

The structural aspects of the binding of **3** to ds‐DNA/RNA were studied by circular dichroism (CD) experiments. CD spectra are highly sensitive to changes in the secondary structure of DNA and RNA^[122]^ and induced (I)CD signals of intrinsically achiral small molecules may be observed when those are bound to chiral hosts (DNA and RNA).^[123]^ Upon binding to *ct*‐DNA, **3** revealed strong bisignate ICD bands at wavelengths >300 nm, with the zero point slightly bathochromically shifted with respect to the absorption maximum of pure **3** (Figure 11). This suggests a well‐defined orientation of **3** with respect to the chiral axis of DNA.^[123]^ Similar observations were made for compound **3’**.^[30]^ Such ICD bands were not observed upon addition of **3** to ds‐RNA (Supporting Information, Figure S28). These observations additionally suggest an efficient insertion of **3** only into the minor groove of ds‐DNA, but not into any groove of ds‐RNA.

### Cell studies

As a strong interaction of small organic molecules with DNA or proteins can cause strong cytotoxic effects and, also, as the strong increase of the emission of **3** upon binding to DNA and BSA makes it a promising candidate for a fluorimetric marker, we performed a series of experiments on a human cell line.

**Figure 12:**
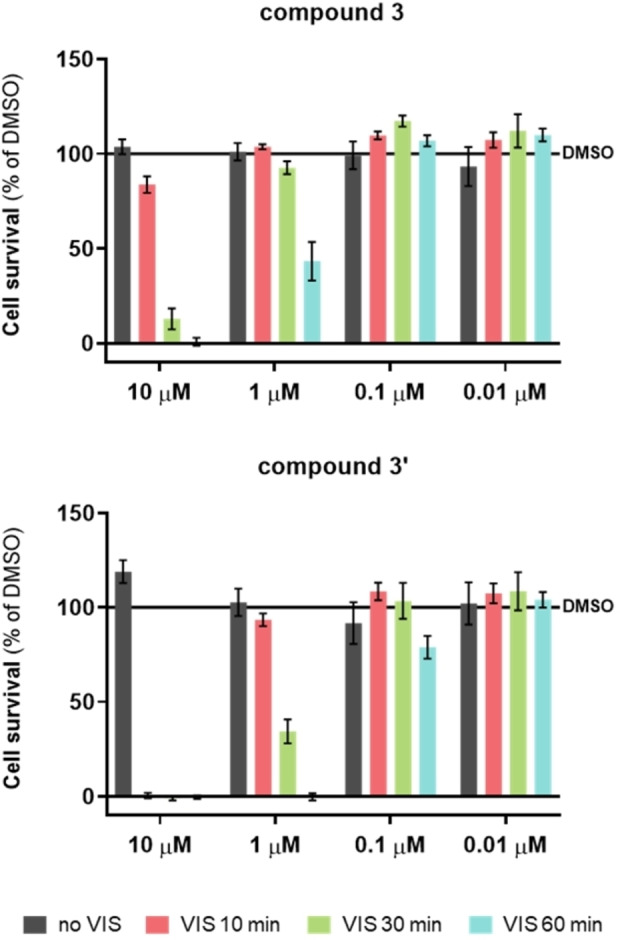
Cell survival of A549 cells exposed to compounds **3** (top) and **3’** (bottom), with or without exposure to visible light irradiation. Irradiation occurred in a Luzchem reactor with visible light range 90 min after addition of dye (400–700 nm, 8 lamps, in total 56 W, Dose 50.6 mw m^−2^), 18 cm lamp to cell‐plate, for 10, 30, or 60 min, and then left in the incubator overnight (37 °C, 5 % CO_2_). Irradiation was performed for three subsequent days at the same time point each day. Data are presented as mean±SD made in four replicates, relative to the control samples (DMSO). Representative data from three independent experiments yielding similar results are shown.

The cytotoxicity of EDOT compound **3** and, for comparison, also that of thiophene compound **3’** was tested using the MTT assay against the human lung carcinoma (A549) cell line (Figure 12). In the dark, **3** and **3’** are negligibly cytotoxic at all concentrations tested (0.01, 0.1, 1.0, and 10 μM). However, when irradiating the incubated cells in the presence of **3** or **3’** at 400–700 nm for 10 min, the viability of the cells was reduced at the highest concentration in both cases. Prolonged irradiation (30 and 60 min) yielded increasing bioactivity even at 1 μM concentration, whereby the effect of **3’** is stronger when compared to **3**.

The increased toxicity of these compounds when irradiated with visible light results most likely from their ability to sensitize singlet oxygen (see above, Figures 7, 8, 9) as this ability can lead to in vivo production of ROS species leading to cell death.^[104]^

**Figure 13:**
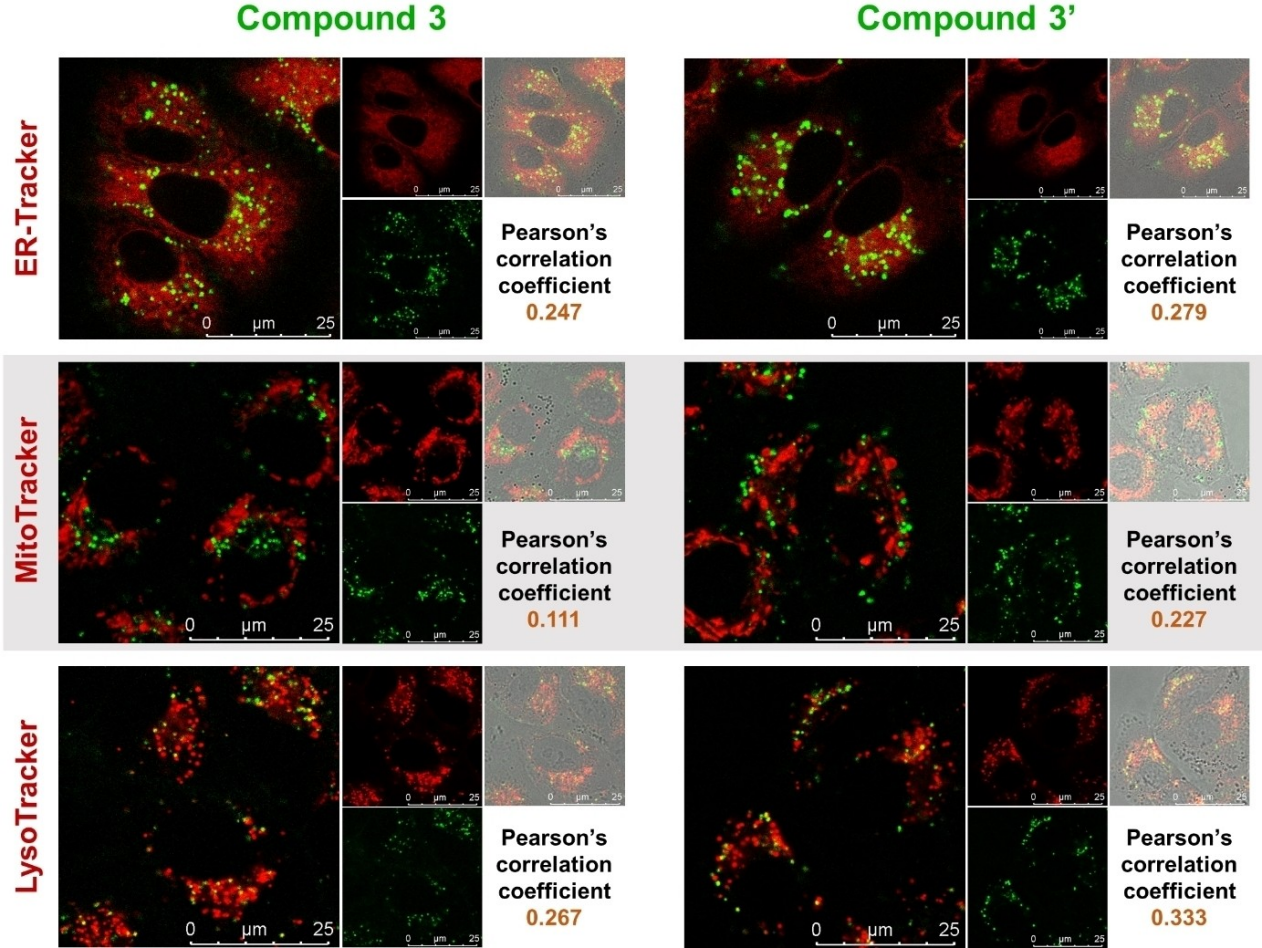
Intracellular localization of **3** or **3’** (shown in green; *λ*_exc_=457 nm, *λ*_em_=500–600 nm) at concentrations of 10 μM for 90 min at 37 °C in A549 cells. Colocalization with endoplasmic reticulum (ER‐Tracker), mitochondria (MitoTracker) or lysosomes (LysoTracker), all shown in red, was monitored by confocal microscopy. Merged signals with white field are shown in gray.

Confocal microscopy colocalization studies revealed that **3** and parent **3’** efficiently enter A549 cells within less than 90 min of incubation, both dyes accumulating mostly in cytoplasmic organelles. In previous studies, we found that **3’** localizes to some extent at mitochondria of live cells^[17]^ and, in this more detailed study, we found that intracellular distribution suggests colocalization of **3’** and **3** with low correlation to mitochondria, endoplasmic reticulum, and lysosomes (Figure 13).

**Figure 14:**
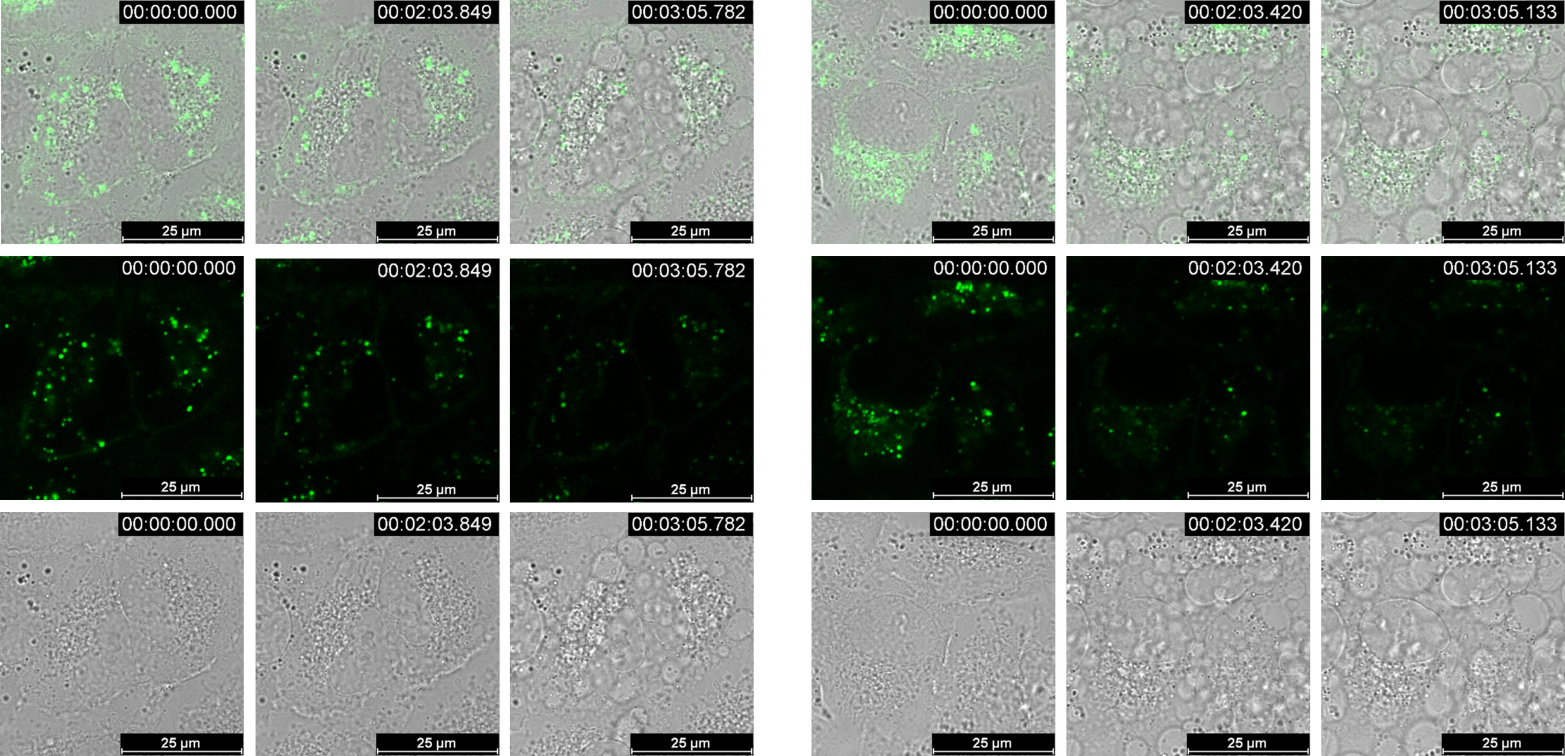
Confocal time‐lapse imaging of A549 cells treated with **3** (left) or **3’** (right) and irradiated at *λ*_exc_=457 nm by maximum power of the laser (Leica TCS SP8X, 50 mW), monitored at bright field and fluorescence during 3 min (real time movie in the Supporting Information).

Cells containing **3** and **3’** irradiated at 457 nm at the full power of the laser exhibited significant changes in the cell morphology within 1–3 min (cell blebbing, inner and outer membrane disintegration, cell contraction; Figure 14), suggesting strong cellular damage, while at the same conditions non‐treated cells remained inert. Such light induced changes strongly support very efficient ROS species production. In all photophysical studies measured open to the air, we found no signs of decomposition for the two compounds. Intriguingly, some bleaching of dyes emission was observed, suggesting that dye molecules are not disrupted with the photo‐induced process, but more likely act as catalysts.

## Conclusion

Three novel EDOT‐linked, water‐soluble tetracationic bis‐triarylborane chromophores **1**, **2**, and **3** and their neutral precursors **1N**, **2N**, and **3N** are reported. A significant red shift of absorption and emission is observed for these compounds compared to their thiophene‐containing analogues. Compound **3** exhibits the most bathochromically shifted emission of all of our water‐soluble A‐π‐A chromophores prepared to date which is still observable well into the NIR region. Even though a small increase in electron density at the boron atoms was found for the three EDOT‐containing compounds compared to their thiophene‐containing analogues, increased water‐stability was not achieved, and only the EDOT‐linked trixylylborane tetracation was stable in aqueous solution, indicating that direct attachment of a thiophene or even 3‐methylthiophene to the boron atom is insufficient to provide hydrolytic stability in aqueous solution. Thus, only EDOT‐derivative **3** and its thiophene analogue **3’** were further investigated in detail. Transient absorption spectroscopy revealed long‐lived (ca. 100 μs) excited states of **3** and **3’**, which were completely quenched by oxygen. For compound **3’**, the characteristic luminescence of singlet oxygen was observed and a quantum yield for singlet oxygen formation of 0.6 was determined. Overlap of the NIR emission of **3** with the emission from ^1^O_2_ hampered quantitative detection of the singlet oxygen emission.

Compounds **3** and **3’** bind within the minor groove of ds‐DNA and in BSA under physiological conditions, reporting the interaction by a strong fluorescence increase. However, only smaller, thiophene‐linked **3’** binds within the major groove of ds‐RNA, which is sterically too narrow for efficient binding of **3**, due to the steric demand of the EDOT bridge. Consequently, **3** can be considered to be a ds‐DNA specific fluorimetric probe (with respect to any RNA in the sample). Thus, the bulkiness of the linker between the two triarylborane units can control the selectivity of such tetracations directed toward conveniently sized polynucleotide grooves (B‐DNA) with respect to narrow grooved polynucleotides (e. g., A‐DNA minor groove or ds‐RNA major groove, Supporting Information, Table S1). However, the fluorescence selectivity between ds‐DNA and BSA, previously demonstrated for **3’**,^[30]^ was not found for compound **3**.

Furthermore, studies on A549 cells with **3** and **3’** show very efficient cellular uptake and accumulation in various cytoplasmic organelles with negligible toxicity even at high concentrations. However, under irradiation with strong visible light in a photoreactor (400–700 nm) or with a high‐power laser on a confocal microscope (at 457 nm), they cause severe cellular damage and death within several minutes.

Thus, both compounds can be considered to be intriguing theranostic agents, allowing their monitoring in vitro and likely in vivo by strong fluorescence and triggering their bioactivity by well‐focused visible light.

### Crystal structures

Deposition Number 2164833 (for **2N**) contains the supplementary crystallographic data for this paper. These data are provided free of charge by the joint Cambridge Crystallographic Data Centre and Fachinformationszentrum Karlsruhe Access Structures service.

## Conflict of interest

The authors declare no conflict of interest.

1

## Supporting information

As a service to our authors and readers, this journal provides supporting information supplied by the authors. Such materials are peer reviewed and may be re‐organized for online delivery, but are not copy‐edited or typeset. Technical support issues arising from supporting information (other than missing files) should be addressed to the authors.

Supporting Information

Supporting Information

## Data Availability

The data that support the findings of this study are available in the supplementary material of this article.
