# Supplementary material for: Electron‐Rich EDOT Linkers in Tetracationic bis‐Triarylborane Chromophores: Influence on Water Stability, Biomacromolecule Sensing, and Photoinduced Cytotoxicity
Source: Chemistry. 2022 Jul 4;28(48):e202201130. doi: 10.1002/chem.202201130 (PMC9543662; doi:10.1002/chem.202201130)
Supplement: Supplementary file 1 — Supporting Information [file CHEM-28-0-s001.pdf]

Chemistry–A European Journal

Supporting Information

Electron-Rich EDOT Linkers in Tetracationic bis-Triarylborane Chromophores: Influence on Water Stability, Biomacromolecule Sensing, and Photoinduced Cytotoxicity

Matthias Ferger, Chantal Roger, Eva Köster, Florian Rauch, Sabine Lorenzen, Ivo Krummenacher, Alexandra Friedrich, Marta Košćak, Davor Nestić, Holger Braunschweig, Christoph Lambert, Ivo Piantanida,* and Todd B. Marder*

Table of Contents

General Information.......... S2

Synthesis.......... S8

NMR Spectra.......... S15

Single-Crystal X-Ray Diffraction.......... S21

Linear Optical Properties.......... S23

Electrochemistry.......... S24

Transient Absorption.......... S30

Optical Properties in Sodium Cacodylate.......... S31

Study of Interactions with DNA, RNA, and Protein.......... S33

Theoretical Studies.......... S36

References.......... S63

General Information

Synthesis and Routine Characterization. Unless otherwise noted, the following conditions apply. Reactions were performed using standard Schlenk or glovebox (Innovative Technology Inc.) techniques under an atmosphere of argon. Only oven-dried glassware was used. Solvents used for reactions were HPLC grade, dried using an Innovative Technology Inc. Solvent Purification System, and further deoxygenated by saturation of the solvent with argon.

Bis[4-(*N*,*N*-dimethylamino)-2,6-dimethylphenyl]fluoroborane,[1] 2,2´-*bis*(3,4-ethylenedioxythiophene),[2] *bis*[4-(*N*,*N*-dimethylamino)-2,6-dimethylphenyl]-3-methyl-5-pinacolborylthiophene-2-ylborane,[3] *bis*[4-(*N*,*N*-dimethylamino)-2,6-dimethylphenyl]-2,6-dimethyl-4-pinacolboryl-phenylborane,[4] $Pd_2(dba)_3{\cdot}CHCl_3$ (dba = dibenzylideneacetone)[5] and $[Ir(COD)(\mu\text{-}OMe)]_2$ (COD = 1,5-cyclooctadiene)[6] were synthesized according to literature procedures. All other starting materials were purchased from commercial sources and were used without further purification.

Reaction progress was monitored using thin layer chromatography (TLC) plates pre-coated with a layer of silica (Polygram® Sil G/UV254) with fluorescent indicator UV254 from Marchery-Nagel. Automated flash column chromatography was performed using a Biotage® Isolera Four system with silica gel (Biotage SNAP cartridge KP-Sil 50g or KP-Sil 100g obtained from Biotage) as the stationary phase and the solvent system indicated. Solvents were generally removed *in vacuo* using a rotary evaporator at a maximum temperature of 50 °C.

^{1}H, $^{13}C\{^{1}H\}$ and $^{11}B\{^{1}H\}$ solution NMR spectroscopic data were obtained at ambient temperature using a Bruker Avance 300 III (operating at 300 MHz for ^{1}H, 75 MHz for $^{13}C\{^{1}H\}$ and 96 MHz for $^{11}B\{^{1}H\}$), or a Bruker Avance 500 NMR spectrometer (operating at 500 MHz for ^{1}H, 125 MHz for $^{13}C\{^{1}H\}$ and 160 MHz for $^{11}B\{^{1}H\}$). Chemical shifts (δ) were referenced to solvent peaks as follows. ^{1}H NMR spectra were referenced via residual proton resonances of CD_2Cl_2 (5.32 ppm), $CDCl_3$ (7.26 ppm) and CD_3OD (3.31 ppm). $^{13}C\{^{1}H\}$ spectra were referenced to CD_2Cl_2 (53.84 ppm) and CD_3OD (49.00 ppm).

Elemental analyses were performed on an Elementar vario MICRO cube elemental analyzer. As is common for related organo-$B(Aryl)_2$ compounds, carbon analyses of our compounds were up to 2.3% below the calculated value, while hydrogen, nitrogen and sulfur analyses were satisfactory. This has been ascribed previously to the formation of boron carbide.[7] High resolution mass spectrometry (HRMS) was performed with a Thermo Fisher Scientific Exactive Plus Orbitrap MS System. ESI measurements were performed with a HESI source at 50 °C. APCI measurements were performed with an ACPI source and Corona needle at 400 °C, unless otherwise noted.

Single-Crystal X-Ray Diffraction. Crystals suitable for single-crystal X-ray diffraction were selected, coated in perfluoropolyether oil, and mounted on MiTeGen sample holders. Diffraction data of **2N** were collected on a Rigaku Oxford Diffraction XtaLAB Synergy diffractometer with a semiconductor HPA-detector (HyPix-6000) and multi-layer mirror monochromated Cu-K_α radiation. The crystal was cooled using an Oxford Cryostream or Bruker Kryoflex low-temperature device. Data were collected at 100 K. The images were processed and corrected for Lorentz-polarization effects and absorption as implemented in the CrysAlis^{Pro} software. The structure was solved using the intrinsic phasing method (SHELXT)[8] and Fourier expansion technique. All non-hydrogen atoms were refined in anisotropic approximation, with hydrogen atoms 'riding' in idealized positions, by full-matrix least squares against F^2 of all data, using SHELXL[9] software and the SHELXLE graphical user interface.[10]

Reflections were slightly smeared along the c* direction, which correspond to the longest c axis (ca. 46 Å) in direct space. Hence, the reflections were not always well resolved along this axis and reflection overlap may lead to wrong intensities on integration. As a result, a few reflections with too high intensities had to be omitted from the refinement.

We found one disordered hexane solvent molecule. The remaining residual density was fitted by an ethyl acetate molecule of low occupancy disordered via inversion symmetry as the best model. Several restraints were applied to the anisotropic displacement parameters (SIMU and ISOR) and 1,2- and 1,3-distances (DFIX and SADI) of the disordered solvent molecules.

Diamond[11] software was used for graphical representation. Crystal data and experimental details are listed in Table S1; full structural information has been deposited with Cambridge Crystallographic Data Centre. CCDC-2164833 (**2N**).

Linear Optical Properties. All measurements were performed in standard quartz cuvettes (1 cm x 1 cm cross-section) under ambient conditions, unless stated otherwise. UV-visible absorption spectra were recorded using an Agilent 8453 diode array UV-visible spectrophotometer. The molar extinction coefficients were calculated from three independently prepared samples in toluene (**1N**-**3N**) and MeCN (**1**, **3**) solutions. Emission spectra were recorded using an Edinburgh Instruments FLSP920 spectrometer equipped with a 450 W Xenon arc lamp, double monochromator for both excitation and emission, and a red-sensitive photomultiplier (PMT-R928P) and a near-IR PMT as detectors, operating in right-angle geometry mode, and all spectra were fully corrected for the spectral response of the instrument. All solutions used in photophysical measurements had concentrations lower than 5×10^{-6} M to minimize inner filter effects during fluorescence measurements. The **fluorescence quantum yields** were measured using a calibrated integrating sphere (inner diameter: 150 mm) from Edinburgh Instruments combined with the FLSP920 spectrometer

described above. For solution-state measurements, the longest-wavelength absorption maximum of the compound in the respective solvent was chosen as the excitation wavelength, unless stated otherwise. **Fluorescence lifetimes** were recorded using the time-correlated single-photon counting (TCSPC) method using an Edinburgh Instruments FLS980 spectrometer equipped with a high speed photomultiplier tube positioned after a single emission monochromator. Measurements were made in right-angle geometry mode, and the emission was collected through a polarizer set to the magic angle. Solutions were excited with a pulsed diode laser at a wavelength of 472.6 nm at repetition rates of 10 or 20 MHz, as appropriate. The full-width-at-half-maximum (FWHM) of the pulse from the diode laser was ca. 80 ps with an instrument response function (IRF) of ca. 230 ps FWHM and ca. 200 ps with an instrument response function (IRF) of ca. 1120 ps FWHM, respectively. The IRFs were measured from the scatter of an aqueous suspension of Ludox at the excitation wavelength. Decays were recorded to 10 000 counts in the peak channel with a record length of 8 192 channels. The band pass of the emission monochromator and a variable neutral density filter on the excitation side were adjusted to give a signal count rate of <60 kHz. Iterative reconvolution of the IRF with one decay function and non-linear least-squares analysis were used to analyze the data. The quality of all decay fits was judged to be satisfactory, based on the calculated values of the reduced χ^2 and Durbin-Watson parameters and visual inspection of the weighted residuals.

Electrochemical Measurements. All cyclic voltammetry experiments were conducted in an argon-filled glovebox using a Gamry Instruments Reference 600 potentiostat. A standard three-electrode cell-configuration was employed using a platinum disk working electrode, a platinum wire counter electrode, and a silver wire reference electrode separated by a Vycor frit, serving as the reference electrode. The redox potentials are referenced to the ferrocene/ferrocenium ([Fc/Fc^+]) redox couple by using decamethylferrocene as an internal standard. Tetra-*n*-butylammonium hexafluorophosphate ([$n\mathrm{Bu}_4$N][PF_6]) was employed as the supporting electrolyte. Compensation for resistive losses (*iR* drop) was employed for all measurements.

Transient Absorption Measurements. Transient absorption spectra were measured with an Edinburgh LP920 laser flash spectrometer equipped with a EKSPLA NT340 Nd:YAG laser with integrated optical parametric oscillator, a 450 W Xe arc flash lamp, a Hamamatsu R955 photomultiplier and a Tektronix TD3012B oscilloscope for detection of the spectra. The pump and white light beams were perpendicular to each other. The transient maps were obtained by measuring temporal decay profiles in 4 nm steps between *ca.* 33,333 cm^{-1} (300 nm) and 12,500 cm^{-1} (800 nm) and corrected for fluorescence. The instrument response (*ca.* 8 ns) of the set-up was determined by measuring the scattered light using a LUDOX AS-30 colloidal

silica suspension in water. Decay curves were fitted with the tailfit function of the spectrometer software. The quality of all decay fits was judged to be satisfactory, based on the calculated values of the reduced χ^2 and Durbin-Watson parameters and visual inspection of the weighted and autocorrelated residuals. All solvents were spectroscopic grade and were used without further purification. The samples were dissolved in MeCN, degassed by at least 5 freeze-pump-thaw cycles, and placed in 10 x 10 mm quartz-cuvettes equipped with a *Young´s* valve. The samples were excited with *ca.* ~5 ns laser pulses at 10 Hz repetition rate. Measurements were performed at pulse energies of 1.2 mJ (excitation at 460 and 550 nm). The stability of the samples was verified by recording the steady-state absorption spectra before and after the time-resolved measurements.

Optical Properties in Sodium Cacodylate. UV-visible absorption spectra were recorded on a Varian Cary 100 Bio spectrometer; excitation and emission spectra were recorded on a Varian Cary Eclipse fluorimeter.

Study of Interactions with DNA, RNA, and BSA. Polynucleotides were purchased as noted: poly A – poly U, *calf thymus* (ct)-DNA (Aldrich) and dissolved in sodium cacodylate buffer, *I* = 0.05 M, pH = 7.0. The ct-DNA was additionally sonicated and filtered through a 0.45 mm filter to obtain mostly short (ca. 100 base pairs) rod-like B-helical DNA fragments.[12] The polynucleotide concentration was determined spectroscopically[13] as the concentration of phosphates (corresponds to *c*(nucleobase)). Bovine Serum Albumin (BSA) (Sigma-Aldrich) was dissolved in sodium cacodylate buffer, *I* = 0.05 M, pH = 7.0 and its concentration determined spectroscopically using a NanoDrop spectrophotometer at 280 nm using its molar extinction coefficient 43,824 $M^{-1}cm^{-1}$. **Fluorimetric titrations** were performed by adding portions of polynucleotide or BSA solution into the solution of the studied compound being studied ($c = 5 \times 10^{-8}$ M). In fluorimetric experiments, an excitation wavelength of λ_{exc} = 471 nm was used to avoid absorption of excitation light by added polynucleotides or BSA. After mixing polynucleotides or BSA with the compound, equilibrium was reached in less than 120 s. Fluorescence spectra were collected using an excess of DNA/RNA ($r_{[\mathbf{3}]/[DNA]}$ <0.3) to assure one dominant binding mode. To obtain binding constants (*Ks*), titration data were processed by means of non-linear fitting to the Scatchard equation (McGhee, von Hippel formalism),[14] which gave values of the ratio of [bound compound] / [polynucleotide] in the range 0.1–0.3, but for easier comparison, all *Ks* values were re-calculated for the fixed n = 0.25 (for ds-DNA). Calculated values of *Ks* have satisfactory correlation coefficients (>0.99). Titration data with BSA gave an excellent correlation (>0.999) to non-linear regression fitting to a 1:1 (**3**:BSA) stoichiometry model, giving a value of *Ks*. **Circular dichroism (CD) spectra** were recorded on a JASCO J-815 spectropolarimeter at room temperature using 1 cm path quartz cuvettes with a scanning speed of 200 nm/min (an average of 3 accumulations). A buffer background

was subtracted from each spectrum. CD experiments were performed by adding portions of compound stock solution into the solution of the polynucleotide (c = 2×10^{-5} M). **Thermal melting experiments** were performed on a Varian Cary 100 Bio spectrometer in quartz cuvettes (1 cm). The measurements were carried out in aqueous buffer solution at pH 7.0 (sodium cacodylate buffer, I = 0.05 M). Thermal melting curves for ds-DNA, ds-RNA and their complexes with **3** were determined by monitoring the absorption change at 260 nm as a function of temperature.[15] *Tm* values are the midpoints of the transition curves determined from the maximum of the first derivative and checked graphically by the tangent method. The ΔTm values were calculated by subtracting *Tm* of the free nucleic acid from *Tm* of the complex. Every ΔTm value here reported was the average of at least two measurements. The error in ΔTm is $\pm$ 0.5 °C.

Theoretical studies. All calculations (DFT and TD-DFT) were carried out with the Gaussian 16 (16.A.03)[16] program package and were performed on a parallel cluster system. GaussView (6.0.16), Avogadro (1.2.0)[17] and multiwfn[18] were used to visualize the results, to measure calculated structural parameters, and to plot orbital surfaces (isovalue: $\pm$ 0.030 $[e\ a_0^{-3}]^{1/2}$). The ground-state geometries were optimized using the B3LYP functional[19] in combination with the 6-31G+(d,p) basis set.[20-21] An ultrafine integration grid and no symmetry constraints were used for all molecules. Frequency calculations were performed on the optimized structures to confirm them to be local minima showing no negative (imaginary) frequencies. Based on these optimized structures, the lowest-energy vertical transitions (gas-phase) were calculated (singlets, 25 states) by TD-DFT, using the CAM-B3LYP functional in combination with the 6-31G+(d,p) basis set.[20-21] Solvent effects were included using the PCM model as implemented in Gaussian.

Cells. Human lung carcinoma (A549; ATCC CCL-185) cells were obtained from the ATCC Cell Biology Collection and were cultured according to the manufacturer's instructions. Cells were grown in Dulbecco Modified Eagle's Medium (DMEM, Sigma Aldrich, USA) supplemented with 10% of fetal bovine serum (FBS, Sigma Aldrich, USA) at 37 °C and 5% CO_2 in a humidified atmosphere.

MTT assay. The cell survival to compounds **3** and **3'** was determined using MTT assay. Stock solutions of compounds **3** and **3'** were prepared in DMSO, while dilutions were prepared in DMEM (10% FBS). Cells were seeded in 96-well tissue culture plates (7×10^3 cells/well), and 24 h later treated with compounds **3** or **3'** (concentration range of 0.01 µM - 10 µM). Cells treated with the same dilutions of DMSO represented control sample. For non-irradiated sample (no VIS), cells were incubated for 72 h (37 °C, 5% CO_2) after treatment. Cells were irradiated in a Luzchem reactor with visible light range 90 min after treatment (VIS, 400-700 nm, 8 lamps, in total 56 W, Dose 50.6 mw*m^{-2}) ~ 18 cm lamp to cell-plate, for 10, 30, or 60

min, and then left in the incubator overnight (37 °C, 5% CO_2). Irradiation was performed for three days in a row at the same time point each day. Medium was removed from all samples 72 h after treatment, and 1X MTT solution was added into each well. Plates were incubated (37 °C, 5% CO_2) for 4 h allowing formazan crystals to form. The resulting MTT-formazan products were dissolved using DMSO, and their absorbance was measured using a microplate reader (Awareness Technology, Inc., United States) at 600 nm. Results are presented as cell survival representing absorbance mean ± SD made in four replicates, relative to the mean absorbance of the control sample (DMSO).

Confocal microscopy. Live imaging of the cells treated with compounds **3** and **3'** was performed on A549 cell line. Cells (1 × 10^5 cells/well) were seeded in Ibidi imaging cell chambers (Ibidi, Germany), and 24 h later treated with 10 µM solution of compounds **3** or **3'**. After incubation (37 !C, 5% CO_2) for 90 min, cells were rinsed with DMEM (10% FBS) and incubated (37 °C, 5% CO_2) with 100 nM MitoTracker Deep Red solution (Invitrogen, Molecular Probes) for 10 min, 50 nM Lysotracker Deep Red solution (Invitrogen, Molecular Probes) for 30 min or 1 µM ER-Tracker Red solution (Invitrogen, Molecular Probes) for 15 min. After incubation, medium was replaced with fresh DMEM (10% FBS) and cells were immediately observed by Leica SP8 X confocal microscope (Leica Microsystems, Germany). Co-localization is assessed by Pearson's correlation coefficient. Analysis was done by ImageJ (NIH, USA) software and appropriate JACoP plugin.[22]

Synthesis

5,5´-Dibromo-2,2´-*bis*(3,4-ethylenedioxythiophene)

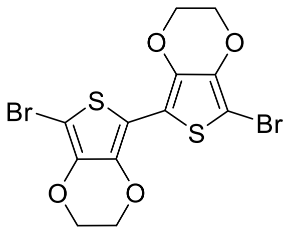

With the exclusion of light, 2,2´-*bis*(3,4-ethylenedioxythiophene) (555 mg, 1.97 mmol, 1.0 eq) was dissolved in CH_2Cl_2 (150 mL) and cooled to -15 °C. *N*-bromosuccinimide (770 mg, 4.32 mmol, 2.20 eq) was added in small portions within 45 min and the reaction was stirred for another 1 h at -15 °C. The reaction mixture was poured into a mixture of ice water / 25% aqueous NH_3 (500 mL / 12 mL) and extracted with CH_2Cl_2 (4 × 100 mL). Residual solid was removed from the organic phases by filtration and the combined organic phase was washed with water (2 x 100 mL) and brine (100 mL). The solvent was removed *in vacuo* at a maximum temperature of 30 °C. The product 5,5´-dibromo-2,2´-*bis*(3,4-ethylenedioxythiophene) was obtained as an off-white solid (788 mg, 91%) and stored at 8 °C. The spectroscopic data match those reported previously.[23]

^{1}H NMR (300 MHz, $CDCl_3$): δ = 4.31 (m, 8H) ppm.

5,5'-Bis[*bis*(4-(*N*,*N*-dimethylamino)-2,6-dimethylphenyl)boryl]-2,2'-bis(3,4-ethylenedioxythiophene) (1N)

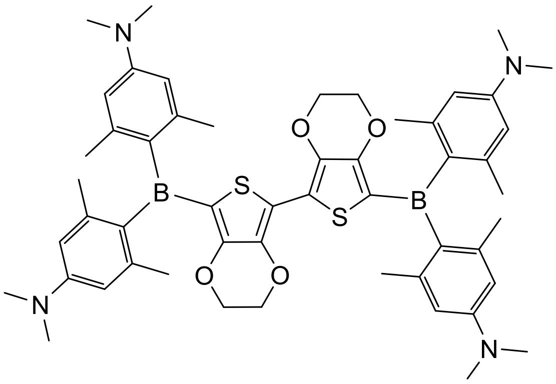

The compound 2,2´-*bis*(3,4-ethylenedioxythiophene) (200 mg, 703 μmol, 1 eq) was dissolved in THF (6 mL), cooled to -78 °C and treated dropwise with *n*-butyllithium (2.5 mol in hexane, 620 μL, 2.2 eq). The solution was stirred for 45 min at -78 °C. *Bis*[4-(*N*,*N*-dimethylamino)-2,6-dimethylphenyl]fluoroborane (460 mg, 1.41 mmol, 2.0 eq) was dissolved in THF (10 mL) and added to the reaction mixture. The mixture was warmed to r.t. and stirred for 3 d, until the starting material was consumed according to TLC (EtOAc /hexane 1:4). Water (5 mL) was added and the product was extracted with CH_2Cl_2. After removing the solvent *in vacuo*, the solid was purified by automated flash column chromatography (hex/EtOAc 4:1), giving compound **1N** as an orange solid (173 mg, 27%).

^{1}H NMR (500 MHz, CD_2Cl_2): δ = 6.35 (s, 8H), 4.21 (m, 8H), 2.95 (s, 24H), 2.09 (s, 24H) ppm.

^{13}C{^{1}H} NMR (125 MHz, CD_2Cl_2): δ = 151.3, 147.8, 142.9, 139.8, 133.5, 125.4, 121.9, 111.6, 65.2, 65.2, 40.3, 23.8 ppm.

HRMS (APCI) m/z: $[M+H]^+$ found: 895.4624; calc. for $[C_{52}H_{65}B_2N_4O_4S_2]^+$: 895.4589 (|Δ| = 3.91 ppm).

Elemental analysis Calc. (%) for $C_{52}H_{64}B_2N_4O_4S_2$: C 69.80, H 7.21, N 6.26, S 7.17; found: C 69.69, H 7.41, N 6.26, S 6.85.

5,5'-Bis[4-(*bis*(4-(*N*,*N*-dimethylamino)-2,6-dimethylphenyl)boryl)-4-methylthiophen-2-yl]-2,2'-bis(3,4-ethylenedioxythiophene) (2N)

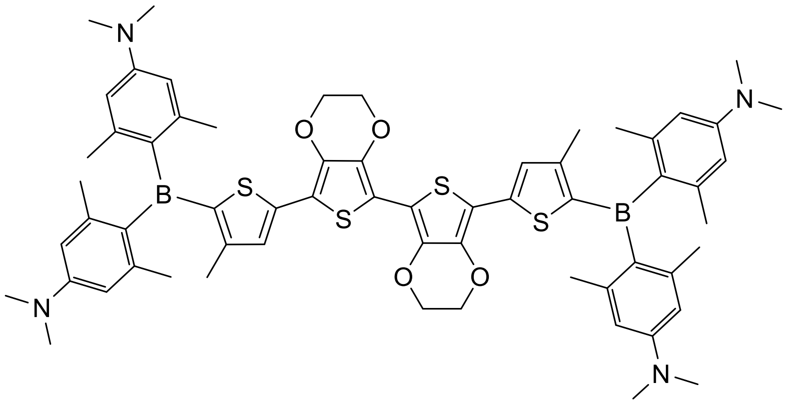

Bis[4-(*N*,*N*-dimethylamino)-2,6-dimethylphenyl]-3-methyl-5-pinacolborylthiophene-2-ylborane (133 mg, 251 *μ*mol, 2.0 eq), 5,5´-dibromo-2,2´-*bis*(3,4-ethylenedioxythiophene) (55.3 mg, 125 *μ*mol, 1.0 eq), SPhos (7.2 mg, 17.6 *μ*mol, 14 mol%), $Pd_2(dba)_3{\cdot}CHCl_3$ (9.1 mg, 8.8 *μ*mol, 7 mol%) and Cs_2CO_3 (246 mg, 754 μmol, 6.0 eq) were dissolved in a mixture of toluene (3 mL) and water (1.5 mL). The mixture was stirred for 2 d at 85 °C, until the starting material was consumed according to TLC (EtOAc /hexane 1:2). The organic phase was separated and the aqueous phase was extracted with CH_2Cl_2 (4 × 10 mL). After removing the solvent *in vacuo* from the combined organic phase, the solid was purified by automated flash column chromatography (hex/EtOAc 4:1). For further purification, the solid was recrystallized from CH_2Cl_2/MeOH, giving **2N** as a red solid (20 mg, 15%).

^{1}H NMR (300 MHz, CD_2Cl_2): δ = 7.22 (m, 2H), 6.37 (s, 8H), 4.37 (s, 8H), 2.96 (s, 24H), 2.08 (s, 24H), 2.00 (s, 6H) ppm.

^{13}C{^{1}H} NMR (125 MHz, CD_2Cl_2): δ = 151.4, 148.4, 145.6, 143.6, 142.6, 138.4, 137.9, 134.3, 128.6, 111.7, 111.2, 108.8, 65.6, 65.5, 40.3, 23.7, 16.2 ppm.

HRMS (APCI) m/z: $[M+H]^+$ found: 1087.4684; calc. for $[C_{62}H_{73}B_2N_4O_4S_4]^+$: 1087.4695 (|Δ| = 1.01 ppm).

Elemental analysis Calc. (%) for $C_{62}H_{72}B_2N_4O_4S_4$: C 68.50, H 6.68, N 5.15, S 11.80; found: C 66.36, H 7.11, N 5.01, S 11.40.

5,5'-Bis[4-(*bis*(4-(*N*,*N*-dimethylamino)-2,6-dimethylphenyl)boryl)-3,5-dimethylphenyl]-2,2'-*bis*(3,4-ethylenedioxythiophene) (3N)

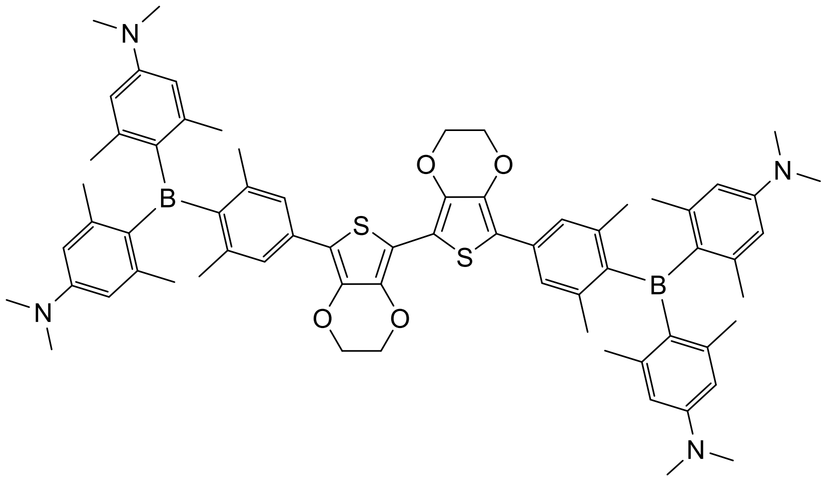

Bis[4-(*N*,*N*-dimethylamino)-2,6-dimethylphenyl]-2,6-dimethyl-4-pinacolboryl-phenylborane (375 mg, 697 *µ*mol, 2.0 eq), 5,5´-dibromo-2,2´-*bis*(3,4-ethylenedioxythiophene) (153 mg, 349 *µ*mol, 1.0 eq), SPhos (20 mg, 48.9 *µ*mol, 14 mol%), $Pd_2(dba)_3·CHCl_3$ (25 mg, 24.4 *µ*mol, 7 mol%) and Cs_2CO_3 (682 mg, 2.09 mmol, 6.0 eq) were dissolved in a mixture of toluene (8 mL) and water (4 mL). The mixture was stirred for 2 d at 85 °C, until the starting material was consumed according to TLC (EtOAc /hexane 1:2). Water (15 mL) was added and the product was extracted with CH_2Cl_2. After removing the solvent *in vacuo* from the combined organic phase, the solid was purified by automated flash column chromatography (hex/EtOAc 4:1). For further purification, the solid was recrystallized from CH_2Cl_2/MeOH, giving compound **3N** as a yellow solid (74 mg, 19%).

^{1}H NMR (500 MHz, CD_2Cl_2): δ = 7.31 (s, 4H), 6.31 (m, 8H), 4.39 (m, 8H), 2.95 (s, 24H), 2.05 (s, 12H), 2.01 (s, 12H), 1.97 (s, 12H) ppm.

^{13}C{^{1}H} NMR (125 MHz, CD_2Cl_2): δ = 151.7, 148.5, 143.2, 142.9, 141.0, 138.5, 138.1, 136.3, 133.3, 124.7, 115.9, 111.9, 111. 8, 108.4, 65.4, 65.2, 40.2, 24.0, 23.8, 23.1 ppm.

HRMS (APCI) m/z: $[M+H]^+$ found: 1103.5890; calc. for: $[C_{68}H_{81}B_2N_4O_4S_2]^+$: 1103.5841 (|Δ| = 4.44 ppm).

Elemental analysis Calc. (%) for $C_{68}H_{80}B_2N_4O_4S_2$: C 74.04, H 7.31, N 5.08, S 5.81; found: C 73.51, H 7.49, N 5.06, S 5.44.

5,5'-Bis[*bis*(4-(*N*,*N*,*N*-trimethylammonium)-2,6-dimethylphenyl)boryl]-2,2'-bis(3,4-ethylenedioxythiophene) tetratriflate (1)

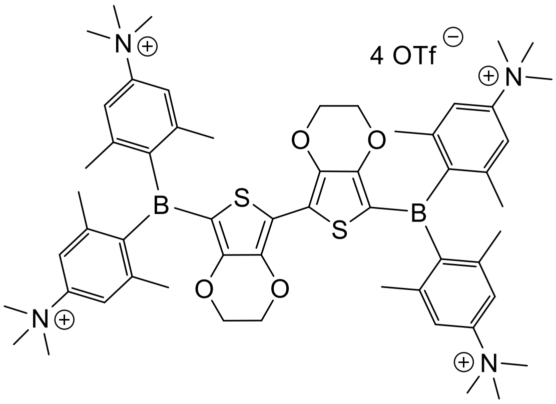

Compound **1N** (14.7 mg, 16.4 *μ*mol, 1.0 eq) was dissolved in CH_2Cl_2 (2 mL). Methyl triflate (9.3 *μ*L, 82.1 *μ*mol, 5 eq) was added and the reaction was stirred at r.t. for 1 d. The precipitate was collected by filtration and washed with CH_2Cl_2 (3 × 5 mL), giving compound **1** as a green-yellow solid (12.3 mg, 48%).

^{1}H NMR (500 MHz, CD_3OD): δ = 7.54 (s, 8H), 4.21 (m, 8H), 3.66 (s, 36H), 2.33 (s, 24H) ppm.

^{13}C{^{1}H} NMR (125 MHz, CD_3OD): 152.3, 148.8, 146.6, 144.9, 141.9, 126.4, 123.5, 121.8 (q, *J* = 318 Hz), 119.2, 66.3, 66.0, 57.5, 23.7 ppm.

HRMS (ESI pos) m/z: $[M-2OTf]^{2+}$ found: 626.2263; calc. for $[C_{58}H_{76}B_2N_4S_4F_6O_{10}]^{2+}$: 626.2262 (|Δ| = 0.16 ppm).

Elemental analysis Calc. (%) for $C_{60}H_{76}B_2F_{12}N_4O_{16}S_6$: C 46.46, H 4.94, N 3.61, S 12.40; found: C 44.19, H 5.13, N 3.43, S 11.73.

5,5'-Bis[4-(*bis*(4-(*N*,*N*,*N*-trimethylammonium)-2,6-dimethylphenyl)boryl)-4-methylthiophen-2-yl]-2,2'-bis(3,4-ethylenedioxythiophene) tetratriflate (2)

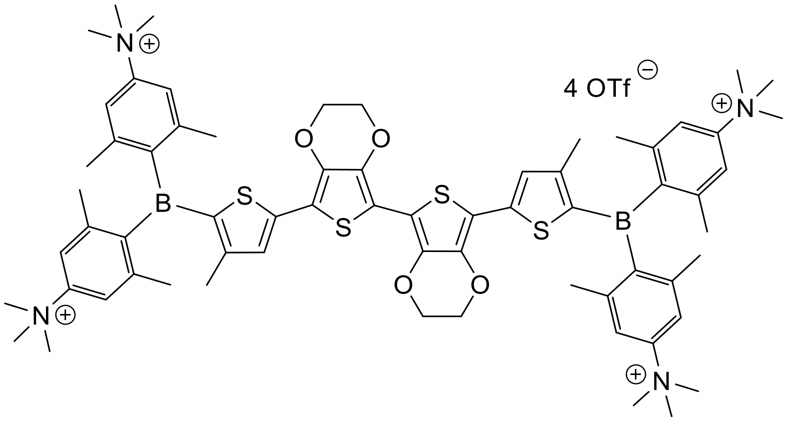

Compound **2N** (8.2 mg, 7.54 *µ*mol, 1.0 eq) was dissolved in CH_2Cl_2 (20 mL). Methyl triflate (27.3 *µ*L, 241 *µ*mol, 32 eq) was added and the reaction was stirred at r.t. for 1 d. The precipitate was collected by filtration and washed with CH_2Cl_2 (3 × 5 mL), giving 9.7 mg of compound **2**, contaminated with an unidentified impurity, as a violet solid.

Despite a prominent signal of an unidentified impurity at δ = 4.59 ppm in the ^{1}H NMR spectrum, a reasonable assignment of the remaining signals to the respective protons of compound **2** was possible. Together with an unambiguous identification of **2** *via* HRMS, we propose **2** as the main product of the described reaction. A broader discussion of this is given in the main text.

^{1}H NMR (300 MHz, CD_3OD): δ = 7.59 (m, 8H), 7.35 (s, 2H), 3.67 (m, 36H), 2.33 (m, 24H), 2.02 (s, 6H) ppm.

HRMS (ESI pos) m/z: $[M-2OTf]^{2+}$ found: 722.2300; calc. for $[C_{68}H_{84}B_2N_4S_6F_6O_{10}]^{2+}$: 722.2296 (|Δ| = 0.55 ppm).

5,5'-*Bis*[4-(*bis*(4-(*N*,*N*,*N*-trimethylammonium)-2,6-dimethylphenyl)boryl)-3,5-dimethylphenyl]-2,2'-*bis*(3,4-ethylenedioxythiophene) tetratriflate (3)

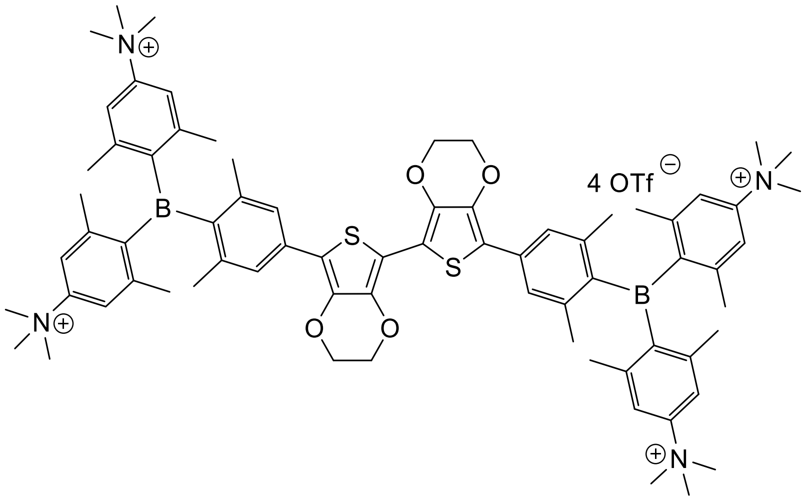

Compound **3N** (15.0 mg, 13.6 *μ*mol, 1.0 eq) was dissolved in CH_2Cl_2 (2 mL). Methyl triflate (6.9 *μ*L, 61.2 *μ*mol, 4.5 eq) was added and the reaction was stirred at r.t. for 1 d. The precipitate was collected by filtration and washed with CH_2Cl_2 (3 × 5 mL), giving compound **3** as a dark red solid (16 mg, 67%).

^{1}H NMR (500 MHz, CD_3OD,): δ = 7.57 (m, 8H), 7.42 (m, 4H), 4.42 (m, 8H), 3.66 (s, 36H), 2.25 (s, 12H), 2.15 (s, 12H), 2.04 (s, 12H) ppm.

^{13}C{^{1}H} NMR (125 MHz, CD_3OD): 149.7, 149.6, 145.0, 144.4, 142.7, 140.7, 139.4, 137.5, 126.2, 121.8 (q, *J* = 318 Hz), 120.1, 115.8, 110.5, 66.3, 66.2, 57.5, 23.6, 23.4 ppm.

HRMS (ESI pos) m/z: $[M\text{-}2OTf]^{2+}$ found: 730.2910; calc. for $[C_{74}H_{92}B_2N_4S_4F_6O_{10}]^{2+}$ 730.2888 (|Δ| = 3.01 ppm).

Elemental analysis Calc. (%) for $C_{76}H_{92}B_2F_{12}N_4O_{16}S_6$: C 51.88, H 5.27, N 3.18, S 10.93; found: C 50.31, H 5.58, N 2.94, S 10.33.

NMR Spectra

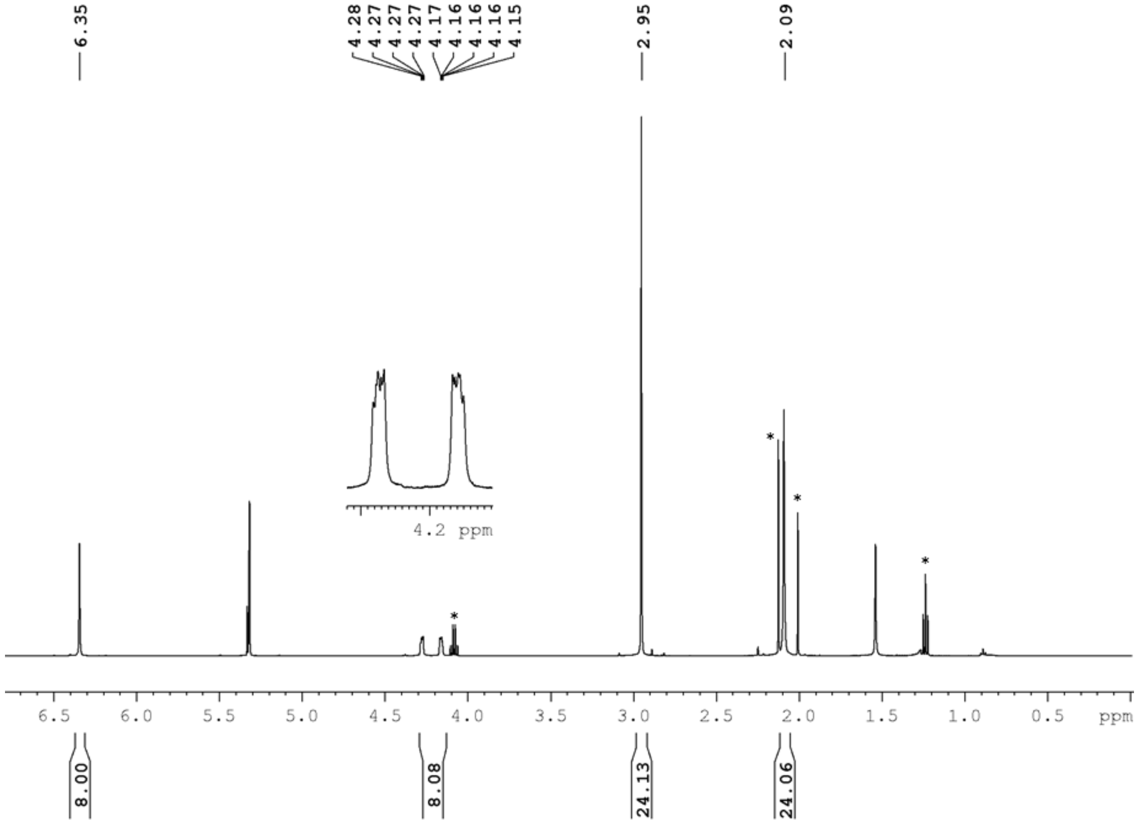

Figure S1. ^{1}H NMR spectrum of **1N** in CD_2Cl_2 at 500 MHz. Residual solvent peaks (EtOAc, acetone) are marked with *.

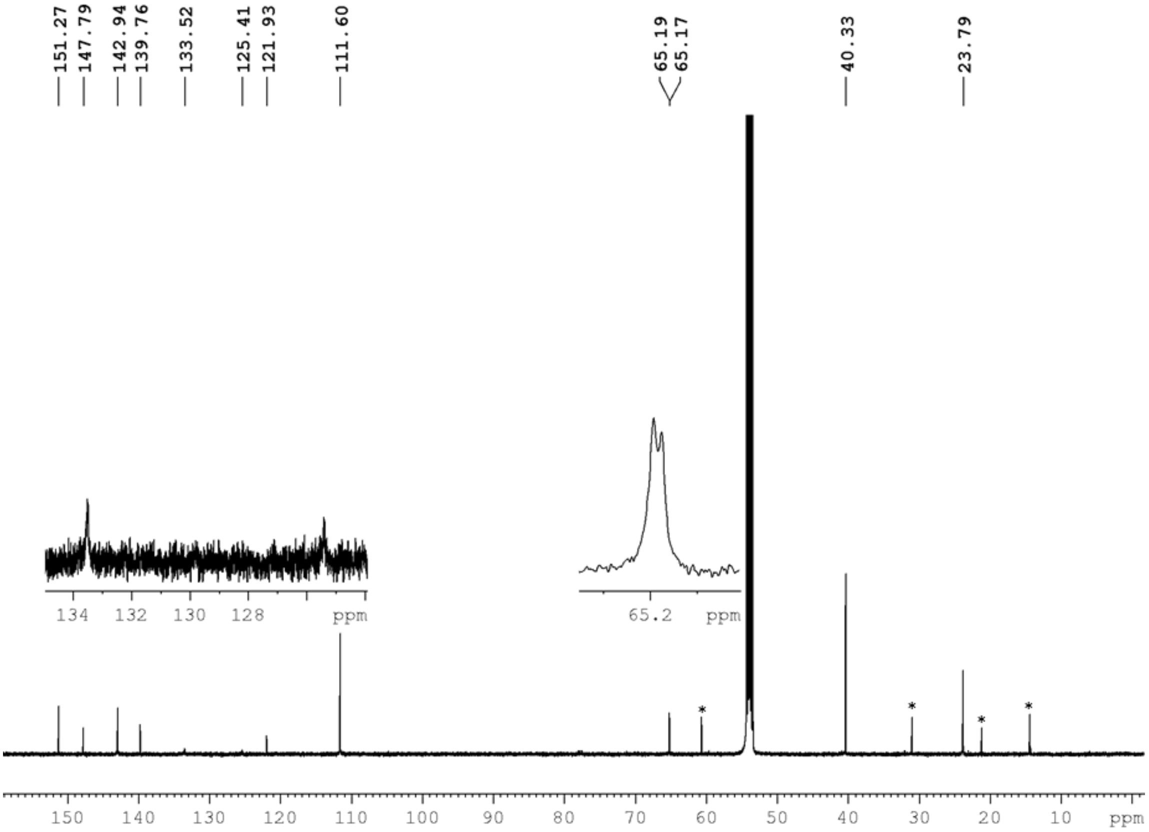

Figure S2. $^{13}C\{^{1}H\}$ NMR spectrum of **1N** in CD_2Cl_2 at 75 MHz. Residual solvent peaks (EtOAc, acetone) are marked with *.

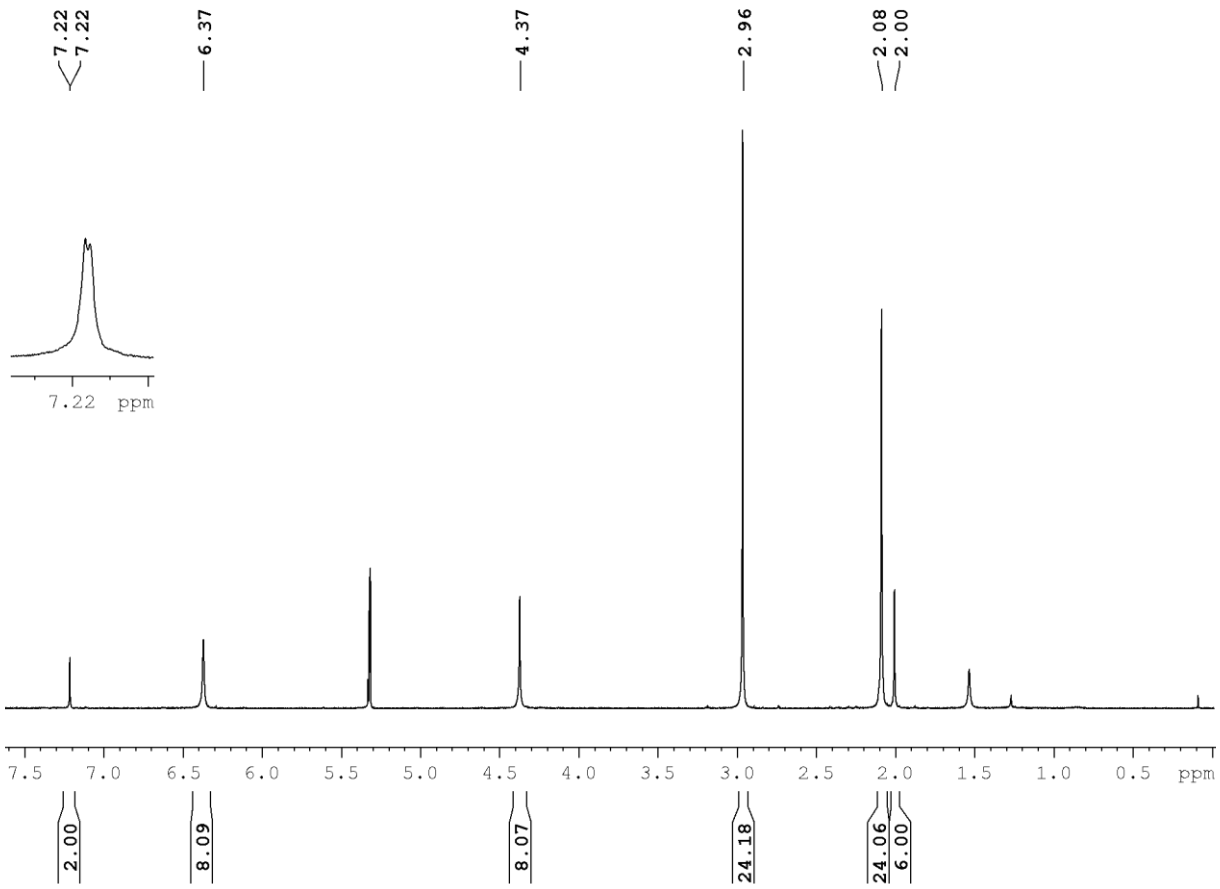

Figure S3. ^{1}H NMR spectrum of **2N** in CD_2Cl_2 at 300 MHz.

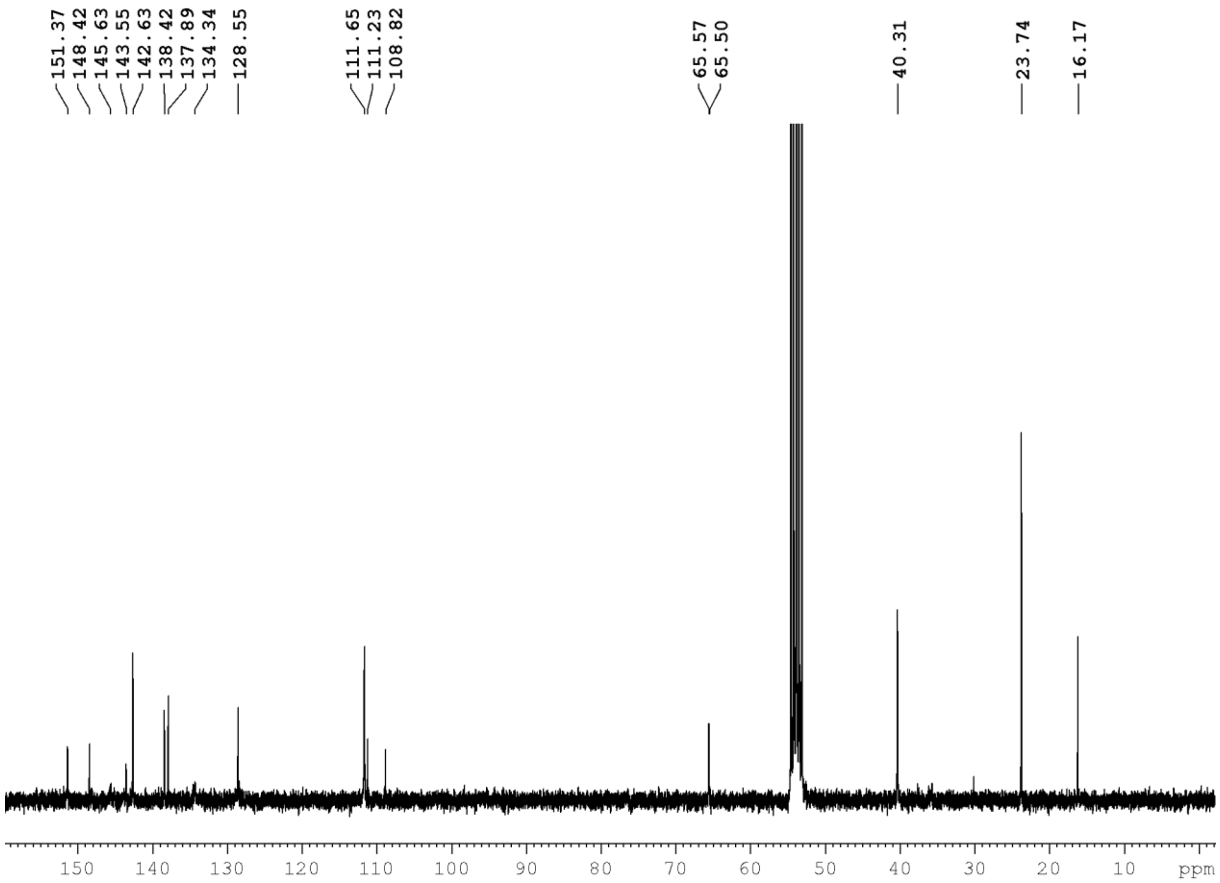

Figure S4. $^{13}C\{^{1}H\}$ NMR spectrum of **2N** in CD_2Cl_2 at 75 MHz.

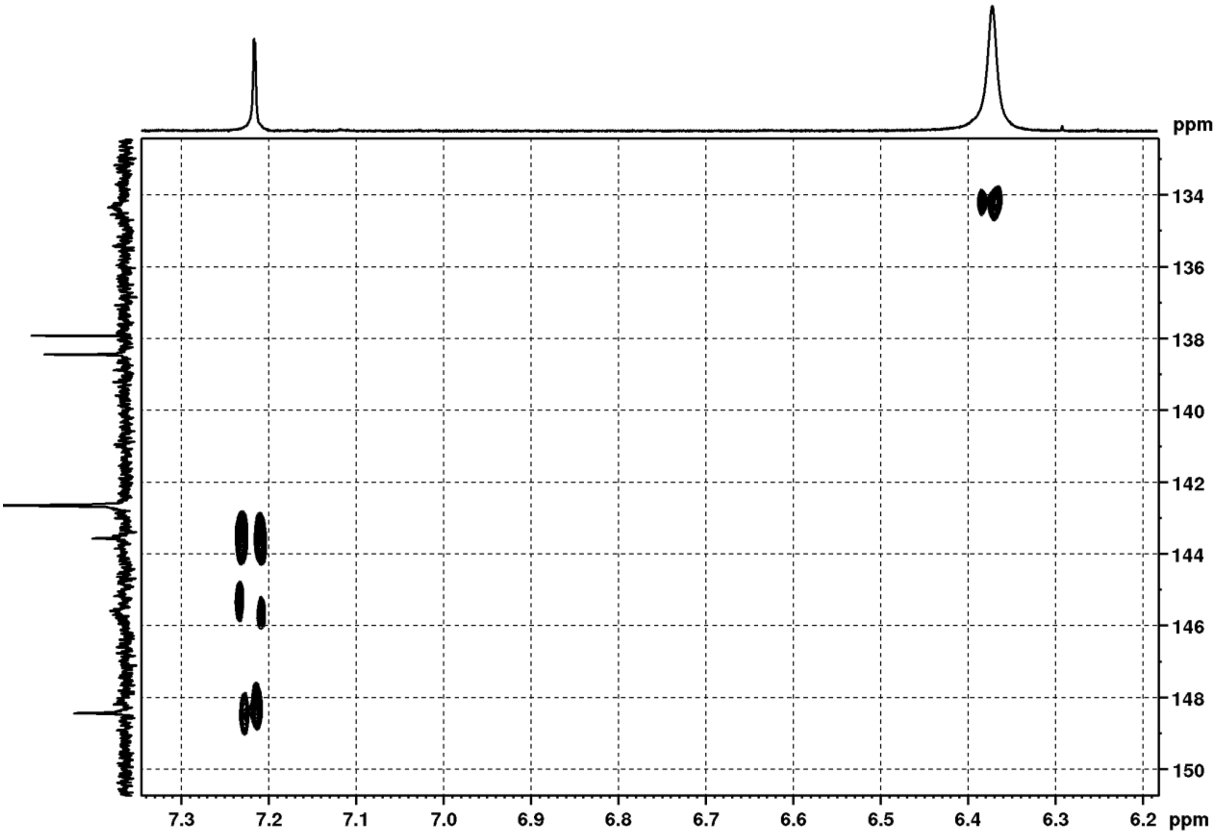

Figure S5. ^{13}C, ^{1}H HMBC NMR spectrum of **2N** in CD_2Cl_2 at 300 MHz.

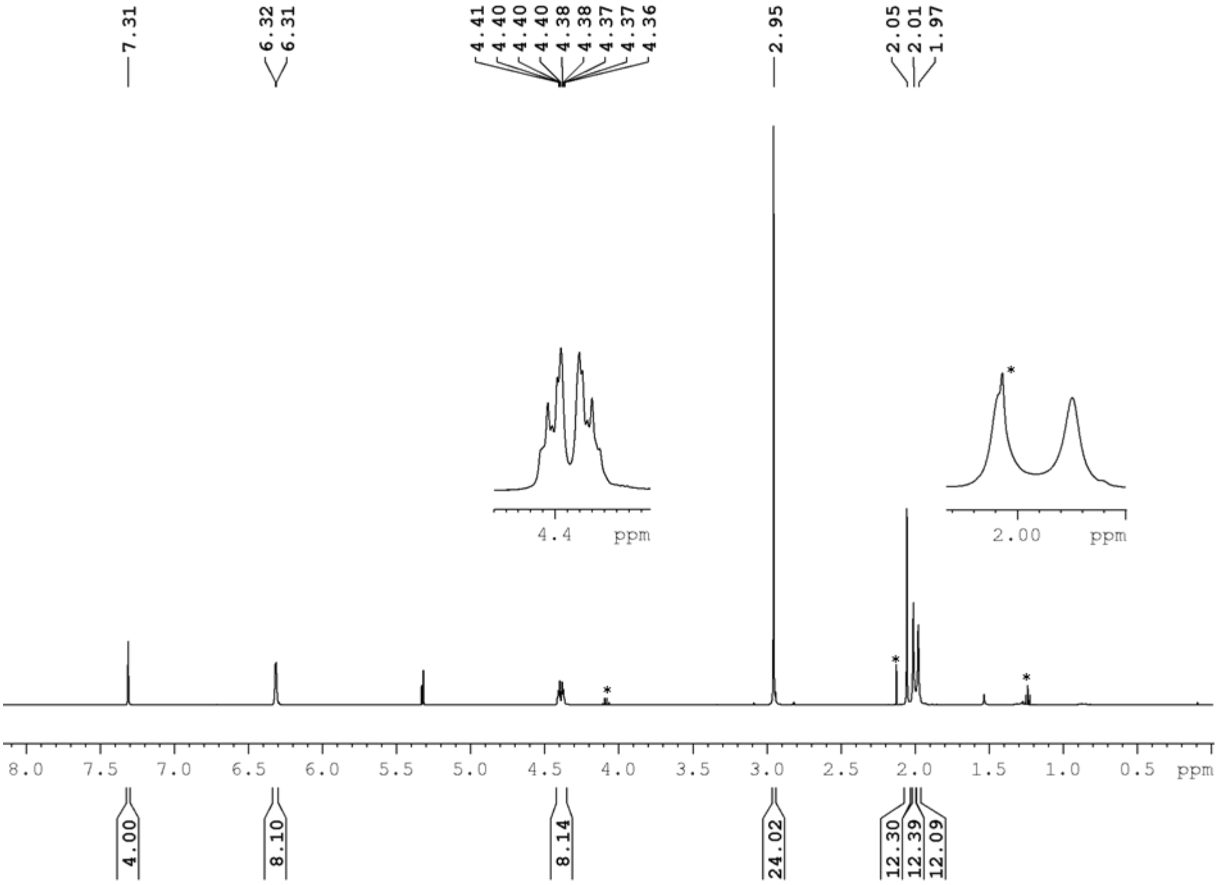

Figure S6. ^{1}H NMR spectrum of **3N** in CD_2Cl_2 at 500 MHz. Residual solvent peaks (EtOAc, acetone) are marked with *.

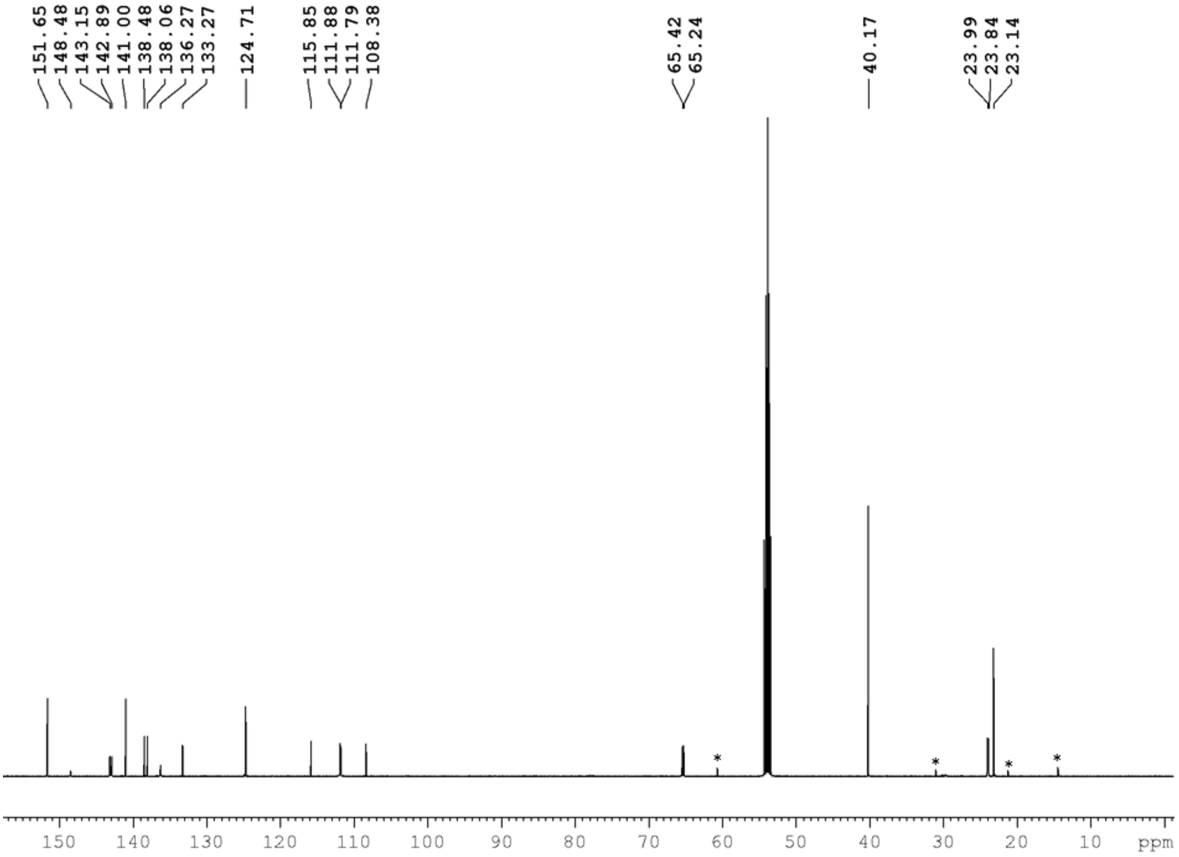

Figure S7. $^{13}C\{^1H\}$ NMR spectrum of **3N** in CD_2Cl_2 at 125 MHz. Residual solvent peaks (EtOAc, acetone) are marked with *.

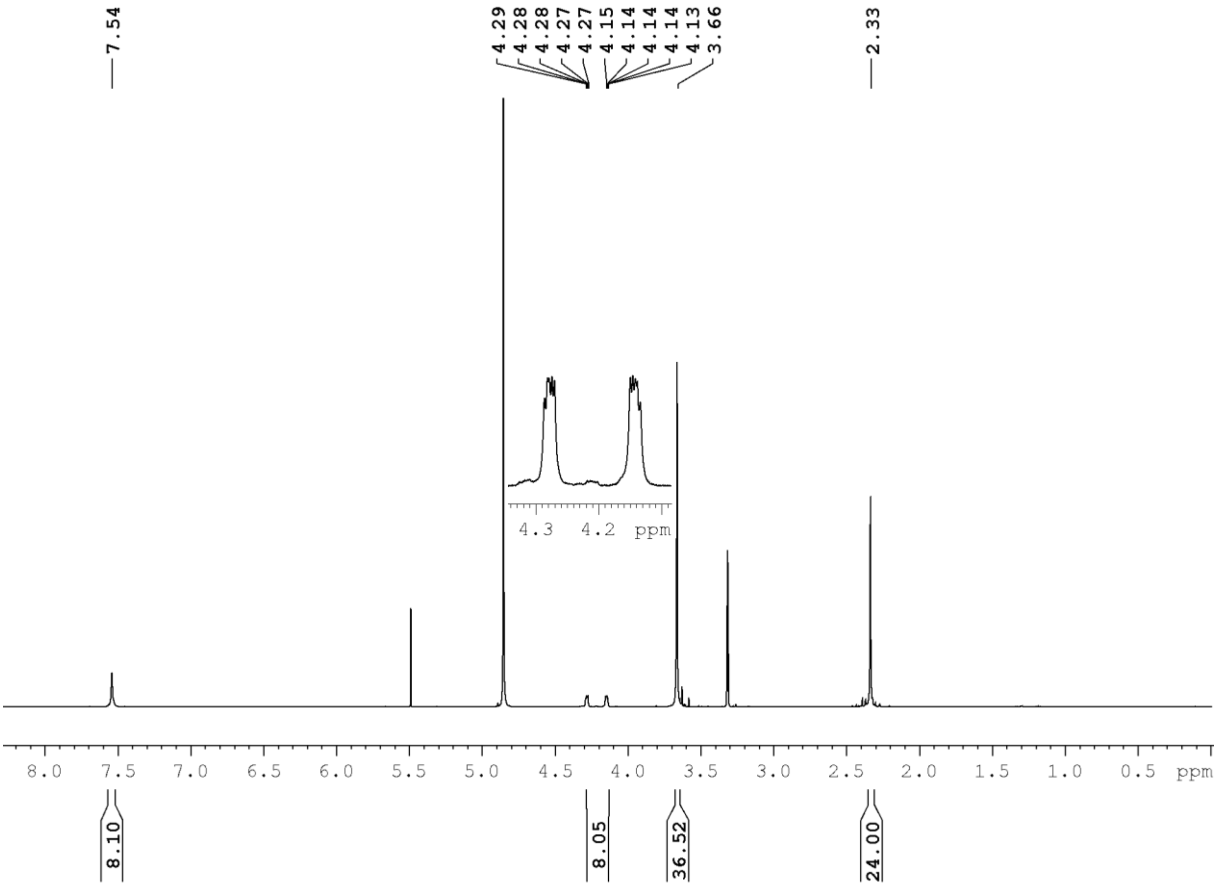

Figure S8. ^1H NMR spectrum of **1** in CD_3OD at 500 MHz.

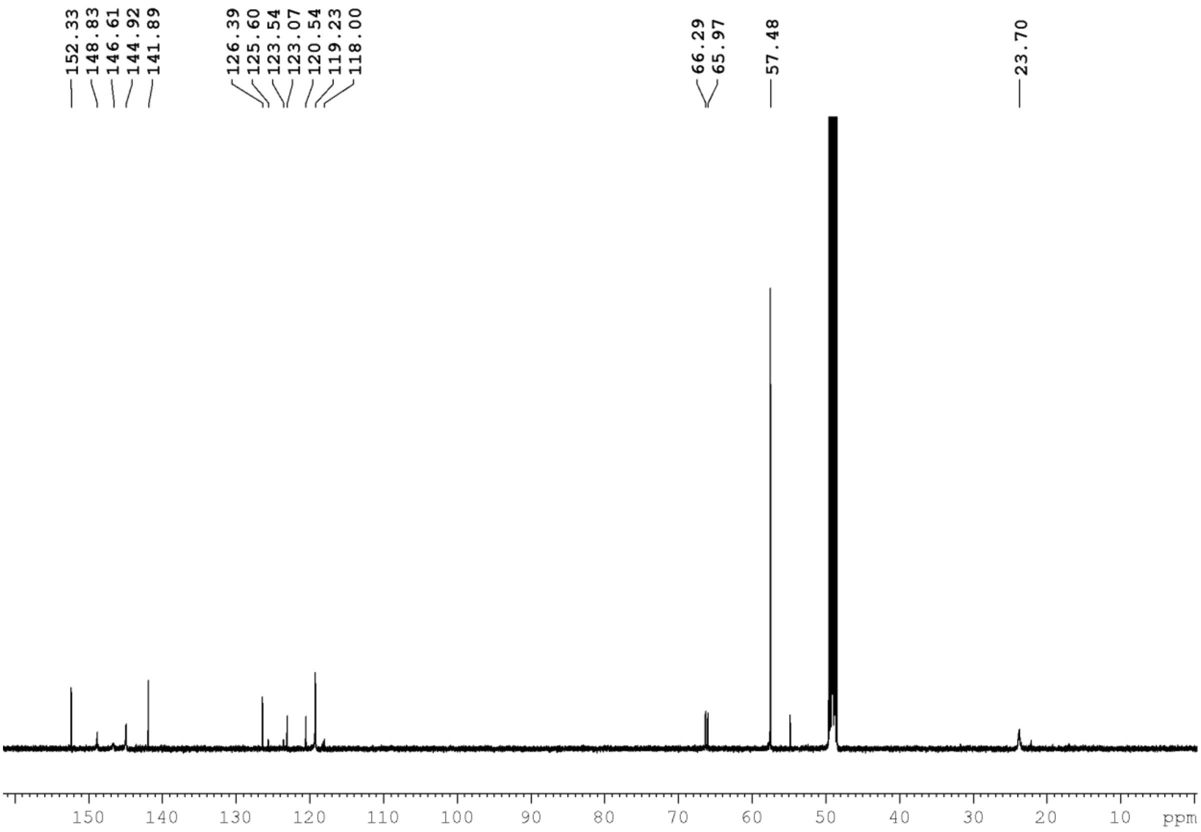

Figure S9. $^{13}C\{^1H\}$ NMR spectrum of **1** in CD_3OD at 125 MHz.

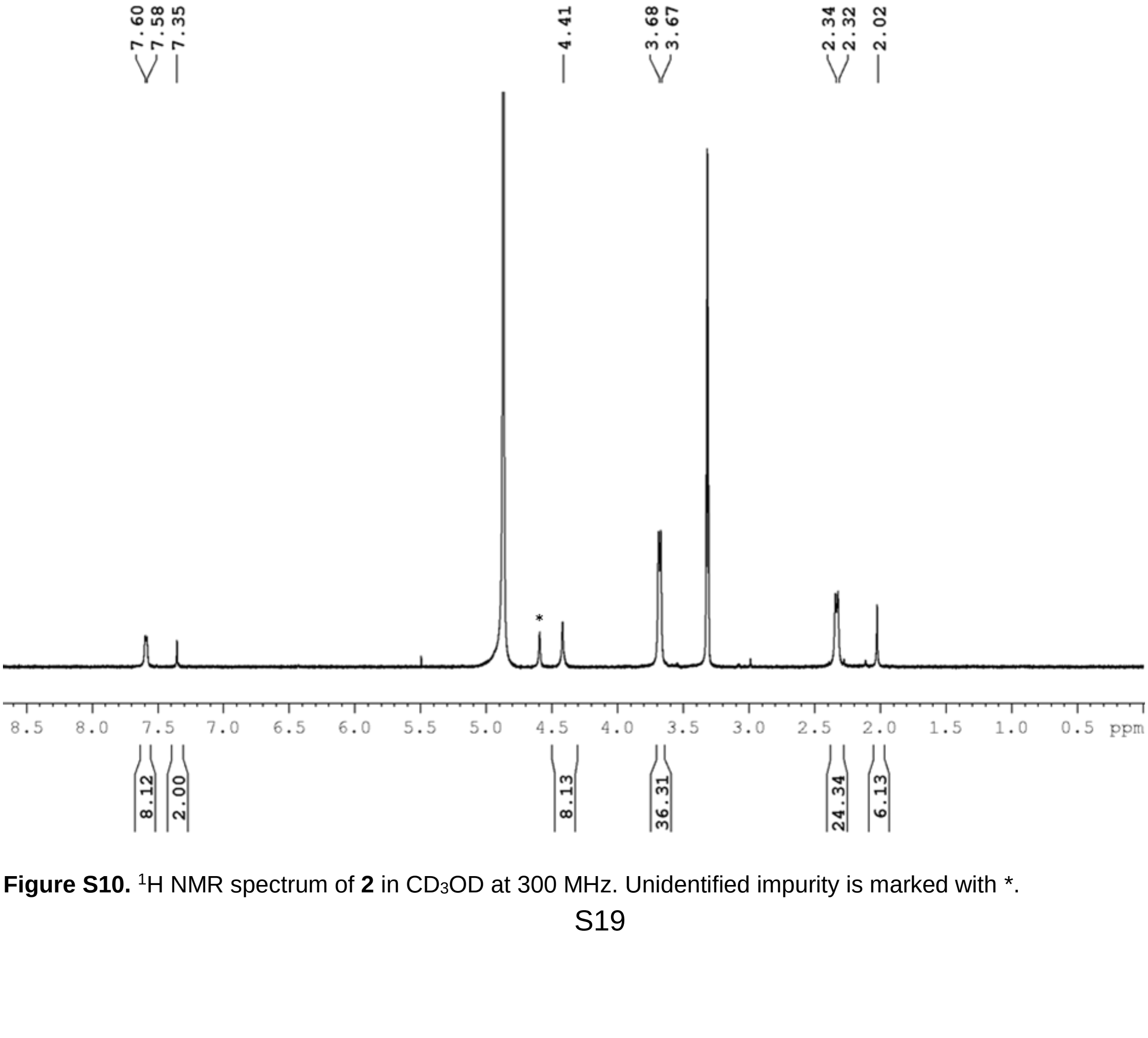

Figure S10. ^1H NMR spectrum of **2** in CD_3OD at 300 MHz. Unidentified impurity is marked with *.

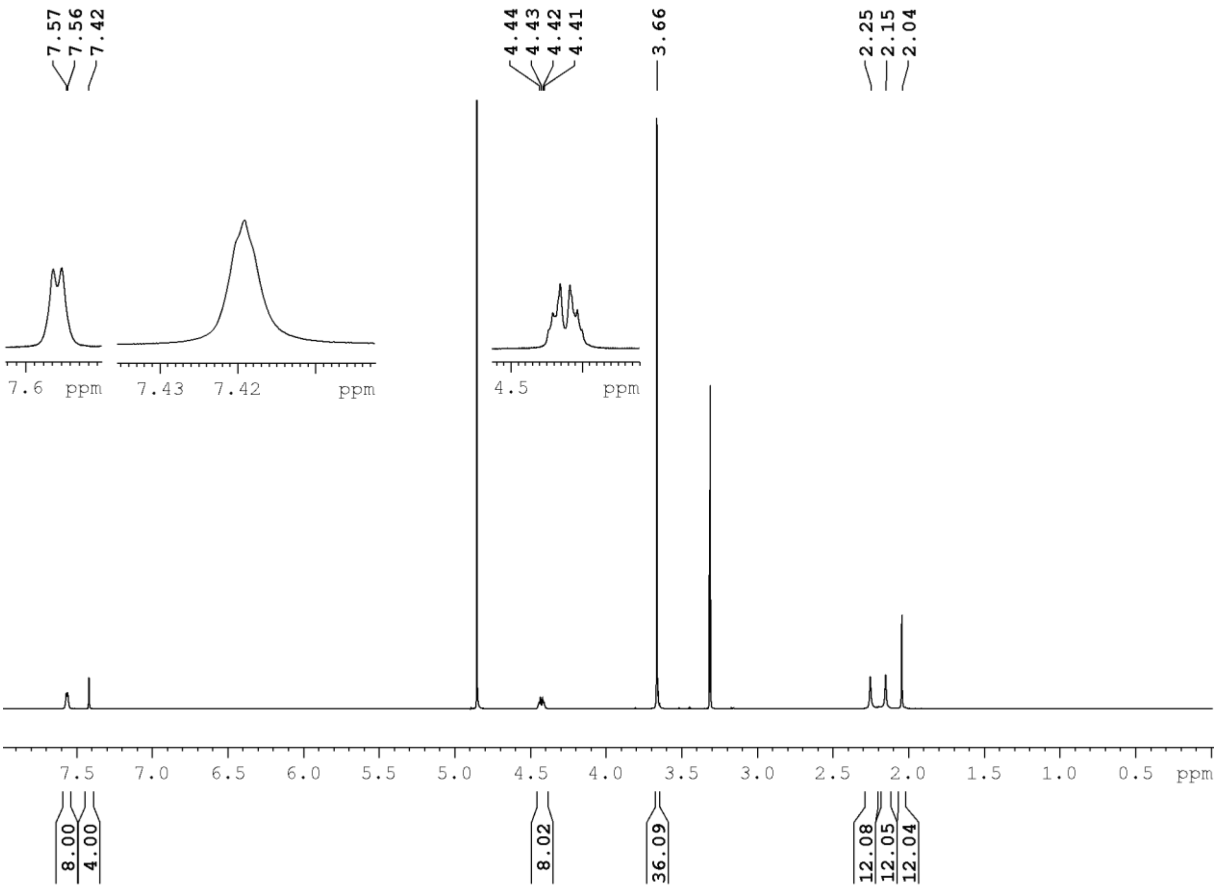

Figure S11. ^{1}H NMR spectrum of **3** in CD_3OD at 500 MHz.

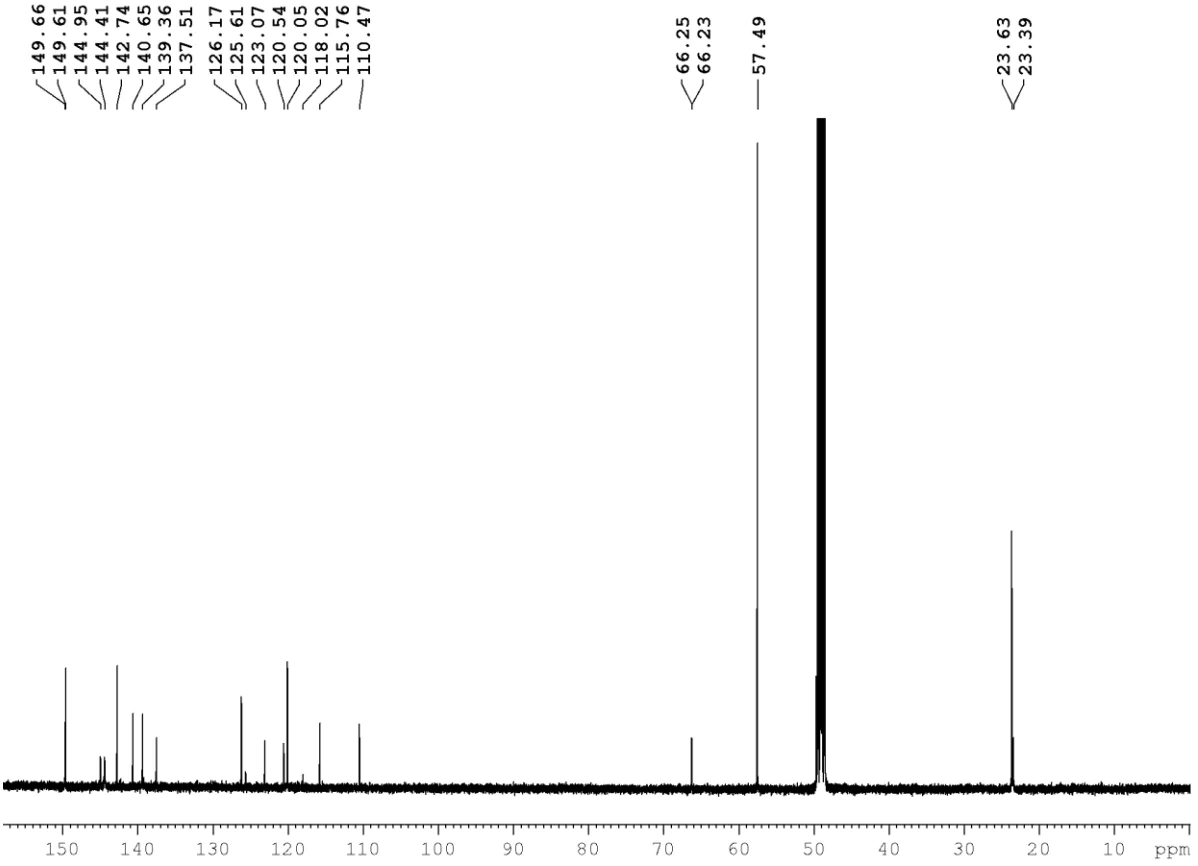

Figure S12. $^{13}C\{^{1}H\}$ NMR spectrum of **3** in CD_3OD at 125 MHz.

Single-Crystal X-Ray Diffraction

Table S1. Single-crystal X-ray diffraction data and structure refinements of compounds **2N**.

Data	**2N**
CCDC number	2164833
Identification code	AF299_MaFe_test_abs
Empirical formula	$C_{65.45}H_{79.89}B_2N_4O_{4.22}S_4$
Formula weight	1140.03
Temperature/K	100(2)
Crystal system	monoclinic
Space group	$P2_1/n$
a/Å	16.72280(10)
b/Å	16.11870(10)
c/Å	46.3545(4)
α/°	90
β/°	93.9260(10)
γ/°	90
Volume/Å^3	12465.52(15)
Z	8
ρ_{calc}g/cm^3	1.215
μ/mm^{-1}	1.789
F(000)	4867.0
Crystal size/mm^3	0.465 × 0.104 × 0.035
Radiation	Cu-K_α (λ = 1.54184)
2Θ range for data collection/°	5.506 to 149.006
Index ranges	-12 ≤ h ≤ 20, -20 ≤ k ≤ 18, -57 ≤ l ≤ 57
Reflections collected	132996
Independent reflections	25334 [R_{int} = 0.0530, R_σ = 0.0345]
Data/restraints/parameters	25334/337/1570
Goodness-of-fit on F^2	1.147
Final R indices [I>=2σ (I)]	R_1 = 0.0755, wR_2 = 0.1848
Final R indices [all data]	R_1 = 0.0837, wR_2 = 0.1898
Largest diff. peak/hole / e Å^{-3}	0.93/-0.62

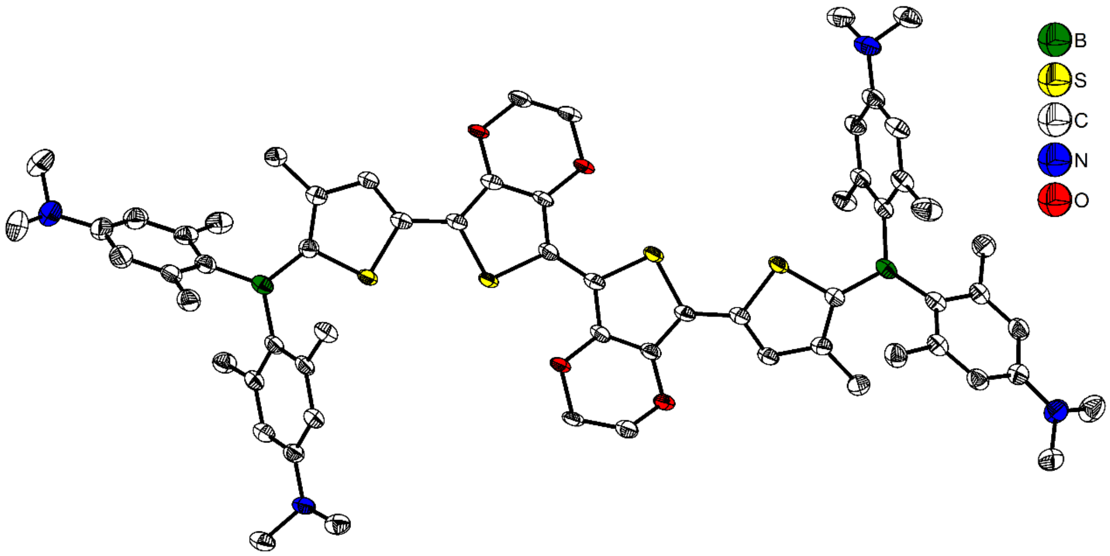

Figure S13. Molecular structure of compound **2N** in the solid state at 100 K. Atomic displacement ellipsoids are drawn at the 50% probability level, and H atoms and co-crystallized solvent molecules (hexane and ethylacetate) are omitted for clarity.

Linear Optical Properties

Table S2. Photophysical data for compounds **1**-**3** in various solvents.

	solvent	λ_{abs} / nm	ε / M^{-1} cm^{-1}	λ_{em} / nm	Stokes shift[a] / cm^{-1}	Φ_f	τ / ns	k_r / 10^8 s^{-1}	k_{nr} / 10^8 s^{-1}
**1**	MeCN	462	53 000	492	1 300	0.03	0.3	1.2	38.8
	H_2O	458		495	1 600	-[b]	-[b]		
**2**[c]	MeCN	536	-[c]	601	2 000	-[c]	-[c]		
	H_2O	526		614	2 700	-[c, b]	-[c, b]		
**3**	MeCN	482	56 000	662	5 600	0.07	0.8	0.9	11.8
	H_2O[a]	465		651	6 100	0.01	< 0.2		

[a] apparent Stokes shift; [b] not measurable due to rapid decomposition; [c] for **2**, only the spectra are given and they should be interpreted with care due to an unidentified impurity. For the same reason, measurements of ε, Φ_f and τ were not performed.

Electrochemistry

Half-wave potentials were determined for all six compounds even though their reduction and oxidation events are not always fully chemically reversible. In particular, the redox events of the sterically less protected compounds **1**, **1’**, **2** and **2’** are accompanied by irreversible processes.

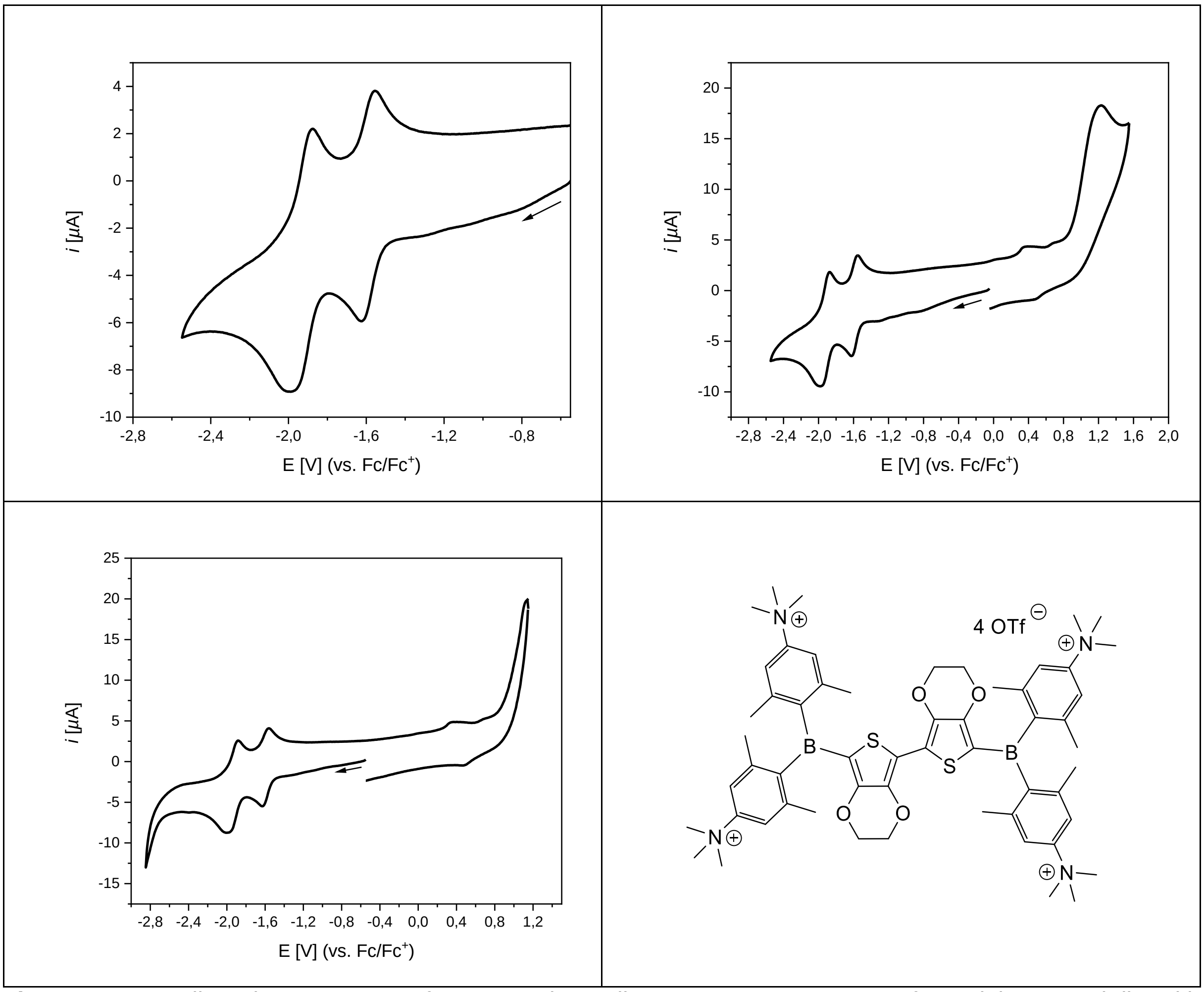

Figure S14. Cyclic voltammograms of compound **1**. All measurements were performed in acetonitrile with [*n*Bu_4N][PF_6] as the electrolyte with a scan rate of 250 mV s^{-1} and are referenced to the Fc/Fc^+ ion couple.

Table S3. Half-wave potentials of partially reversible reduction processes of **1**.

	1st reduction potential $E_{1/2}$ [V] vs. Fc/Fc^+	2nd reduction potential $E_{1/2}$ [V] vs. Fc/Fc^+
**1**	-1.59	-1.93

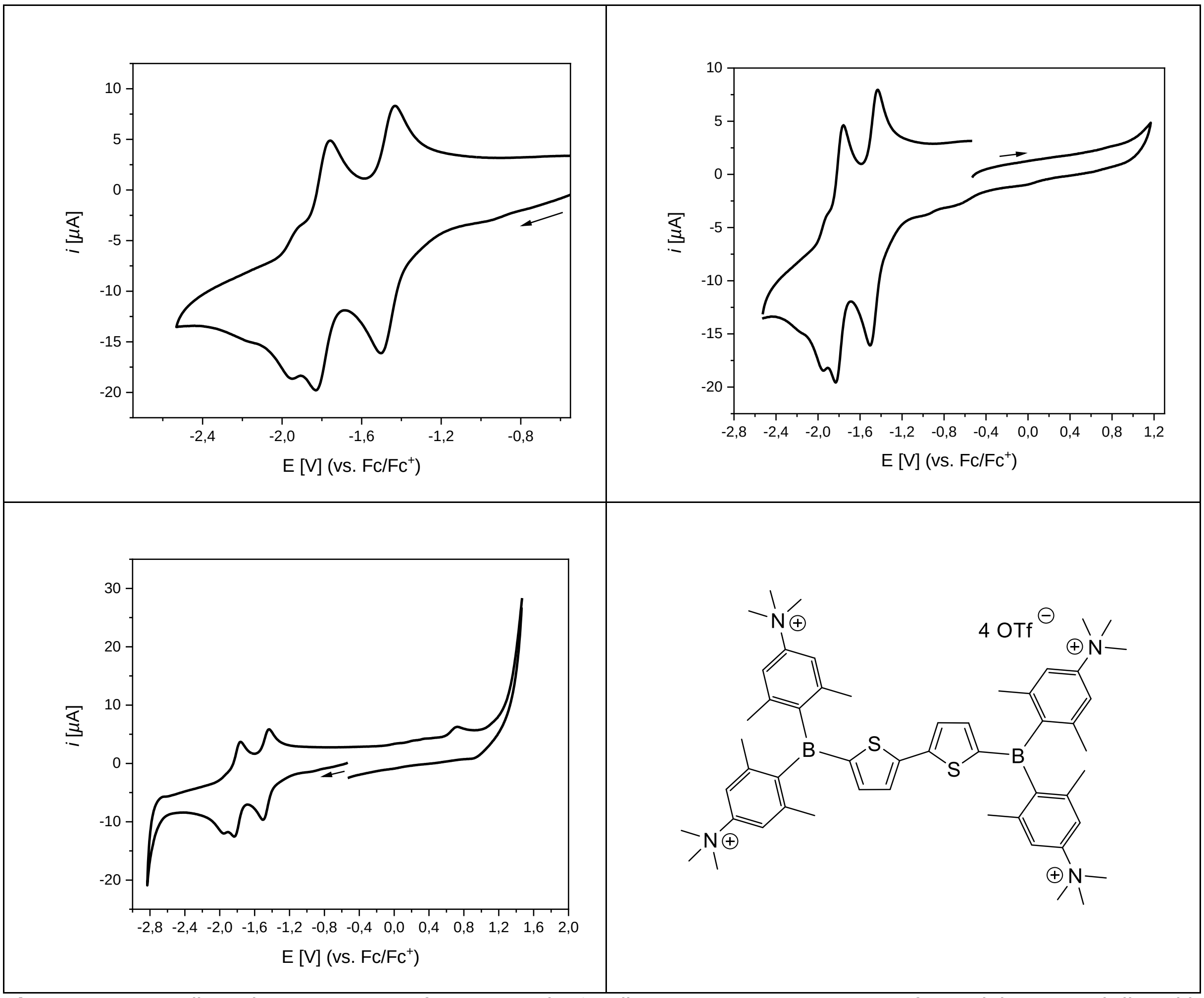

Figure S15. Cyclic voltammograms of compound **1'**. All measurements were performed in acetonitrile with [nBu$_4$N][PF$_6$] as the electrolyte with a scan rate of 250 mV s^{-1} and are referenced to the Fc/Fc$^+$ ion couple.

Table S4. Half-wave potentials of partially reversible reduction processes of **1**'.

	1st reduction potential $E_{1/2}$ [V] vs. Fc/Fc$^+$	2nd reduction potential $E_{1/2}$ [V] vs. Fc/Fc$^+$
**1'**	-1.46	-1.79

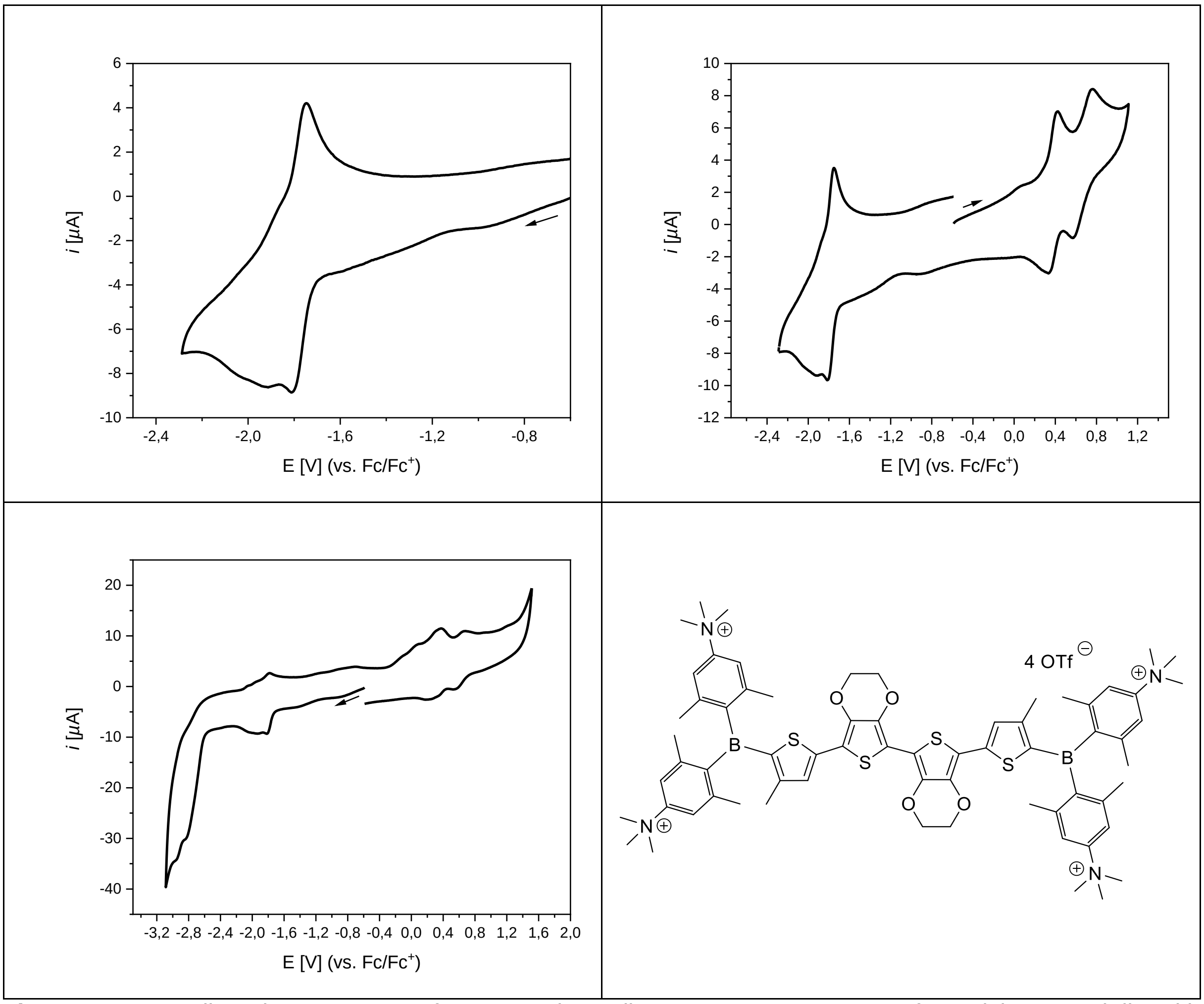

Figure S 16. Cyclic voltammograms of compound **2**. All measurements were performed in acetonitrile with [*n*Bu_4N][PF_6] as the electrolyte with a scan rate of 250 mV s^{-1} and are referenced to the Fc/Fc^+ ion couple.

Table S5. Half-wave potentials of partially reversible reduction and oxidation processes of **2**.

	1st reduction potential $E_{1/2}$ [V] vs. Fc/Fc^+	1st oxidation potential $E_{1/2}$ [V] vs. Fc/Fc^+	2nd oxidation potential $E_{1/2}$ [V] vs. Fc/Fc^+
**2**	-1.78	0.38	0.67

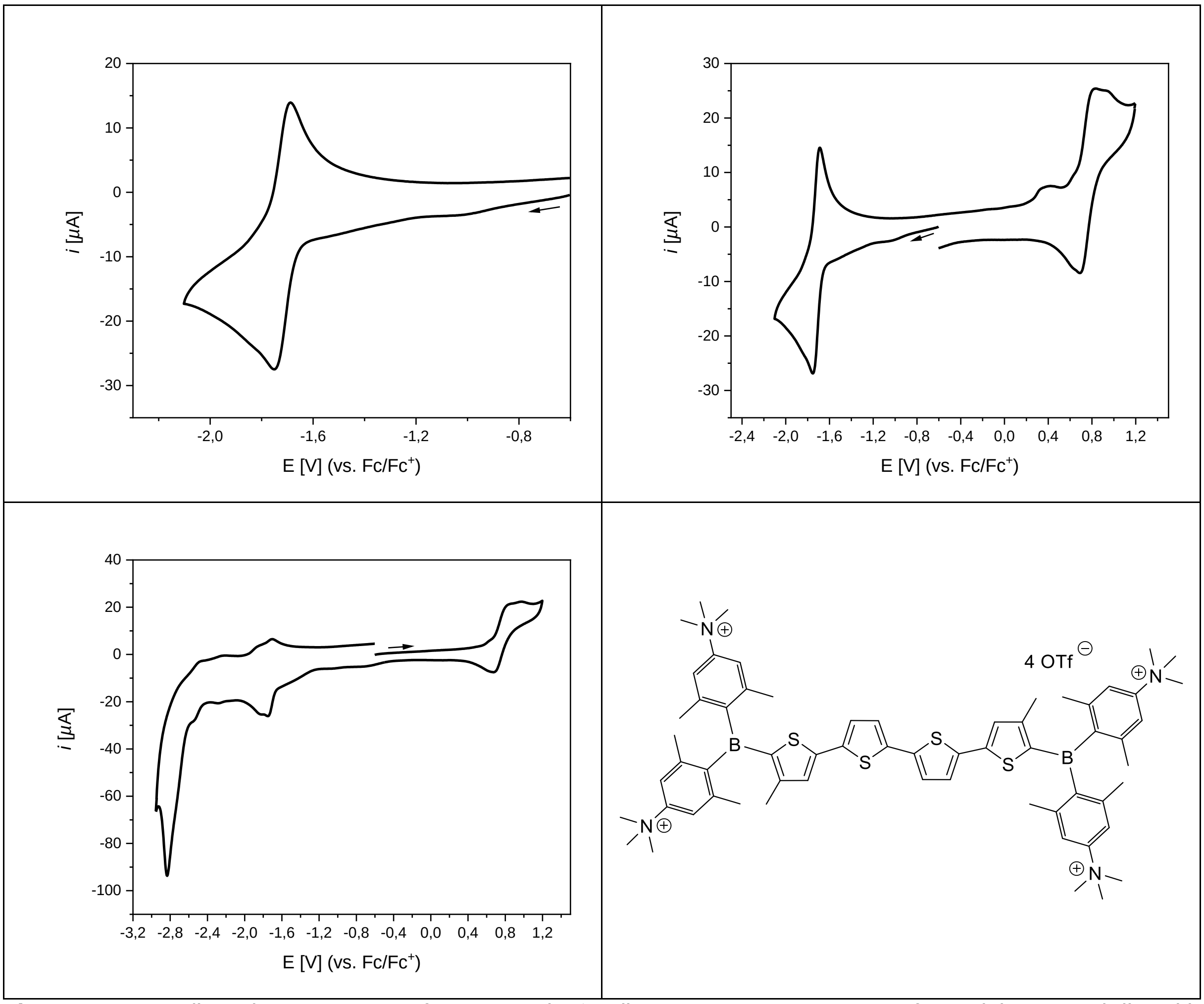

Figure S17. Cyclic voltammograms of compound **2‘**. All measurements were performed in acetonitrile with [*n*Bu_4N][PF_6] as the electrolyte with a scan rate of 250 mV s^{-1} and are referenced to the Fc/Fc^+ ion couple.

Table S6. Half-wave potentials of partially reversible reduction and oxidation processes of **2‘**.

	1st reduction potential $E_{1/2}$ [V] vs. Fc/Fc^+	1st oxidation potential $E_{1/2}$ [V] vs. Fc/Fc^+	2nd oxidation potential $E_{1/2}$ [V] vs. Fc/Fc^+
**2‘**	-1.71	0.76	-

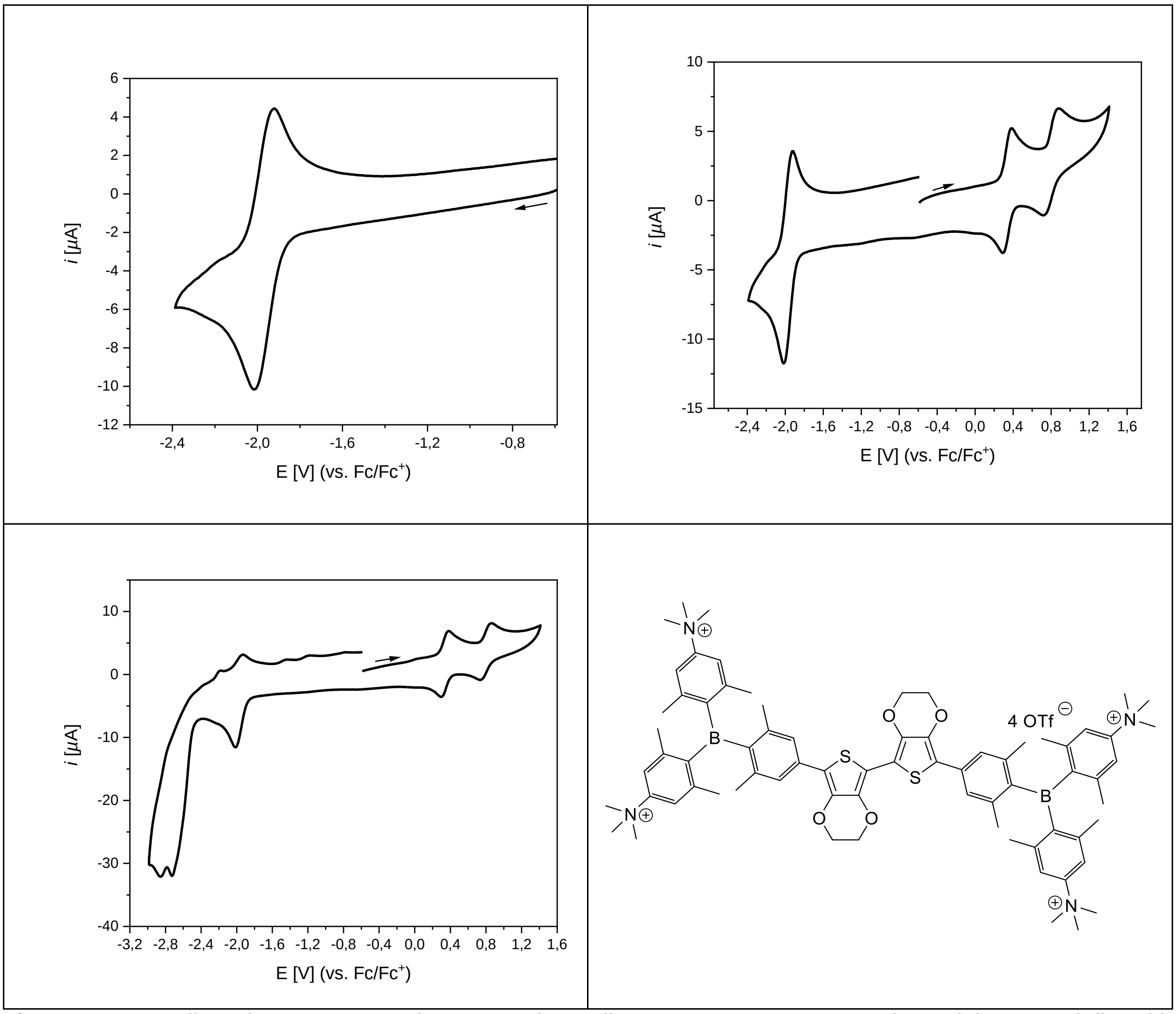

Figure S18. Cyclic voltammograms of compound **3**. All measurements were performed in acetonitrile with [*n*Bu_4N][PF_6] as the electrolyte with a scan rate of 250 mV s^{-1} and are referenced to the Fc/Fc^+ ion couple.

Table S7. Half-wave potentials of partially reversible reduction and oxidation processes of **3**.

	1st reduction potential $E_{1/2}$ [V] vs. Fc/Fc^+	1st oxidation potential $E_{1/2}$ [V] vs. Fc/Fc^+	2nd oxidation potential $E_{1/2}$ [V] vs. Fc/Fc^+
**3**	-1.97	0.34	0.80

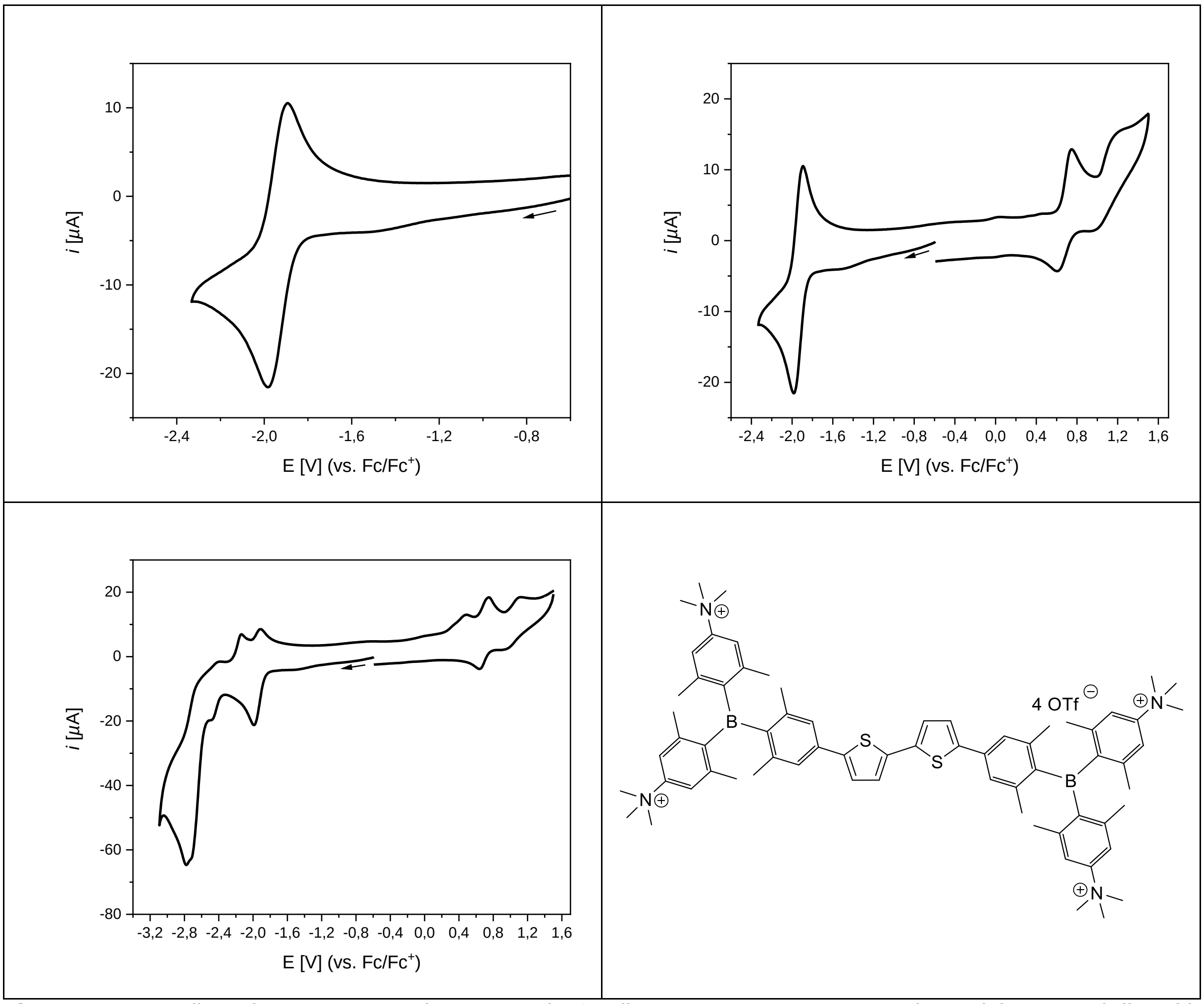

Figure S19. Cyclic voltammograms of compound **3‘**. All measurements were performed in acetonitrile with [nBu$_4$N][PF$_6$] as the electrolyte with a scan rate of 250 mV s^{-1} and are referenced to the Fc/Fc$^+$ ion couple.

Table S8. Half-wave potentials of partially reversible reduction and oxidation processes of **3‘**.

	1st reduction potential $E_{1/2}$ [V] vs. Fc/Fc$^+$	1st oxidation potential $E_{1/2}$ [V] vs. Fc/Fc$^+$	2nd oxidation potential $E_{1/2}$ [V] vs. Fc/Fc$^+$
**3‘**	-1.92	0.68	-

Transient Absorption

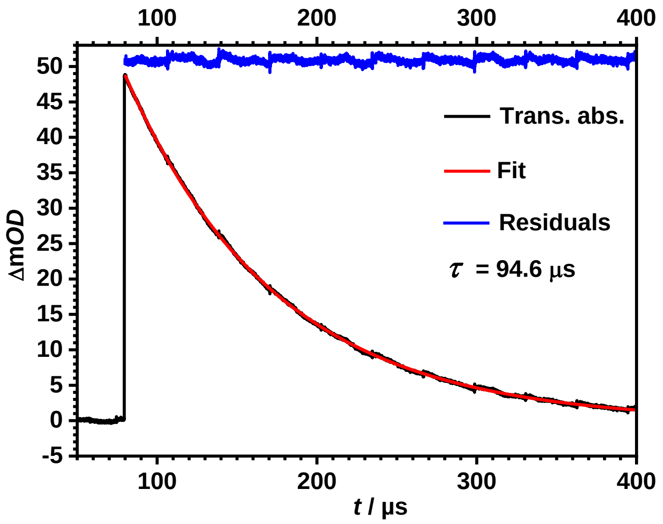

Figure S20. Transient absorption decay curve for compound **3'** at 565 nm (black) and fit (red).

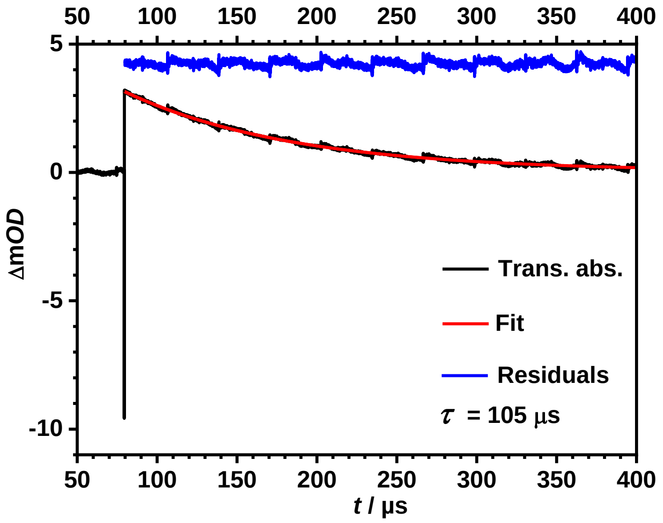

Figure S21. Transient absorption decay curve for compound **3** at 575 nm (black) and fit (red).

Optical Properties in Sodium Cacodylate

All compounds were dissolved in DMSO to give stock solutions of 10^{-3} M. The stock solutions were stored at -8 °C, and working aliquots kept at +25 °C. No visible precipitation or degradation was observed over several months. The experiments were performed in buffer solution (sodium cacodylate buffer, *I* = 0.05 M, pH = 7.0). The absorbancies of **3** buffered solutions were proportional to their concentration within the concentration range employed.

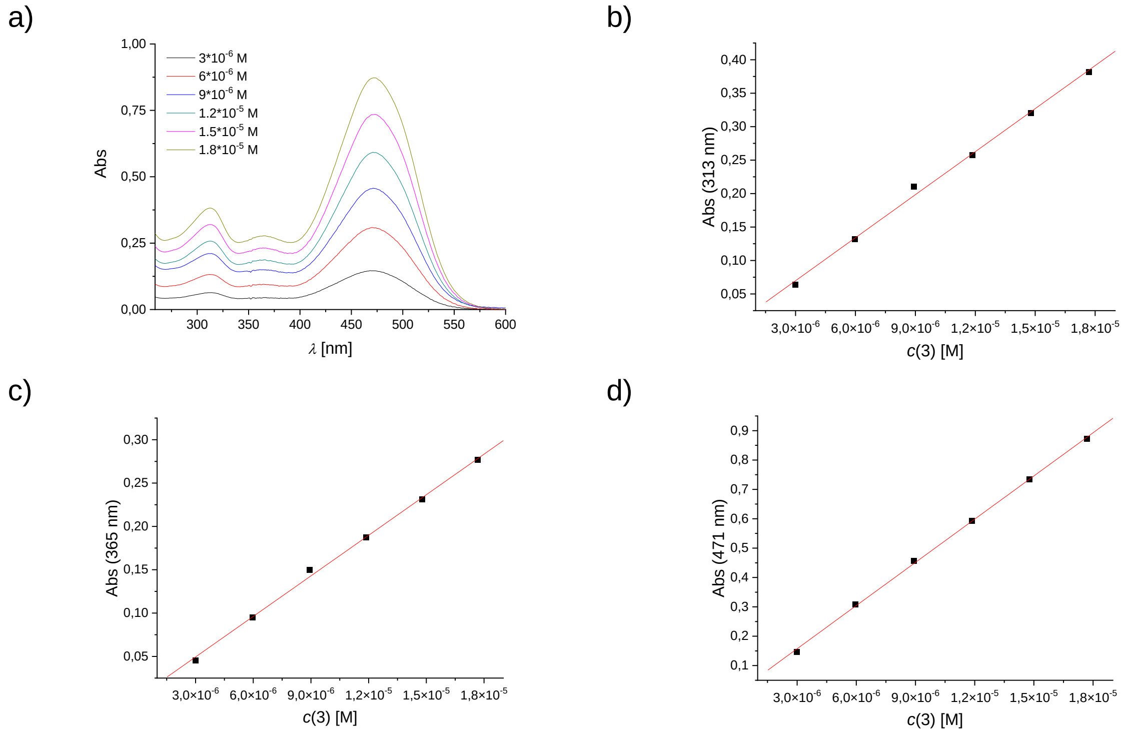

Figure S22. a) Dependence of UV/Vis spectra on concentration of **3**; **b)** Dependence of Abs (313 nm) on *c* (**3**); **c)** Dependence of Abs (365 nm) on *c* (**3**); **d)** Dependence of Abs (471 nm) on *c* (**3**). Done at *pH* 7.0 in sodium cacodylate buffer, *I* = 0.05 M.

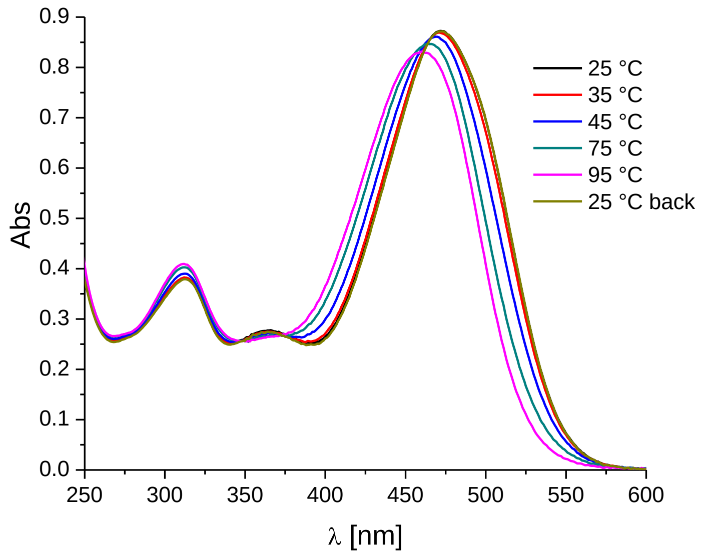

Figure S23. Temperature dependence of UV/Vis spectrum of **3** (c = 1.8 × 10^{-5} M) at pH 7.0, sodium cacodylate buffer, I = 0.05 M.

Study of Interactions with DNA, RNA, and Protein

Table S9. Groove widths and depths for selected nucleic acid conformations. Note the drastic changes accompanied with an A-to-B DNA helix transition.[24]

Structure	Groove width		Groove depth	
type	major	minor	major	minor
A-DNA	2.7	11.0	13.5	2.8
A_nU_n	3.8	10.9	-	-
B-DNA	11.7	5.7	8.5	7.5
$(dGdC)_n$	13.5	9.5	10.0	7.2
$(dAdT)_n$	11.2	6.3	-	-
C-DNA	10.5	4.8	7.5	7.9
A-DNA	11.4	3.3	-	-

Fluorimetric titrations

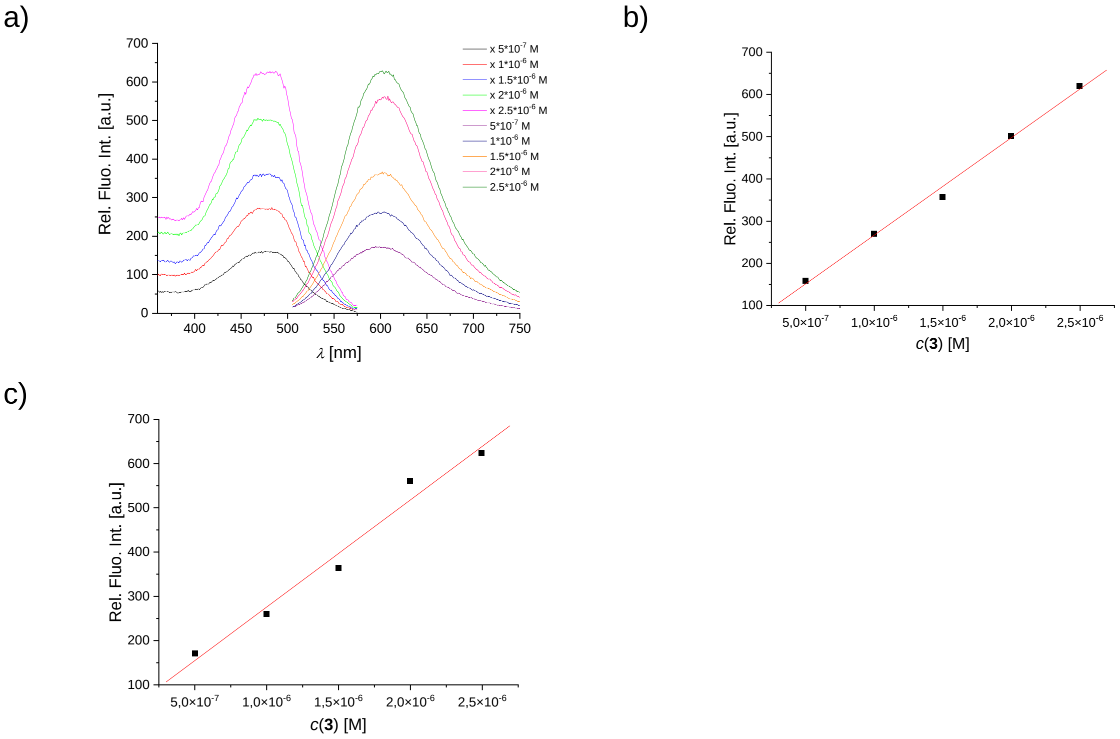

Figure S24. a) Dependence of fluorescence excitation and emission spectra on concentration increase of **3** at λ_{exc} = 471 nm and λ_{em} = 605 nm; **b)** Dependence of fluorescence intensity of excitation spectra on *c*(**3**); **c)** Dependence of fluorescence intensity of emission spectra on *c*(**3**). Done at pH 7.0, sodium cacodylate buffer, *I* = 0.05 M and with slits 2.5-5.

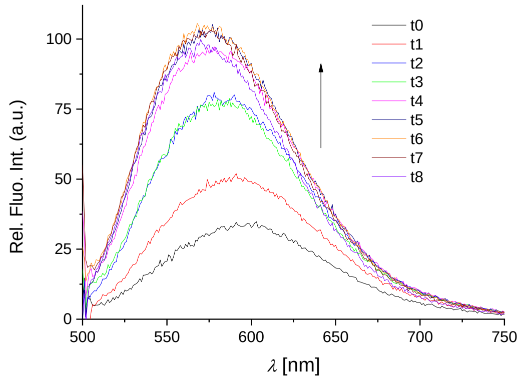

Figure S25. Changes in fluorescence spectrum of **3** (c = 5.0 × 10^{-8} M) on λ_{exc} = 471 nm upon titration with ctDNA; at pH 7.0, sodium cacodylate buffer, I = 0.05 M.

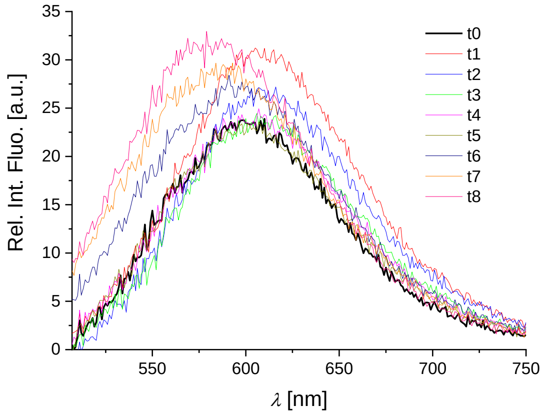

Figure S26. Changes in fluorescence spectrum of **3** (c = 5.0 × 10^{-8} M) on λ_{exc} = 471 nm upon titration with pApU, at pH 7.0, sodium cacodylate buffer, I = 0.05 M.

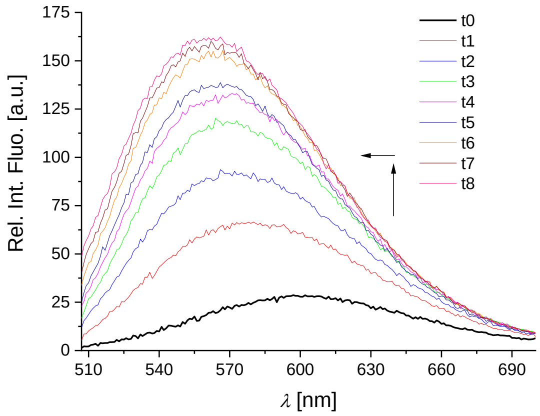

Figure S27. Changes in fluorescence spectrum of **3** (c = 5.0 × 10^{-8} M) on λ_{exc} = 471 nm upon titration with BSA; at pH 7.0, sodium cacodylate buffer, I = 0.05 M.

Circular dichroism (CD) experiments

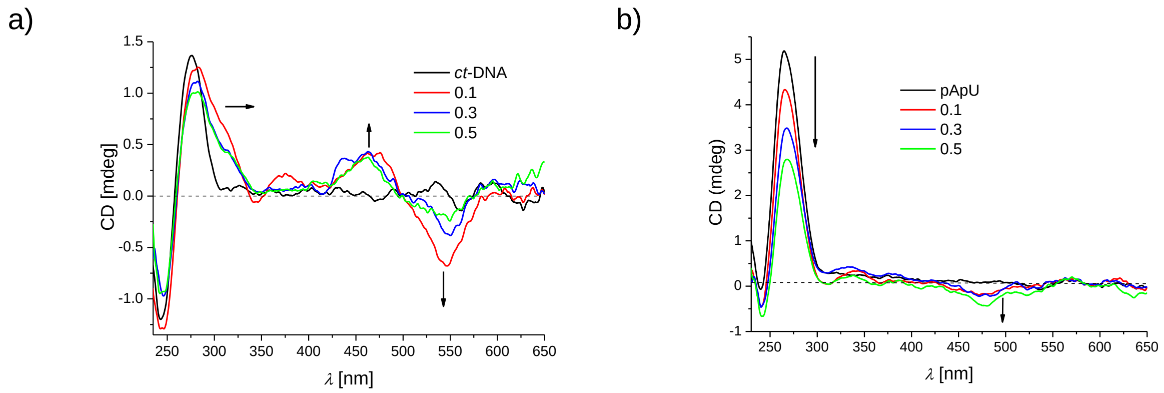

Figure S28. CD titration of **a)** ctDNA, **b)** pApU (c = 2 × 10^{-5} M) with **3** at various molar ratios r = [compound] / [polynucleotide] (*pH* 7.0, buffer sodium cacodylate, *I* = 0.05 M).

Thermal melting experiments

It is well known that, upon heating, ds-helices of polynucleotides at a well-defined temperature (*Tm* value) denature into two single stranded polynucleotides. Non-covalent binding of small molecules to ds-polynucleotides usually has an effect on the thermal stability of helices thus giving different *Tm* values. The difference between the *Tm* value of the free polynucleotide and the complex with a small molecule (Δ*Tm* value) is an important factor in the characterization of small molecule / ds-polynucleotide interactions.[15]

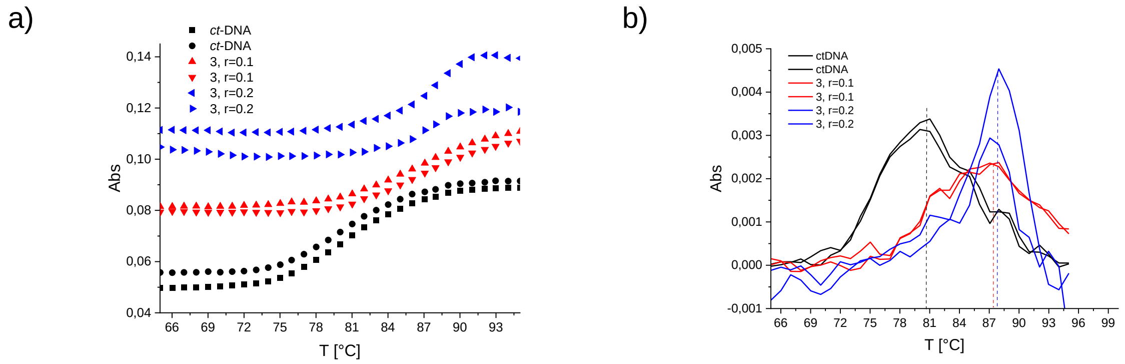

Figure S29. a) Melting curve of ctDNA upon addition r = 0.1 and r = 0.2 ([compound/ [polynucleotide]) of **3** at pH 7.0 (buffer sodium cacodylate, *I* = 0.05 M), **b)** first derivation of absorbance on temperature.

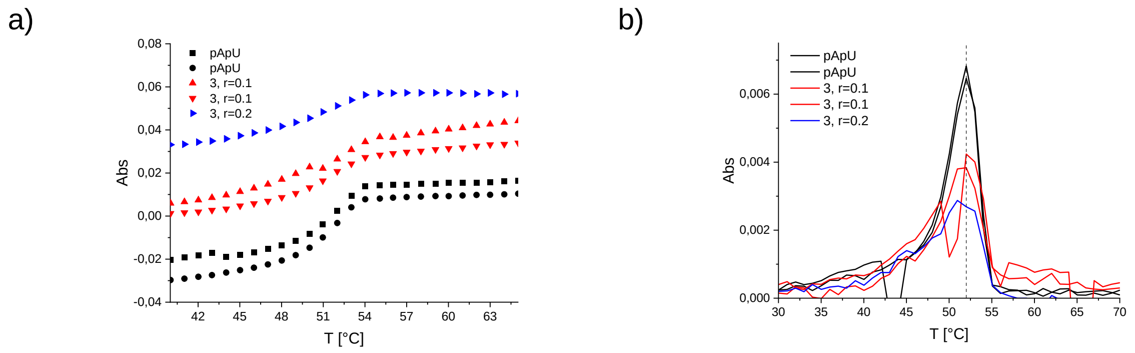

Figure S30. a) Melting curve of pApU upon addition r = 0.1 and r = 0.2 ([compound/ [polynucleotide]) of **3** at pH 7.0 (buffer sodium cacodylate, *I* = 0.05 M), **b)** first derivation of absorbance on temperature.

Theoretical Studies

Compound 1N

<table>
<tr><th rowspan="2">Calculated absorption spectrum</th><th rowspan="2">Orbital</th><th colspan="2">Energy [eV]</th><th rowspan="2">Sym.</th></tr>
<tr><th>gas phase</th><th>toluene</th></tr>
<tr><td rowspan="10">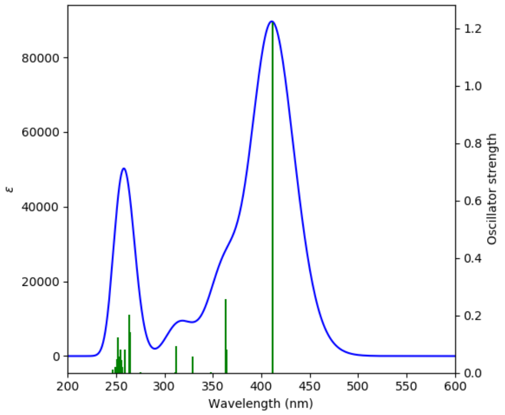

TD-DFT CAM-B3LYP/6-31G+(d,p), gas phase</td><td>L+4</td><td>0.80</td><td>0.80</td><td>A</td></tr>
<tr><td>L+3</td><td>0.77</td><td>0.72</td><td>A</td></tr>
<tr><td>L+2</td><td>0.64</td><td>0.59</td><td>A</td></tr>
<tr><td>L+1</td><td>0.04</td><td>-0.12</td><td>A</td></tr>
<tr><td>LUMO</td><td>-0.94</td><td>-1.11</td><td>A</td></tr>
<tr><td>HOMO</td><td>-6.13</td><td>-6.28</td><td>A</td></tr>
<tr><td>H-1</td><td>-6.27</td><td>-6.40</td><td>A</td></tr>
<tr><td>H-2</td><td>-6.27</td><td>-6.40</td><td>A</td></tr>
<tr><td>H-3</td><td>-6.34</td><td>-6.47</td><td>A</td></tr>
<tr><td>H-4</td><td>-6.61</td><td>-6.75</td><td>A</td></tr>
<tr><th>Orbitals relevant to the $S_1 \leftarrow S_0$ transition</th><th colspan="4">other relevant orbitals</th></tr>
<tr><td>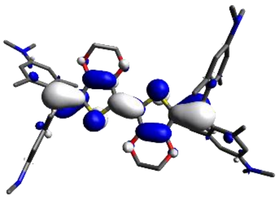
LUMO</td><td>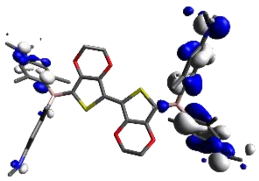
HOMO-1</td><td colspan="3">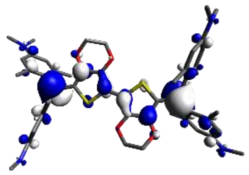
LUMO+1</td></tr>
<tr><td>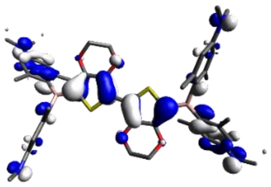
HOMO</td><td>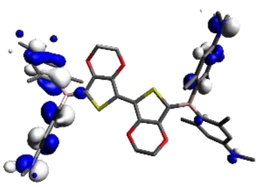
HOMO-2</td><td colspan="3">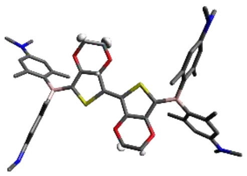
LUMO+2</td></tr>
<tr><td>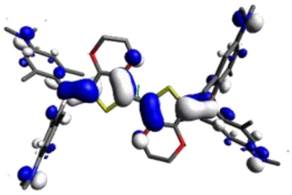
HOMO-4</td><td>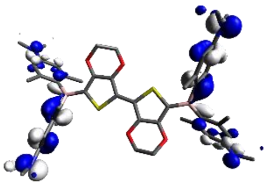
HOMO-3</td><td colspan="3">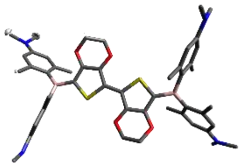
LUMO+3</td></tr>
</table>

Table S10. Lowest energy singlet electronic transition of **1N** (TD-DFT CAM-B3LYP/6-31G+(d,p), gas phase).

State	E [eV]	λ [nm]	*f*	Sym.	Major contributions	Λ
**1**	3.01	412	1.222	A	H-4→LUMO (12%), HOMO→LUMO (77%)	0.63
**2**	3.41	364	0.081	A	H-2→L+1 (12%), H-1→LUMO (69%)	0.36
**3**	3.41	364	0.256	A	H-2→LUMO (70%), H-1→L+1 (13%)	0.36
**4**	3.56	348	0.001	A	H-3→LUMO (68%), HOMO→L+1 (19%)	0.45
**5**	3.77	329	0.055	A	H-4→LUMO (68%), H-3→L+1 (11%)	0.67
**6**	3.97	313	0.093	A	H-5→LUMO (77%)	0.66
**7**	3.98	312	0.004	A	H-6→LUMO (78%)	0.65
**8**	4.21	295	0.000	A	H-4→L+1 (28%), H-3→LUMO (12%), HOMO→L+1 (43%)	0.60
**9**	4.44	279	0.001	A	H-7→LUMO (45%), H-7→L+1 (18%)	0.29
**10**	4.45	279	0.001	A	H-8→LUMO (44%), H-8→L+1 (17%)	0.32
**11**	4.51	275	0.002	A	H-10→LUMO (26%), H-10→L+1 (11%), H-9→LUMO (14%)	0.28
**12**	4.51	275	0.002	A	H-10→LUMO (14%), H-9→LUMO (26%), H-9→L+1 (11%)	0.28
**13**	4.69	264	0.142	A	H-2→L+1 (11%), H-1→LUMO (16%), H-1→L+1 (24%)	0.38
**14**	4.70	264	0.202	A	H-2→LUMO (16%), H-2→L+1 (25%), H-1→L+1 (10%)	0.39
**15**	4.79	259	0.080	A	H-3→L+1 (17%), HOMO→L+3 (12%)	0.38
**16**	4.81	258	0.000	A	H-3→L+3 (14%), H-2→L+5 (11%)	0.33
**17**	4.83	256	0.020	A		0.29
**18**	4.83	256	0.007	A	H-3→L+4 (10%)	0.30
**19**	4.86	255	0.046	A		0.40
**20**	4.87	255	0.080	A	H-8→LUMO (15%)	0.36
**21**	4.88	254	0.055	A	H-3→L+1 (12%)	0.37
**22**	4.93	252	0.124	A	H-10→LUMO (18%)	0.37
**23**	4.93	251	0.048	A	H-9→LUMO (16%)	0.34
**24**	4.97	249	0.021	A	H-11→LUMO (13%), H-4→L+1 (25%), HOMO→L+1 (11%)	0.60
**25**	5.03	247	0.013	A	H-4→L+2 (18%), HOMO→L+2 (25%)	0.35

Table S11. Lowest energy singlet electronic transition of **1N** (TD-DFT CAM-B3LYP/6-31G+(d,p), toluene).

State	E [eV]	λ [nm]	*f*	Sym.	Major contributions	Λ
**1**	2.94	422	1.349	A	H-4→LUMO (15%), HOMO→LUMO (74%)	0.62
**2**	3.37	368	0.092	A	H-2→L+1 (15%), H-1→LUMO (68%)	0.35
**3**	3.37	368	0.360	A	H-2→LUMO (68%), H-1→L+1 (16%)	0.36
**4**	3.51	353	0.001	A	H-3→LUMO (67%), HOMO→L+1 (20%)	0.44
**5**	3.72	333	0.072	A	H-5→LUMO (11%), H-4→LUMO (65%), H-3→L+1 (11%)	0.68
**6**	3.93	315	0.135	A	H-5→LUMO (77%)	0.66
**7**	3.95	314	0.001	A	H-6→LUMO (80%)	0.66
**8**	4.18	297	0.000	A	H-4→L+1 (31%), H-3→LUMO (13%), HOMO→L+1 (40%)	0.60
**9**	4.45	279	0.001	A	H-7→LUMO (42%), H-7→L+1 (17%)	0.31
**10**	4.45	279	0.001	A	H-8→LUMO (41%), H-8→L+1 (16%)	0.32
**11**	4.51	275	0.007	A	H-9→LUMO (34%), H-9→L+1 (12%)	0.28
**12**	4.51	275	0.006	A	H-10→LUMO (34%), H-10→L+1 (13%)	0.29
**13**	4.66	266	0.213	A	H-2→L+1 (11%), H-1→LUMO (18%), H-1→L+1 (26%)	0.38
**14**	4.67	265	0.270	A	H-2→LUMO (18%), H-2→L+1 (27%), H-1→L+1 (11%)	0.40
**15**	4.77	260	0.131	A	H-3→L+1 (31%), HOMO→L+3 (10%)	0.50
**16**	4.81	258	0.023	A	H-7→LUMO (13%)	0.37
**17**	4.82	257	0.056	A	H-8→LUMO (15%)	0.34
**18**	4.85	256	0.055	A	H-3→L+3 (12%)	0.40
**19**	4.88	254	0.091	A	H-9→LUMO (18%)	0.31
**20**	4.88	254	0.154	A	H-10→LUMO (13%)	0.33
**21**	4.90	253	0.035	A	H-10→LUMO (10%)	0.33
**22**	4.91	253	0.008	A	H-1→L+6 (11%)	0.36
**23**	4.91	252	0.107	A		0.34
**24**	4.96	250	0.016	A	H-11→LUMO (10%), H-4→L+1 (20%), HOMO→L+1 (11%)	0.55
**25**	5.03	247	0.005	A	H-11→LUMO (38%), H-5→L+1 (27%), H-4→L+1 (11%)	0.64

Compound 2N

Calculated absorption spectrum

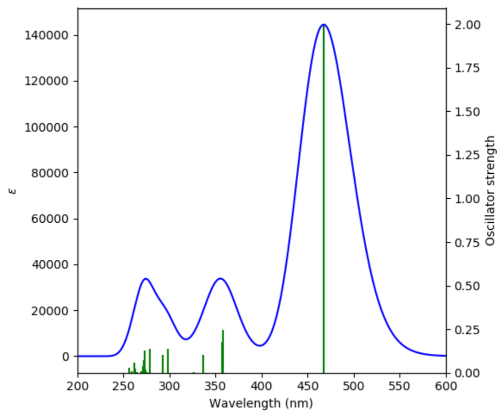

TD-DFT CAM-B3LYP/6-31G+(d,p), gas phase

Orbital	Energy [eV] gas phase	Energy [eV] toluene	Sym.
L+4	0.71	0.64	A
L+3	0.67	0.59	A
L+2	0.21	0.06	A
L+1	-0.43	-0.59	A
LUMO	-1.08	-1.25	A
HOMO	-5.76	-5.92	A
H-1	-6.31	-6.43	A
H-2	-6.32	-6.43	A
H-3	-6.37	-6.48	A
H-4	-6.44	-6.54	A

Orbitals relevant to the $S_1 \leftarrow S_o$ transition

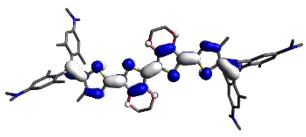

LUMO

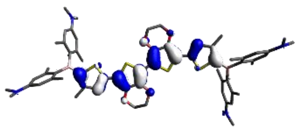

HOMO

other relevant orbitals

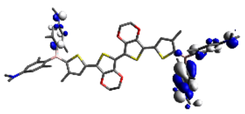

HOMO-1

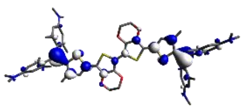

LUMO+1

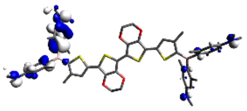

HOMO-2

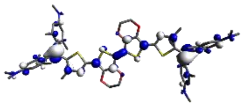

LUMO+2

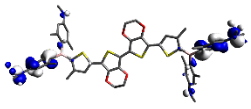

HOMO-3

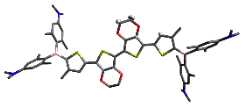

LUMO+3

Table S12. Lowest energy singlet electronic transition of **2N** (TD-DFT CAM-B3LYP/6-31G+(d,p), gas phase).

State	E [eV]	λ [nm]	*f*	Sym.	Major contributions	Λ
**1**	2.65	467	1.993	A	HOMO→LUMO (88%)	0.76
**2**	3.35	370	0.000	A	H-5→LUMO (11%), H-3→LUMO (15%), HOMO→L+1 (49%)	0.56
**3**	3.46	358	0.244	A	H-2→LUMO (14%), H-1→LUMO (29%), H-1→L+1 (29%)	0.33
**4**	3.47	357	0.179	A	H-2→LUMO (30%), H-2→L+1 (30%)	0.32
**5**	3.69	336	0.104	A	H-4→LUMO (45%), H-3→L+1 (27%)	0.45
**6**	3.80	326	0.002	A	H-4→L+1 (26%), H-3→LUMO (29%), HOMO→L+1 (23%)	0.49
**7**	4.11	302	0	A	H-5→LUMO (66%), HOMO→L+1 (10%)	0.73
**8**	4.16	298	0.139	A	H-5→L+1 (15%), H-4→LUMO (10%), HOMO→L+2 (55%)	0.64
**9**	4.24	293	0.100	A	H-6→LUMO (76%)	0.63
**10**	4.28	290	0.000	A	H-11→LUMO (25%), H-9→LUMO (38%)	0.59
**11**	4.46	278	0.136	A	H-13→L+1 (13%), H-12→LUMO (39%), H-10→LUMO (11%)	0.56
**12**	4.50	276	0.000	A	H-7→LUMO (20%), H-7→L+1 (16%)	0.23
**13**	4.50	275	0.002	A	H-8→LUMO (20%), H-8→L+1 (16%)	0.24
**14**	4.53	274	0.002	A	H-10→L+1 (17%)	0.49
**15**	4.53	274	0.017	A	H-13→LUMO (10%), H-1→LUMO (14%)	0.49
**16**	4.54	273	0.129	A	H-12→LUMO (11%)	0.44
**17**	4.56	272	0.074	A	H-2→LUMO (20%), H-2→L+2 (10%), H-1→LUMO (10%), H-1→L+1 (11%)	0.34
**18**	4.57	271	0.037	A	H-13→LUMO (15%), H-2→LUMO (12%), H-2→L+1 (10%), H-1→LUMO (15%)	0.43
**19**	4.58	270	0.011	A	HOMO→L+3 (20%), HOMO→L+20 (15%)	0.28
**20**	4.61	269	0.008	A	H-13→LUMO (30%), H-12→L+1 (14%)	0.56
**21**	4.69	265	0.002	A	HOMO→L+19 (17%), HOMO→L+25 (14%)	0.35
**22**	4.71	263	0.023	A	H-4→LUMO (10%), HOMO→L+16 (13%)	0.33
**23**	4.74	261	0.057	A	H-4→LUMO (22%), H-4→L+2 (15%), H-3→L+1 (13%)	0.44
**24**	4.79	259	0.007	A	H-4→L+1 (14%), H-3→LUMO (18%)	0.46
**25**	4.85	256	0.027	A	H-1→L+8 (16%)	0.27

Table S13. Lowest energy singlet electronic transition of **2N** (TD-DFT CAM-B3LYP/6-31G+(d,p), toluene).

State	E [eV]	λ [nm]	*f*	Sym.	Major contributions
**1**	2.57	482	2.082	A	HOMO→LUMO (88%)
**2**	3.27	379	0.000	A	H-5→LUMO (13%), H-3→LUMO (19%), HOMO→L+1 (50%)
**3**	3.41	364	0.278	A	H-1→LUMO (42%), H-1→L+1 (32%), H-1→L+2 (10%)
**4**	3.41	363	0.262	A	H-2→LUMO (42%), H-2→L+1 (32%), H-2→L+2 (10%)
**5**	3.62	343	0.184	A	H-4→LUMO (45%), H-3→L+1 (29%)
**6**	3.76	330	0.002	A	H-4→L+1 (28%), H-3→LUMO (32%), HOMO→L+1 (20%)
**7**	4.07	304	0.000	A	H-5→LUMO (61%), HOMO→L+1 (13%)
**8**	4.12	301	0.284	A	H-5→L+1 (17%), H-4→LUMO (12%), HOMO→L+2 (47%)
**9**	4.19	296	0.096	A	H-6→LUMO (72%)
**10**	4.26	291	0.000	A	H-11→LUMO (39%), H-9→LUMO (24%)
**11**	4.44	279	0.134	A	H-13→L+1 (14%), H-12→LUMO (42%)
**12**	4.49	276	0.010	A	H-7→LUMO (10%)
**13**	4.49	276	0.051	A	H-1→LUMO (25%), H-1→L+2 (14%)
**14**	4.50	276	0.022	A	H-8→LUMO (13%), H-8→L+1 (10%)
**15**	4.50	275	0.284	A	H-2→LUMO (22%), H-2→L+2 (12%)
**16**	4.51	275	0.003	A	H-9→LUMO (10%), HOMO→L+3 (15%)
**17**	4.54	273	0.001	A	H-10→LUMO (13%)
**18**	4.54	273	0.030	A	H-13→LUMO (16%), H-11→LUMO (11%)
**19**	4.58	270	0.009	A	H-13→LUMO (36%), H-12→L+1 (16%)
**20**	4.60	270	0.010	A	HOMO→L+4 (17%), HOMO→L+21 (21%)
**21**	4.66	266	0.002	A	HOMO→L+17 (21%), HOMO→L+22 (11%)
**22**	4.68	265	0.072	A	H-4→LUMO (30%), H-4→L+2 (20%), H-3→L+1 (19%)
**23**	4.73	262	0.007	A	HOMO→L+4 (14%), HOMO→L+16 (14%)
**24**	4.76	260	0.003	A	H-4→L+1 (16%), H-3→LUMO (18%), HOMO→L+3 (11%)
**25**	4.85	256	0.032	A	H-14→LUMO (11%), H-7→LUMO (11%)

Compound 3N

Calculated absorption spectrum

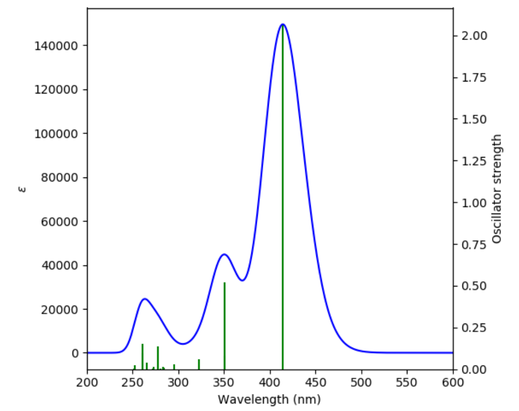

TD-DFT CAM-B3LYP/6-31G+(d,p), gas phase

Orbital	Energy [eV] gas phase	Energy [eV] toluene	Sym.
L+4	0.67	0.62	A
L+3	0.66	0.61	A
L+2	0.10	-0.05	A
L+1	-0.28	-0.42	A
LUMO	-0.70	-0.88	A
HOMO	-5.77	-5.96	A
H-1	-6.31	-6.40	A
H-2	-6.31	-6.40	A
H-3	-6.49	-6.58	A
H-4	-6.51	-6.59	A

Orbitals relevant to the $S_1 \leftarrow S_0$ transition

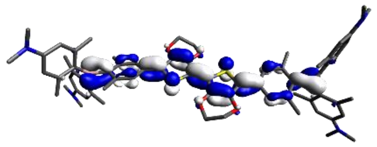

LUMO

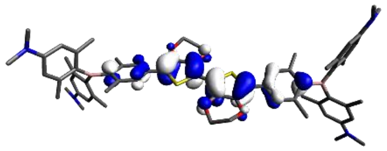

HOMO

other relevant orbitals

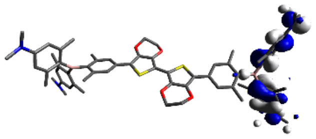

HOMO-1

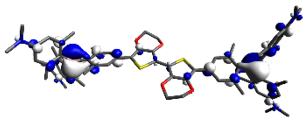

LUMO+1

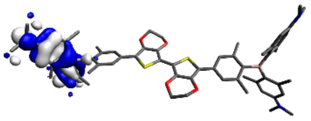

HOMO-2

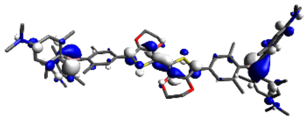

LUMO+2

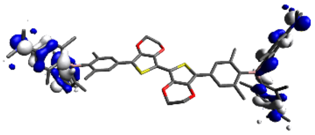

HOMO-3

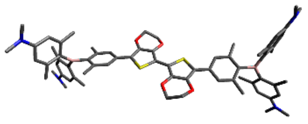

LUMO+3

Table S14. Lowest energy singlet electronic transition of **3N** (TD-DFT CAM-B3LYP/6-31G+(d,p), gas phase).

State	E [eV]	λ [nm]	*f*	Sym.	Major contributions	Λ
**1**	2.99	414	2.062	A	HOMO→LUMO (84%)	0.71
**2**	3.54	350	0.080	A	H-2→LUMO (19%), H-2→L+1 (15%), H-2→L+2 (11%), H-1→LUMO (12%), H-1→L+1 (26%)	0.34
**3**	3.54	350	0.523	A	H-2→LUMO (12%), H-2→L+1 (26%), H-1→LUMO (19%), H-1→L+1 (15%), H-1→L+2 (11%)	0.33
**4**	3.63	342	0	A	H-5→LUMO (11%), H-4→L+1 (11%), H-3→LUMO (14%), HOMO→L+1 (43%)	0.49
**5**	3.84	323	0.061	A	H-4→LUMO (24%), H-4→L+2 (10%), H-3→L+1 (32%), HOMO→L+2 (18%)	0.51
**6**	4.04	307	0	A	H-4→L+1 (29%), H-3→LUMO (22%), HOMO→L+1 (24%)	0.48
**7**	4.20	295	0.030	A	H-5→L+1 (16%), H-4→LUMO (12%), HOMO→L+2 (43%)	0.54
**8**	4.37	284	0.006	A	H-7→LUMO (25%), H-5→LUMO (24%)	0.64
**9**	4.38	283	0.012	A	H-11→L+1 (11%), H-10→LUMO (17%), H-8→LUMO (10%), H-6→LUMO (28%)	0.52
**10**	4.42	280	0.003	A	H-11→LUMO (21%), H-5→LUMO (26%)	0.59
**11**	4.46	278	0.137	A	H-10→LUMO (10%), H-6→LUMO (47%)	0.55
**12**	4.54	273	0.003	A	H-9→LUMO (10%), H-9→L+2 (10%), H-8→L+1 (14%)	0.48
**13**	4.54	273	0.013	A	H-9→L+1 (17%)	0.47
**14**	4.55	272	0.000	A	H-12→L+1 (17%)	0.45
**15**	4.56	272	0.002	A	H-13→L+1 (20%), H-12→LUMO (11%), H-12→L+2 (11%)	0.43
**16**	4.59	270	0.000	A	H-5→L+2 (11%), HOMO→L+5 (20%)	0.52
**17**	4.66	266	0.000	A	H-7→LUMO (39%)	0.62
**18**	4.67	266	0.038	A	HOMO→L+3 (14%), HOMO→L+7 (31%)	0.21
**19**	4.76	261	0.097	A	H-2→LUMO (23%), H-2→L+2 (12%), H-1→LUMO (16%)	0.28
**20**	4.76	261	0.154	A	H-2→LUMO (16%), H-1→LUMO (22%), H-1→L+2 (12%)	0.28
**21**	4.84	256	0.000	A	H-4→L+6 (13%), H-2→L+3 (13%), H-1→L+4 (15%)	0.27
**22**	4.85	256	0	A	H-3→L+6 (13%), H-2→L+4 (15%), H-1→L+3 (13%)	0.27
**23**	4.91	252	0	A	H-4→L+4 (15%), H-3→L+3 (14%), H-1→L+6 (10%)	0.24
**24**	4.91	252	0.022	A	H-4→L+3 (14%), H-3→L+4 (16%), H-2→L+6 (10%)	0.24
**25**	4.93	251	0.001	A	HOMO→L+9 (33%)	0.32

Table S15. Lowest energy singlet electronic transition of **3N** (TD-DFT CAM-B3LYP/6-31G+(d,p), toluene).

State	E [eV]	λ [nm]	*f*	Sym.	Major contributions
**1**	2.93	424	2.124	A	HOMO→LUMO (86%)
**2**	3.47	357	0.103	A	H-2→LUMO (19%), H-2→L+1 (14%), H-2→L+2 (12%), H-1→LUMO (11%), H-1→L+1 (27%)
**3**	3.47	357	0.677	A	H-2→LUMO (11%), H-2→L+1 (27%), H-1→LUMO (19%), H-1→L+1 (14%), H-1→L+2 (12%)
**4**	3.58	346	0.000	A	H-5→LUMO (12%), H-4→L+1 (12%), H-3→LUMO (15%), HOMO→L+1 (39%)
**5**	3.79	327	0.119	A	H-4→LUMO (25%), H-4→L+2 (11%), H-3→L+1 (34%), HOMO→L+2 (14%)
**6**	4.00	310	0	A	H-4→L+1 (28%), H-3→LUMO (20%), HOMO→L+1 (24%)
**7**	4.21	295	0.026	A	H-5→L+1 (17%), H-4→LUMO (11%), HOMO→L+2 (44%)
**8**	4.35	285	0.004	A	H-7→LUMO (27%), H-5→LUMO (37%)
**9**	4.36	284	0.089	A	H-12→LUMO (10%), H-6→LUMO (57%)
**10**	4.42	280	0.008	A	H-13→LUMO (33%), H-12→L+1 (18%), H-5→LUMO (11%)
**11**	4.44	279	0.157	A	H-13→L+1 (13%), H-12→LUMO (31%), H-6→LUMO (19%)
**12**	4.52	274	0.006	A	H-9→LUMO (10%), H-9→L+2 (12%), H-8→L+1 (18%)
**13**	4.52	274	0.028	A	H-9→L+1 (19%), H-8→LUMO (10%), H-8→L+2 (12%)
**14**	4.54	273	0.000	A	H-10→L+1 (16%), HOMO→L+3 (11%)
**15**	4.55	273	0.006	A	H-11→L+1 (17%), H-10→LUMO (10%), H-10→L+2 (11%)
**16**	4.58	271	0.001	A	H-5→L+2 (11%), HOMO→L+3 (25%)
**17**	4.65	267	0.003	A	H-7→LUMO (35%), H-5→LUMO (11%), HOMO→L+3 (10%)
**18**	4.68	265	0.123	A	H-2→LUMO (20%), H-1→LUMO (11%), HOMO→L+6 (12%)
**19**	4.68	265	0.206	A	H-2→LUMO (16%), H-1→LUMO (30%), H-1→L+2 (13%)
**20**	4.68	265	0.004	A	H-2→LUMO (10%), HOMO→L+4 (11%), HOMO→L+6 (25%), HOMO→L+26 (10%)
**21**	4.85	256	0.018	A	H-4→LUMO (15%), H-4→L+2 (10%)
**22**	4.86	255	0	A	H-3→LUMO (13%)
**23**	4.94	251	0.012	A	H-4→L+5 (15%), H-3→L+4 (15%), H-2→L+8 (11%), H-1→L+7 (11%)
**24**	4.94	251	0.063	A	H-4→L+4 (14%), H-3→L+5 (16%), H-2→L+7 (11%), H-1→L+8 (11%)
**25**	4.97	249	0.065	A	H-14→LUMO (13%), H-10→LUMO (10%), H-5→L+1 (11%), HOMO→L+2 (18%)

Compound 1

Calculated absorption spectrum

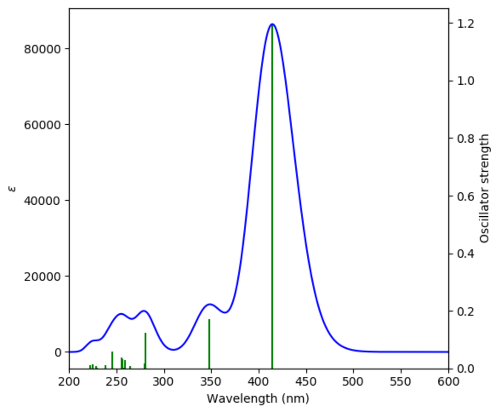

TD-DFT CAM-B3LYP/6-31G+(d,p), gas phase

Orbital	Energy [eV]		Sym.
	gas phase	toluene	
L+4	-6.65	-0.15	A
L+3	-6.74	-0.15	A
L+2	-6.75	-0.17	A
L+1	-7.50	-1.02	A
LUMO	-8.45	-2.04	A
HOMO	-13.65	-7.30	A
H-1	-14.29	-7.96	A
H-2	-14.39	-8.07	A
H-3	-15.17	-8.60	A
H-4	-15.17	-8.61	A

Orbitals relevant to the $S_1 \leftarrow S_0$ transition

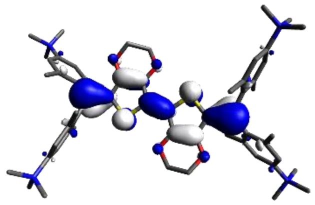

LUMO

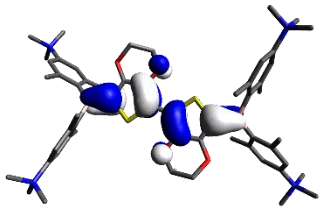

HOMO

other relevant orbitals

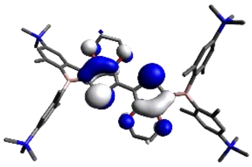

HOMO-1

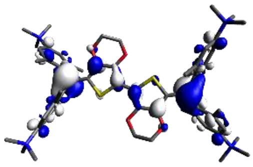

LUMO+1

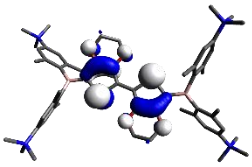

HOMO-2

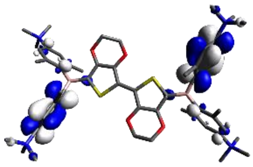

LUMO+2

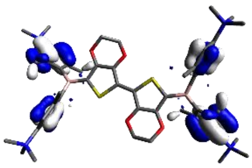

HOMO-3

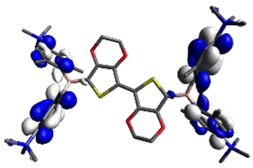

LUMO+3

Table S16. Lowest energy singlet electronic transition of **1** (TD-DFT CAM-B3LYP/6-31G+(d,p), gas phase).

State	E [eV]	λ [nm]	*f*	Sym.	Major contributions	Λ
**1**	2.99	415	1.193	A	HOMO→LUMO (94%)	0.71
**2**	3.56	348	0.172	A	H-1→LUMO (89%)	0.66
**3**	3.60	344	0.000	A	H-2→LUMO (87%), H-1→L+1 (10%)	0.64
**4**	4.04	307	0.000	A	HOMO→L+1 (79%)	0.56
**5**	4.42	280	0.125	A	H-6→LUMO (63%), H-5→L+1 (21%)	0.46
**6**	4.43	280	0.016	A	H-9→LUMO (11%), H-6→L+1 (24%), H-5→LUMO (55%)	0.50
**7**	4.64	267	0.000	A	H-9→LUMO (30%), H-1→L+1 (25%), HOMO→L+1 (10%)	0.51
**8**	4.68	265	0.009	A	H-4→LUMO (53%), H-3→L+1 (21%)	0.35
**9**	4.68	265	0.004	A	H-4→L+1 (23%), H-3→LUMO (55%)	0.36
**10**	4.78	259	0.029	A	H-10→LUMO (62%), H-9→L+1 (14%)	0.46
**11**	4.80	259	0.003	A	H-10→L+1 (13%), H-9→LUMO (23%), H-1→L+1 (33%)	0.53
**12**	4.83	256	0.002	A	H-8→LUMO (42%), H-7→L+1 (17%)	0.43
**13**	4.84	256	0.033	A	H-8→L+1 (20%), H-7→LUMO (44%)	0.47
**14**	4.85	256	0.036	A	H-2→L+1 (69%)	0.46
**15**	5.02	247	0.000	A	H-11→LUMO (69%), H-1→L+1 (13%)	0.69
**16**	5.06	245	0.059	A	HOMO→L+2 (29%), HOMO→L+4 (47%)	0.44
**17**	5.19	239	0.010	A	HOMO→L+2 (53%), HOMO→L+4 (24%)	0.35
**18**	5.19	239	0.003	A	HOMO→L+3 (80%)	0.24
**19**	5.39	230	0.001	A	H-8→L+3 (11%), H-7→LUMO (11%), H-3→LUMO (13%)	0.53
**20**	5.39	230	0.003	A	H-8→LUMO (15%), H-8→L+2 (11%), H-4→LUMO (12%)	0.50
**21**	5.43	228	0.007	A	H-8→LUMO (16%), H-4→LUMO (12%)	0.46
**22**	5.43	228	0.000	A	H-7→LUMO (15%), H-3→LUMO (11%)	0.49
**23**	5.51	225	0.014	A	H-2→L+3 (11%), H-1→L+2 (19%), HOMO→L+6 (16%), HOMO→L+14 (17%)	0.22
**24**	5.51	225	0.004	A	H-2→L+2 (13%), H-1→L+3 (16%), HOMO→L+5 (16%), HOMO→L+7 (15%), HOMO→L+11 (14%)	0.28
**25**	5.58	222	0.012	A	H-2→L+3 (16%), H-1→L+2 (22%), HOMO→L+6 (19%)	0.23

Table S17. Lowest energy singlet electronic transition of **1** (TD-DFT CAM-B3LYP/6-31G+(d,p), MeCN).

State	E [eV]	λ [nm]	*f*	Symmetry	Major contributions
**1**	2.97	418	1.272	A	HOMO→LUMO (95%)
**2**	3.61	344	0.219	A	H-1→LUMO (90%)
**3**	3.66	339	0.000	A	H-2→LUMO (88%)
**4**	4.07	305	0.000	A	HOMO→L+1 (76%)
**5**	4.33	286	0.148	A	H-8→LUMO (10%), H-6→LUMO (56%), H-5→L+1 (20%)
**6**	4.34	286	0.019	A	H-6→L+1 (21%), H-5→LUMO (53%)
**7**	4.58	271	0.010	A	H-4→LUMO (58%), H-3→L+1 (20%)
**8**	4.58	271	0.002	A	H-4→L+1 (20%), H-3→LUMO (54%)
**9**	4.62	268	0.000	A	H-10→L+1 (13%), H-9→LUMO (48%), HOMO→L+1 (11%)
**10**	4.67	266	0.024	A	H-10→LUMO (64%), H-9→L+1 (16%)
**11**	4.73	262	0.000	A	H-8→LUMO (47%), H-7→L+1 (18%)
**12**	4.73	262	0.050	A	H-8→L+1 (17%), H-7→LUMO (46%)
**13**	4.83	257	0.000	A	H-11→LUMO (26%), H-1→L+1 (46%)
**14**	4.96	250	0.039	A	H-2→L+1 (74%)
**15**	5.06	245	0.000	A	H-11→LUMO (58%), H-1→L+1 (27%)
**16**	5.15	241	0.085	A	HOMO→L+2 (53%), HOMO→L+4 (18%)
**17**	5.33	233	0.001	A	H-4→LUMO (11%)
**18**	5.33	233	0.001	A	H-3→LUMO (11%)
**19**	5.34	232	0.019	A	HOMO→L+2 (19%), HOMO→L+4 (34%)
**20**	5.37	231	0.002	A	HOMO→L+3 (60%)
**21**	5.38	230	0.007	A	H-8→LUMO (13%), H-4→LUMO (10%), H-4→L+4 (12%)
**22**	5.39	230	0.004	A	H-7→LUMO (12%), H-3→L+4 (10%), HOMO→L+3 (11%)
**23**	5.43	228	0.032	A	HOMO→L+4 (14%), HOMO→L+7 (32%), HOMO→L+13 (18%)
**24**	5.59	222	0.003	A	H-13→L+1 (11%), H-12→LUMO (59%)
**25**	5.61	221	0.002	A	HOMO→L+8 (20%), HOMO→L+11 (13%), HOMO→L+14 (12%)

Compound 2

Calculated absorption spectrum

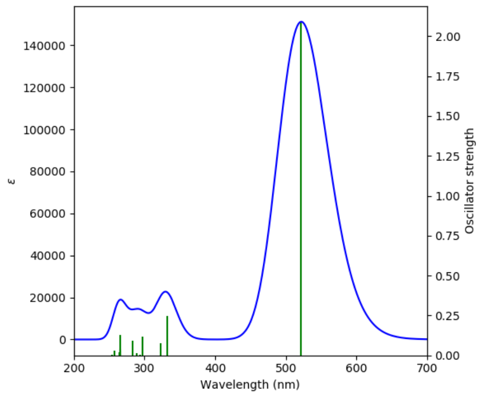

TD-DFT CAM-B3LYP/6-31G+(d,p), gas phase

Orbital	Energy [eV] gas phase	Energy [eV] toluene	Sym.
L+4	-6.07	-0.16	A
L+3	-6.07	-0.16	A
L+2	-6.22	-0.65	A
L+1	-7.00	-1.44	A
LUMO	-7.45	-2.04	A
HOMO	-11.69	-6.48	A
H-1	-12.91	-7.65	A
H-2	-13.07	-7.95	A
H-3	-13.37	-8.25	A
H-4	-13.95	-8.58	A

Orbitals relevant to the $S_1 \leftarrow S_0$ transition

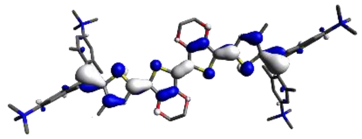

LUMO

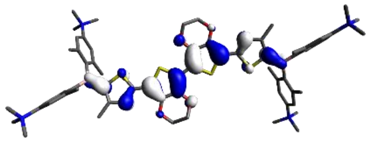

HOMO

other relevant orbitals

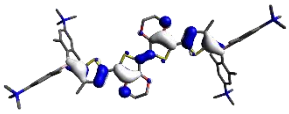

HOMO-1

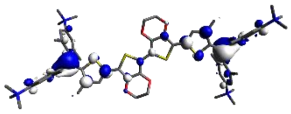

LUMO+1

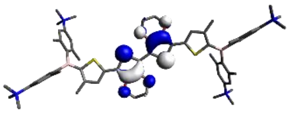

HOMO-2

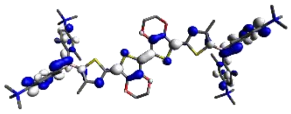

LUMO+2

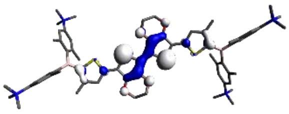

HOMO-3

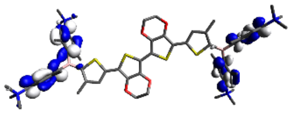

LUMO+3

Table S18. Lowest energy singlet electronic transition of **2** (TD-DFT CAM-B3LYP/6-31G+(d,p), gas phase).

State	E [eV]	λ [nm]	*f*	Sym.	Major contributions	Λ
**1**	2.38	522	2.087	A	HOMO→LUMO (88%)	0.67
**2**	3.05	406	0	A	H-1→LUMO (13%), HOMO→L+1 (77%)	0.56
**3**	3.70	335	0	A	H-1→LUMO (64%)	0.67
**4**	3.73	332	0.248	A	H-2→LUMO (71%)	0.53
**5**	3.84	323	0.074	A	H-2→LUMO (10%), HOMO→L+2 (63%)	0.60
**6**	3.97	312	0.000	A	H-3→LUMO (63%)	0.62
**7**	4.17	298	0.120	A	H-5→L+1 (30%), H-4→LUMO (57%)	0.54
**8**	4.22	294	0.003	A	H-5→LUMO (53%), H-4→L+1 (22%)	0.54
**9**	4.28	289	0.015	A	HOMO→L+4 (72%)	0.18
**10**	4.29	289	0.004	A	HOMO→L+3 (74%)	0.17
**11**	4.31	288	0.000	A	H-1→L+2 (11%), HOMO→L+5 (57%)	0.45
**12**	4.38	283	0.090	A	H-6→LUMO (21%), H-1→L+1 (33%), HOMO→L+2 (12%)	0.61
**13**	4.63	268	0.001	A	H-2→L+1 (74%)	0.37
**14**	4.66	266	0.003	A	H-9→LUMO (10%), HOMO→L+7 (54%)	0.26
**15**	4.66	266	0.030	A	H-10→LUMO (14%), H-9→L+1 (12%), HOMO→L+6 (47%)	0.26
**16**	4.67	266	0.042	A	H-10→L+1 (29%), H-9→LUMO (33%), HOMO→L+7 (16%)	0.43
**17**	4.67	266	0.127	A	H-10→LUMO (29%), H-9→L+1 (26%), HOMO→L+6 (24%)	0.37
**18**	4.69	264	0.021	A	H-6→LUMO (12%), H-3→L+1 (38%), H-1→L+1 (26%)	0.55
**19**	4.77	260	0.000	A	HOMO→L+7 (11%), HOMO→L+32 (36%)	0.33
**20**	4.79	259	0.000	A	HOMO→L+34 (52%)	0.36
**21**	4.83	257	0.030	A	HOMO→L+8 (48%), HOMO→L+14 (10%)	0.34
**22**	4.86	255	0.000	A	H-8→L+1 (19%), H-7→LUMO (19%)	0.40
**23**	4.89	253	0.005	A	H-8→LUMO (18%), H-7→L+1 (17%), HOMO→L+8 (10%)	0.40
**24**	4.89	253	0.001	A	H-13→LUMO (16%), HOMO→L+5 (17%), HOMO→L+9 (15%)	0.49
**25**	4.96	250	0.001	A	H-12→LUMO (20%), H-11→L+1 (17%)	0.50

Table S19. Lowest energy singlet electronic transition of **2** (TD-DFT CAM-B3LYP/6-31G+(d,p), MeCN).

State	E [eV]	λ [nm]	*f*	Sym.	Major contributions
**1**	2.43	510	2.127	A	HOMO→LUMO (90%)
**2**	3.18	390	0	A	H-1→LUMO (21%), HOMO→L+1 (69%)
**3**	3.84	323	0	A	H-1→LUMO (61%), HOMO→L+1 (16%)
**4**	3.89	319	0.436	A	H-2→LUMO (66%), H-1→L+1 (11%), HOMO→L+2 (12%)
**5**	4.01	309	0.049	A	H-2→LUMO (18%), HOMO→L+2 (48%)
**6**	4.12	301	0.001	A	H-3→LUMO (65%)
**7**	4.27	290	0.178	A	H-7→L+1 (14%), H-6→LUMO (11%), H-4→LUMO (42%)
**8**	4.35	285	0.003	A	H-7→LUMO (32%), H-3→LUMO (10%)
**9**	4.42	280	0.041	A	H-9→LUMO (36%), H-8→L+1 (29%)
**10**	4.42	280	0.175	A	H-9→L+1 (28%), H-8→LUMO (33%)
**11**	4.50	275	0.005	A	H-1→L+2 (12%), HOMO→L+3 (11%), HOMO→L+5 (30%)
**12**	4.57	271	0.044	A	H-12→LUMO (28%), H-1→L+1 (13%), HOMO→L+2 (22%)
**13**	4.61	269	0.004	A	HOMO→L+4 (20%), HOMO→L+20 (39%)
**14**	4.61	269	0.001	A	HOMO→L+3 (11%), HOMO→L+5 (12%), HOMO→L+19 (21%)
**15**	4.66	266	0.028	A	H-6→LUMO (30%), H-5→L+1 (27%)
**16**	4.66	266	0.000	A	H-6→L+1 (21%), H-5→LUMO (29%)
**17**	4.74	261	0.004	A	H-13→LUMO (39%), H-12→L+1 (15%)
**18**	4.78	260	0.001	A	H-11→L+1 (18%), H-10→LUMO (25%)
**19**	4.78	259	0.054	A	H-11→LUMO (28%), H-10→L+1 (20%)
**20**	4.80	258	0.003	A	H-14→LUMO (12%), H-13→L+1 (18%), H-12→LUMO (13%), H-1→L+1 (20%)
**21**	4.85	256	0.002	A	HOMO→L+9 (27%), HOMO→L+12 (31%)
**22**	4.95	250	0.000	A	H-2→L+1 (75%)
**23**	4.98	249	0.012	A	H-14→LUMO (24%), H-3→L+1 (32%), H-1→L+1 (18%)
**24**	5.05	246	0.010	A	HOMO→L+3 (44%), HOMO→L+17 (12%)
**25**	5.07	245	0.015	A	HOMO→L+4 (56%), HOMO→L+20 (14%)

Compound 3

Calculated absorption spectrum

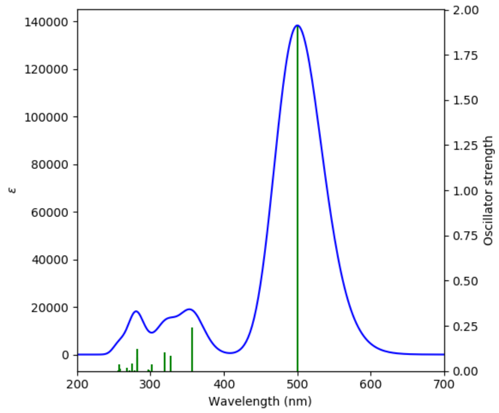

TD-DFT CAM-B3LYP/6-31G+(d,p), gas phase

Orbital	Energy [eV] gas phase	Energy [eV] toluene	Sym.
L+4	-6.01	-0.16	A
L+3	-6.01	-0.16	A
L+2	-6.09	-0.83	A
L+1	-7.01	-1.44	A
LUMO	-7.16	-1.77	A
HOMO	-11.29	-6.45	A
H-1	-12.57	-7.70	A
H-2	-12.59	-7.84	A
H-3	-12.93	-8.13	A
H-4	-13.47	-8.35	A

Orbitals relevant to the $S_1 \leftarrow S_0$ transition

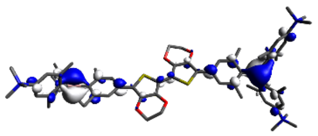

LUMO

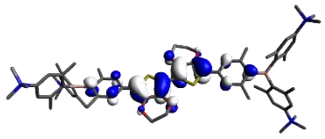

HOMO

other relevant orbitals

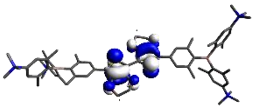

HOMO-1

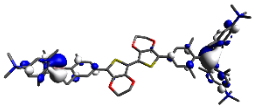

LUMO+1

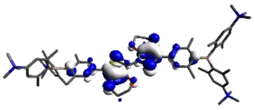

HOMO-2

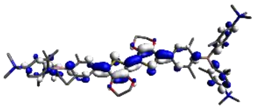

LUMO+2

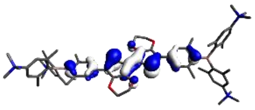

HOMO-3

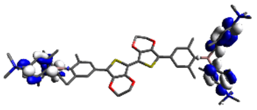

LUMO+3

Table S20. Lowest energy singlet electronic transition of **3** (TD-DFT CAM-B3LYP/6-31G+(d,p), gas phase).

State	E [eV]	λ [nm]	*f*	Symmetry	Major contributions	Λ
**1**	2.48	500	1.908	A	HOMO→LUMO (77%), HOMO→L+2 (11%)	0.47
**2**	2.88	431	0	A	HOMO→L+1 (80%)	0.38
**3**	3.48	356	0.242	A	HOMO→L+2 (70%)	0.60
**4**	3.77	329	0.000	A	H-2→LUMO (51%), H-1→L+1 (12%)	0.44
**5**	3.79	327	0.083	A	H-5→L+1 (10%), H-4→LUMO (12%), H-1→LUMO (46%), H-1→L+2 (11%)	0.37
**6**	3.85	322	0.000	A	H-5→LUMO (42%), H-4→L+1 (34%)	0.36
**7**	3.88	320	0.105	A	H-5→L+1 (27%), H-4→LUMO (37%), H-1→LUMO (13%)	0.36
**8**	4.05	306	0.000	A	H-6→L+1 (10%), H-3→LUMO (49%)	0.51
**9**	4.11	302	0.035	A	H-6→LUMO (22%), H-3→L+1 (22%), H-2→L+1 (12%), HOMO→LUMO (11%)	0.47
**10**	4.15	299	0.001	A	HOMO→L+3 (14%), HOMO→L+5 (23%), HOMO→L+7 (17%), HOMO→L+11 (18%)	0.31
**11**	4.17	298	0.002	A	HOMO→L+4 (87%)	0.15
**12**	4.17	297	0.006	A	HOMO→L+3 (73%)	0.15
**13**	4.40	282	0.072	A	H-9→L+1 (40%), H-8→LUMO (44%)	0.61
**14**	4.40	282	0.124	A	H-9→LUMO (42%), H-8→L+1 (42%)	0.62
**15**	4.45	279	0.002	A	H-2→L+2 (10%), H-1→L+1 (74%)	0.28
**16**	4.52	275	0.042	A	H-3→L+1 (21%), H-2→L+1 (51%), H-1→L+2 (11%)	0.39
**17**	4.56	272	0.001	A	HOMO→L+6 (88%)	0.07
**18**	4.56	272	0.001	A	HOMO→L+5 (38%), HOMO→L+7 (50%)	0.24
**19**	4.63	268	0.017	A	HOMO→L+8 (73%)	0.31
**20**	4.68	265	0.000	A	H-7→LUMO (13%), H-3→L+2 (11%), HOMO→L+5 (14%), HOMO→L+7 (11%)	0.46
**21**	4.73	262	0	A	H-10→L+1 (10%), HOMO→L+11 (36%)	0.45
**22**	4.79	259	0.015	A	H-11→L+1 (25%), H-10→LUMO (30%)	0.51
**23**	4.82	257	0.036	A	HOMO→L+30 (19%)	0.37
**24**	4.82	257	0.010	A	HOMO→L+29 (14%)	0.43
**25**	4.83	257	0.001	A	H-11→LUMO (18%), H-10→L+1 (15%)	0.49

Table S21. Lowest energy singlet electronic transition of **3** (TD-DFT CAM-B3LYP/6-31G+(d,p), MeCN).

State	E [eV]	λ [nm]	*f*	Sym.	Major contributions
**1**	2.70	459	2.162	A	HOMO→LUMO (82%)
**2**	3.33	373	0	A	H-1→LUMO (16%), HOMO→L+1 (67%)
**3**	3.80	326	0.065	A	H-1→L+1 (15%), HOMO→L+2 (56%)
**4**	4.06	305	0.002	A	H-5→LUMO (19%), H-4→L+1 (15%), H-3→LUMO (11%), H-1→LUMO (26%)
**5**	4.06	305	0.026	A	H-5→L+1 (21%), H-4→LUMO (33%), H-2→LUMO (22%)
**6**	4.11	302	0.006	A	H-5→LUMO (23%), H-4→L+1 (17%), H-1→LUMO (28%)
**7**	4.16	298	0.163	A	H-4→LUMO (14%), H-2→LUMO (45%), H-2→L+2 (12%)
**8**	4.26	291	0.144	A	H-8→LUMO (12%), H-8→L+1 (21%), H-7→LUMO (20%), H-7→L+1 (14%)
**9**	4.26	291	0.164	A	H-8→LUMO (18%), H-8→L+1 (13%), H-7→LUMO (11%), H-7→L+1 (20%)
**10**	4.34	286	0.001	A	H-6→L+1 (12%), H-3→LUMO (37%)
**11**	4.44	279	0	A	H-3→LUMO (13%), H-1→L+2 (11%), HOMO→L+5 (37%)
**12**	4.46	278	0.035	A	H-13→L+1 (14%), H-6→LUMO (20%), HOMO→L+2 (18%)
**13**	4.62	269	0	A	H-10→L+1 (18%), H-9→LUMO (31%), H-9→L+2 (11%), H-6→L+1 (16%)
**14**	4.62	268	0.069	A	H-10→LUMO (23%), H-9→L+1 (34%)
**15**	4.67	266	0.022	A	H-12→L+1 (10%), H-11→LUMO (26%), H-11→L+1 (21%)
**16**	4.67	266	0.060	A	H-12→LUMO (26%), H-12→L+1 (21%), H-11→L+1 (10%)
**17**	4.71	263	0.000	A	H-14→L+1 (16%), H-13→LUMO (32%), H-10→L+1 (10%), HOMO→L+5 (13%)
**18**	4.77	260	0.015	A	HOMO→L+9 (16%), HOMO→L+12 (48%), HOMO→L+22 (11%)
**19**	4.85	256	0.001	A	H-14→LUMO (26%), H-13→L+1 (22%), H-1→L+1 (24%)
**20**	4.95	251	0.003	A	HOMO→L+3 (27%), HOMO→L+17 (14%), HOMO→L+18 (13%)
**21**	4.96	250	0.045	A	HOMO→L+4 (33%), HOMO→L+15 (11%)
**22**	5.06	245	0.001	A	H-2→L+1 (56%), H-1→L+2 (14%)
**23**	5.11	242	0.009	A	HOMO→L+10 (14%), HOMO→L+16 (29%)
**24**	5.13	242	0.004	A	H-3→L+1 (26%), H-1→L+1 (17%)
**25**	5.15	241	0	A	H-15→LUMO (13%), H-14→L+1 (16%), H-3→L+2 (11%), H-2→L+1 (14%)

Theoretical Studies: Cartesian Coordinates

Compound 1N

DFT B3LYP/6-31G+(d,p), gas phase, S_0

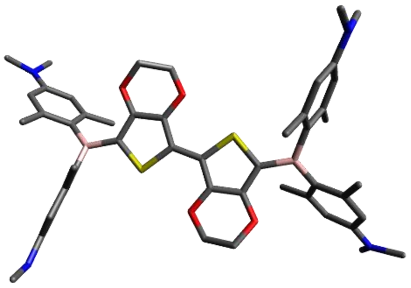

Point group: C_1

Total energy: -2,124,876.35 kcal mol^{-1}

Dipole moment: 0.62 D

Immaginary frequencies: 0

C	3.24722200	0.31432500	-0.04771500
B	4.74683100	-0.06772800	0.00386200
C	5.80737800	0.94329600	0.59545600
C	5.15935600	-1.50611500	-0.52428800
C	6.98341700	1.29213600	-0.12512100
C	7.92349600	2.17925900	0.40457100
C	7.78148400	2.73569600	1.69207200
C	6.61988700	2.38693300	2.40970100
C	5.65102200	1.53032700	1.88082900
C	5.84063200	-2.43331400	0.31292700
C	6.19398600	-3.70210100	-0.14963400
C	5.91629700	-4.12079900	-1.46826200
C	5.27660200	-3.18964000	-2.30883400
C	4.88624700	-1.92630000	-1.85275700
C	4.44867100	1.25104700	2.76500500
C	7.25715800	0.75854500	-1.51883100
C	6.18438600	-2.10068000	1.75263400
C	4.19825500	-1.02952800	-2.86819000
N	8.75351200	3.57231800	2.23799500
N	6.25138200	-5.40011900	-1.91230100
C	7.17258500	-6.20284300	-1.12217100
C	9.80398700	4.09295000	1.37733100
C	8.43784600	4.33628800	3.43477800
C	2.60715900	1.54736300	-0.09471000
C	1.19027800	1.48063600	-0.15083400
C	0.68551200	0.18666700	-0.14037300
S	2.01986000	-0.94923400	-0.08534200
O	3.26320700	2.74725800	-0.09222000
C	2.44663200	3.84611300	-0.51892900
C	1.08143200	3.79588600	0.14713600
O	0.39240500	2.59172200	-0.21874400
C	-0.69796500	-0.21343500	-0.14544200
S	-2.03371000	0.92172100	-0.10679900
C	-3.26030200	-0.34287700	-0.07366400
C	-2.61888900	-1.57558700	-0.10992800
C	-1.20156500	-1.50781200	-0.15462700
O	-3.27352900	-2.77587400	-0.10751100
C	-2.45281600	-3.87596300	-0.52352800
C	-1.09303900	-3.82219200	0.15311300
O	-0.40231900	-2.61815800	-0.21106200
B	-4.76094000	0.03703800	-0.03620300
C	-5.82227600	-0.97541600	0.55114200
C	-5.17370300	1.47114100	-0.57578800
C	-6.99817900	-1.32252000	-0.17146600
C	-7.93464700	-2.21651600	0.35261100
C	-7.78040500	-2.79775700	1.62770300
C	-6.63672300	-2.42386300	2.36107500
C	-5.67049900	-1.56064600	1.83777700
C	-5.85099900	2.40565200	0.25560600
C	-6.20808200	3.66990600	-0.21709000
C	-5.95531800	4.06810600	-1.54699100
C	-5.28900200	3.14163200	-2.37216900
C	-4.89636900	1.88289000	-1.90609700
C	-4.48054600	-1.26629500	2.73376600
C	-7.28138600	-0.77268900	-1.55704300
C	-6.17746800	2.08853800	1.70274200
C	-4.19246400	0.98419200	-2.90888800
N	-8.70971100	-3.70570000	2.13344700
N	-6.36653200	5.31127200	-2.02706800
C	-6.82064200	6.31951500	-1.08228400
C	-5.84102400	5.78482000	-3.29798100
C	-8.64431200	-4.07487300	3.53881800
C	6.18028600	-5.69259500	-3.33524300
C	-10.00284100	-3.83199300	1.47856600
H	8.78383300	2.42979300	-0.20430000
H	6.45814900	2.78165400	3.40591500
H	6.69659000	-4.37097900	0.53868300
H	5.07406500	-3.43854400	-3.34408100
H	3.60216100	1.88887400	2.48847700
H	4.10361700	0.21678200	2.69726800
H	4.68696700	1.45602100	3.81359800
H	6.44859800	1.00669200	-2.21488600
H	8.17980700	1.18640200	-1.92244000
H	7.35833600	-0.33028900	-1.52247800
H	5.28887800	-1.85884600	2.33625800
H	6.67394000	-2.94861800	2.24099500
H	6.85190200	-1.23699800	1.82005200
H	4.59735400	-1.20921000	-3.87223200
H	3.12052000	-1.22762800	-2.90078100
H	4.31906100	0.03205400	-2.64412800
H	7.29410900	-7.17697500	-1.59970500
H	8.16782700	-5.73908500	-1.02048500
H	6.77562300	-6.37874600	-0.11733300
H	10.49487300	4.68675600	1.97873200
H	10.37912000	3.27787900	0.92641500
H	9.41700100	4.72906600	0.56379200
H	9.31817300	4.90784500	3.73456400
H	8.17965600	3.67271400	4.26653500
H	7.60121200	5.03916300	3.28648600
H	2.98841800	4.75171400	-0.23607600
H	2.33676100	3.81724200	-1.61150100
H	0.44798800	4.62075900	-0.18671400
H	1.18617400	3.83749100	1.23950400
H	-2.99637700	-4.78069300	-0.24143400
H	-2.33451600	-3.85116400	-1.61528900
H	-0.45588100	-4.64722200	-0.17307300
H	-1.20621700	-3.86029900	1.24472600
H	-8.80104800	-2.45558000	-0.25227700
H	-6.48479400	-2.80625200	3.36354300
H	-6.69683200	4.34933700	0.47086500
H	-5.07125900	3.38979500	-3.40459400
H	-3.62949300	-1.90642500	2.47691000
H	-4.13634400	-0.23249400	2.65732100
H	-4.73228800	-1.45864700	3.78170000
H	-7.39217800	0.31507800	-1.54682900
H	-6.47335000	-1.00476800	-2.25907800
H	-8.20150600	-1.20382500	-1.96288400
H	-5.27385400	1.86470900	2.28124000
H	-6.67296100	2.93669400	2.18472800
H	-6.83416700	1.21793700	1.78676700

H	-4.57486000	1.16188600	-3.91964400
H	-3.11402900	1.18058300	-2.92395900
H	-4.31816300	-0.07686500	-2.68449000
H	-7.14044900	7.20539300	-1.63420900
H	-7.68324000	5.96019800	-0.51200700
H	-6.03838700	6.62083200	-0.36554300
H	-6.28571500	6.75491700	-3.52763400
H	-6.10802100	5.10186500	-4.11109800
H	-4.74405600	5.89741900	-3.29331300
H	-9.41019300	-4.82483800	3.74544200
H	-8.80470500	-3.21928200	4.21599600
H	-7.67445000	-4.52150100	3.78005100
H	6.87278100	-5.07900300	-3.93576900
H	6.42620800	-6.74418300	-3.49488300
H	5.16669600	-5.53139900	-3.71580900
H	-9.88426900	-4.14045200	0.43493200
H	-10.58294700	-4.60642500	1.98393100
H	-10.58489200	-2.89569700	1.49434800

Compound 1

DFT B3LYP/6-31G+(d,p), gas phase, S_0

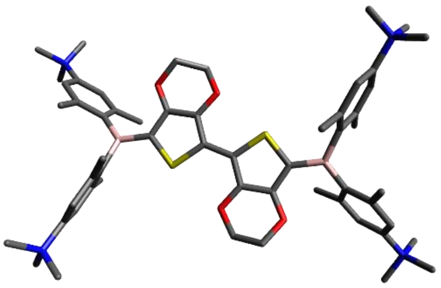

Point group: C_1

Total energy: -2,224,338.35 kcal mol^{-1}

Dipole moment: 0.31 D

Immaginary frequencies: 0

C	-3.25662900	0.31927900	0.01926600
B	-4.74732800	-0.02275500	0.01014200
C	-5.83375800	1.05127300	-0.47978200
C	-5.23674400	-1.47851900	0.48514700
C	-6.87627500	1.49509900	0.37229300
C	-7.85920900	2.38516200	-0.09381800
C	-7.82983700	2.83697700	-1.40725300
C	-6.80613200	2.42850400	-2.25820500
C	-5.80248600	1.56007100	-1.80699500
C	-5.90794900	-2.36081200	-0.40223200
C	-6.40387300	-3.59514000	0.04706900
C	-6.24851100	-3.97111900	1.37726900
C	-5.57233300	-3.13561000	2.25965200
C	-5.05430000	-1.90423500	1.82821700
C	-4.70290100	1.22379300	-2.79361100
C	-6.96725500	1.07143800	1.82256700
C	-6.09133500	-2.03553500	-1.86855000
C	-4.32438000	-1.08400800	2.87436800
N	-8.89612300	3.76725600	-1.94893600
N	-6.81456500	-5.27555400	1.90171800
C	-7.51416700	-6.08647500	0.83406400
C	-5.69815100	-6.13492400	2.46611700
C	-9.94027100	4.13162600	-0.91750800
C	-8.25997100	5.06017100	-2.42691500
C	-2.61214700	1.56064100	-0.00325900
C	-1.19584300	1.49394300	0.04843000
C	-0.69364900	0.19638400	0.09461600
S	-2.02460300	-0.94621800	0.10602900
O	-3.26507100	2.74837600	-0.05812300
C	-2.44418500	3.88510600	0.29399300
C	-1.08514000	3.79328300	-0.37483200
O	-0.39885600	2.59726500	0.05046200
C	0.69369000	-0.19641500	0.09461800
S	2.02466700	0.94618500	0.10592900
C	3.25666300	-0.31931300	0.01910300
C	2.61218500	-1.56067700	-0.00336400
C	1.19587400	-1.49397200	0.04840000
O	3.26509300	-2.74841900	-0.05822700
C	2.44421300	-3.88513800	0.29392400
C	1.08514700	-3.79331000	-0.37485600
O	0.39888400	-2.59728800	0.05046900
B	4.74736800	0.02275000	0.01000100
C	5.83383800	-1.05123000	-0.47992200
C	5.23671100	1.47850100	0.48508700
C	6.87645300	-1.49482700	0.37216300
C	7.85938500	-2.38494300	-0.09384900
C	7.82994600	-2.83699900	-1.40720000
C	6.80618400	-2.42870500	-2.25816700
C	5.80253800	-1.56022100	-1.80705300
C	5.90776300	2.36089600	-0.40231900
C	6.40360700	3.59525300	0.04698300
C	6.24834300	3.97116100	1.37721900
C	5.57233500	3.13554900	2.25962600
C	5.05438100	1.90413400	1.82819100
C	4.70292800	-1.22407600	-2.79368400
C	6.96766600	-1.07073100	1.82230100
C	6.09108400	2.03567400	-1.86865700
C	4.32482200	1.08376800	2.87449000
N	8.89619500	-3.76739800	-1.94874600
N	6.81426800	5.27565200	1.90167200
C	7.51370900	6.08671300	0.83401700
C	5.69777800	6.13484800	2.46617300
C	9.94042800	-4.13149200	-0.91730800
C	8.26001300	-5.06044100	-2.42634400
C	-7.83084900	-4.99027600	2.99449600
C	9.61634200	-3.10289000	-3.10945700
C	7.83065000	4.99046400	2.99438300
C	-9.61636500	3.10246100	-3.10942200
H	-8.62987900	2.69277800	0.60012200
H	-6.75693400	2.77140700	-3.28640800
H	-6.90910100	-4.22684700	-0.67109400
H	-5.43343200	-3.40654400	3.30141300
H	-3.85469200	1.90385300	-2.65842100
H	-4.32066300	0.20926000	-2.67687000
H	-5.05122900	1.33340000	-3.82423300
H	-6.07094000	1.36305300	2.37903500
H	-7.82177700	1.53707500	2.31903300
H	-7.07792400	-0.01206100	1.91878600
H	-5.12550200	-1.95711900	-2.37918700
H	-6.66494400	-2.81195100	-2.38009600
H	-6.61695900	-1.08725600	-2.00788500
H	-4.87781800	-1.07312500	3.81874700
H	-3.33778500	-1.51567500	3.07759700
H	-4.16268100	-0.05178500	2.56629500
H	-7.88468000	-6.99930500	1.29962000
H	-8.35061600	-5.51550800	0.43401600
H	-6.80545000	-6.33858600	0.04660100
H	-6.12851400	-7.07070600	2.82466700
H	-5.21745600	-5.61017900	3.28861900
H	-4.97739000	-6.32625300	1.67161200
H	-10.65940000	4.79750800	-1.39368500
H	-10.44680000	3.22905400	-0.57907200
H	-9.46363000	4.64442100	-0.08337500
H	-9.04700800	5.71702100	-2.79915200
H	-7.55234800	4.84247100	-3.22367500
H	-7.74546200	5.52141400	-1.58439400
H	-2.99044500	4.76611700	-0.04693800
H	-2.34061800	3.92222700	1.38474000
H	-0.44760600	4.62859600	-0.08093400

H	-1.18417400	3.77812100	-1.46685400
H	2.99046000	-4.76615600	-0.04701300
H	2.34067900	-3.92224900	1.38467500
H	0.44761300	-4.62861900	-0.08094700
H	1.18414700	-3.77813700	-1.46688100
H	8.63011700	-2.69240600	0.60009200
H	6.75692200	-2.77180500	-3.28630100
H	6.90869900	4.22703700	-0.67120800
H	5.43350500	3.40642000	3.30141500
H	3.85462900	-1.90397800	-2.65827400
H	4.32084500	-0.20945600	-2.67718300
H	5.05118200	-1.33399500	-3.82429700
H	7.07995200	0.01265300	1.91807200
H	6.07080600	-1.36074000	2.37870300
H	7.82138300	-1.53743100	2.31915500
H	5.12522900	1.95694900	-2.37920100
H	6.66439400	2.81226900	-2.38026500
H	6.61698800	1.08755200	-2.00803900
H	4.87960100	1.07113700	3.81807100
H	3.33911800	1.51657100	3.07960700
H	4.16128600	0.05210100	2.56554700
H	7.88415600	6.99955500	1.29960400
H	8.35019600	5.51586500	0.43387800
H	6.80491300	6.33879600	0.04661800
H	6.12803900	7.07066400	2.82475900
H	5.21718800	5.60999100	3.28866700
H	4.97695400	6.32613500	1.67171600
H	10.65953400	-4.79747800	-1.39337500
H	10.44696300	-3.22882500	-0.57913400
H	9.46386000	-4.64409000	-0.08301300
H	9.04702400	-5.71738200	-2.79847400
H	7.55232600	-4.84294600	-3.22310300
H	7.74557300	-5.52147000	-1.58366500
H	-8.62187200	-4.36976900	2.57436500
H	-8.23721800	-5.93916900	3.34659900
H	-7.34291200	-4.46837000	3.81461600
H	10.06435500	-2.17663100	-2.75083000
H	10.38700900	-3.78284500	-3.47446100
H	8.90365700	-2.88989600	-3.90300800
H	8.62173100	4.37007900	2.57418300
H	8.23691100	5.93939700	3.34650200
H	7.34282500	4.46846200	3.81450900
H	-10.06436100	2.17629900	-2.75052300
H	-10.38705000	3.78233100	-3.47454500
H	-8.90373600	2.88925300	-3.90296800

Compound 2N

DFT B3LYP/6-31G+(d,p), gas phase, S_0

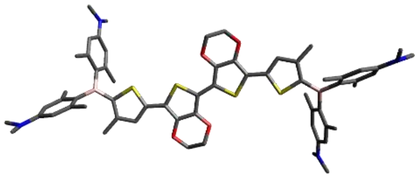

Point group: C_1

Total energy: -2,866,771.33 kcal mol^{-1}

Dipole moment: 0.51 D

Immaginary frequencies: 0

C	-6.96847200	0.91629700	0.03604600
B	-8.33289100	0.17553500	-0.00591200
C	-8.43770200	-1.24576500	0.68784400
C	-9.56040900	0.83118200	-0.75785400
C	-8.88138900	-2.39473200	-0.02473600
C	-8.96642100	-3.64332300	0.59440800
C	-8.64253000	-3.82586500	1.95522500
C	-8.24181700	-2.68107300	2.67220100
C	-8.11799600	-1.43008500	2.06098200
C	-10.82840100	0.98102300	-0.12995500
C	-11.90367200	1.57142900	-0.79842300
C	-11.79868500	2.02016900	-2.13128000
C	-10.55565000	1.83025800	-2.76970800
C	-9.45887800	1.27524300	-2.10568800
C	-7.66738500	-0.28592500	2.95321000
C	-9.24865900	-2.32891100	-1.49535200
C	-11.06964300	0.54031200	1.30191400
C	-8.17982300	1.13569600	-2.91377400
N	-8.70728200	-5.08092600	2.55755800
N	-12.86856200	2.62823700	-2.78221500
C	-14.19525000	2.55287500	-2.18982000
C	-9.39994500	-6.16057400	1.87107700
C	-6.61919700	2.26900100	0.01438200
C	-5.21949000	2.49771500	0.06218500
C	-4.45509100	1.33808200	0.10379200
S	-5.49914400	-0.05971100	0.12552300
C	-3.01753100	1.22596000	0.12138700
S	-2.20430900	-0.33474700	0.14035600
C	-0.60151600	0.39944300	0.15610800
C	-0.73237500	1.77762800	0.14729900
C	-2.07466800	2.24026800	0.13391700
C	0.60648600	-0.37574500	0.15860700
C	0.73730300	-1.75395300	0.16316000
C	2.07954000	-2.21676000	0.15141400
C	3.02240800	-1.20268100	0.12676300
S	2.20925500	0.35817800	0.13207400
C	4.45991700	-1.31511900	0.10713400
S	5.50423400	0.08265500	0.11246600
C	6.97318500	-0.89436800	0.02967900
C	6.62363400	-2.24712300	0.02215700
C	5.22405800	-2.47524800	0.07546400

O	0.33999300	2.62889100	0.16795000
C	-0.00449800	3.96568900	-0.22553700
C	-1.27396500	4.41327000	0.48070700
O	-2.38279700	3.57467000	0.11644000
O	-0.33505400	-2.60494100	0.19461200
C	0.00853600	-3.94552100	-0.18646100
C	1.27951200	-4.38619200	0.52141700
O	2.38759000	-3.55131900	0.14647200
B	8.33796200	-0.15484200	-0.02170300
C	8.44632600	1.27237600	0.65914200
C	9.56458600	-0.81610600	-0.77056900
C	8.89386200	2.41353700	-0.06386800
C	8.98196400	3.66751900	0.54369100
C	8.65800600	3.86370200	1.90260900
C	8.25369700	2.72682700	2.63000500
C	8.12639900	1.47055000	2.03038600
C	10.82953500	-0.97124800	-0.13870700
C	11.90563400	-1.56369900	-0.80436100
C	11.80949300	-1.99102500	-2.14483300
C	10.55960200	-1.82592400	-2.77682800
C	9.46240400	-1.26879300	-2.11558800
C	7.67165300	0.33668200	2.93366800
C	9.26229600	2.33351500	-1.53350100
C	11.06282200	-0.54267400	1.29809400
C	8.17970500	-1.14429900	-2.92037000
N	8.72618500	5.12410300	2.49315700
N	12.90139900	-2.53024500	-2.81922500
C	14.09237900	-2.89242600	-2.06679900
C	12.68904800	-3.19688500	-4.09477000
C	9.42245100	6.19527900	1.79704100
C	-12.79260600	2.85119500	-4.21762100
C	-8.60466700	-5.17661300	4.00525200
C	8.62254400	5.23377800	3.93977500
C	7.59234700	-3.40417800	-0.00455900
C	-7.58791200	3.42581900	-0.02119400
H	-9.29324700	-4.48518400	-0.00411500
H	-8.02012100	-2.75162800	3.73052900
H	-12.83934300	1.67529600	-0.26230300
H	-10.42952600	2.11434800	-3.80768000
H	-7.91249100	-0.49553400	3.99941600
H	-6.58353200	-0.13768800	2.89039500
H	-8.13166100	0.66587100	2.68491200
H	-8.40996700	-1.97625700	-2.10595300
H	-9.53163900	-3.31754800	-1.86897600
H	-10.08367300	-1.64585500	-1.67448300
H	-10.37936700	1.02609900	2.00021400
H	-12.08632400	0.79207100	1.61820000
H	-10.93333900	-0.53820100	1.42073800
H	-8.39474100	1.20277700	-3.98491500
H	-7.45967100	1.92395600	-2.67089800
H	-7.67191700	0.18478500	-2.73424400
H	-14.89561400	3.11031200	-2.81435400
H	-14.56314200	1.51814500	-2.09399100
H	-14.20660800	3.01098900	-1.19540900
H	-9.32339100	-7.06962100	2.47051200
H	-10.46791300	-5.94534900	1.70140300
H	-8.93713500	-6.36834500	0.90091100
H	-4.77498900	3.48502200	0.06356100
H	4.77940700	-3.46241500	0.08759400
H	0.84392800	4.59265000	0.05692600
H	-0.13796200	4.00190500	-1.31475900
H	-1.55191600	5.42661200	0.18220500
H	-1.13612700	4.37945500	1.56932600
H	-0.83929000	-4.56965000	0.10397400
H	0.13967700	-3.99246800	-1.27556200
H	1.55676200	-5.40246200	0.23235500
H	1.14401700	-4.34159700	1.60994400
H	9.31138500	4.50284600	-0.06249700
H	8.03171000	2.80785900	3.68752900
H	12.83506200	-1.68360900	-0.26080200
H	10.42964400	-2.12920000	-3.80894100
H	7.92320600	0.55284700	3.97702700
H	6.58640100	0.19616600	2.87715000
H	8.12749600	-0.62059200	2.67084700
H	8.42650400	1.96857200	-2.14072400
H	9.53917600	3.31971900	-1.91792900
H	10.10199100	1.65413600	-1.70445200

H	10.36539300	-1.03002600	1.98825500
H	12.07621700	-0.80083800	1.61973000
H	10.93065900	0.53572200	1.42309000
H	7.45933400	-1.92628900	-2.65898400
H	7.67411600	-0.18920400	-2.75650700
H	8.38940800	-1.23156300	-3.99101400
H	14.85392400	-3.25547500	-2.75926300
H	14.50782000	-2.02152200	-1.54936800
H	13.90293200	-3.67788200	-1.31660500
H	13.65156900	-3.53091600	-4.48612100
H	12.25745700	-2.50936300	-4.82974300
H	12.02420000	-4.07246100	-4.01418900
H	9.34823000	7.11008400	2.38794600
H	10.48985200	5.97530900	1.63004800
H	8.96082800	6.39536500	0.82470300
H	-12.71203300	1.91445700	-4.79357100
H	-13.69102200	3.37839900	-4.54359700
H	-11.93301300	3.48046800	-4.47006200
H	-9.42504200	-4.65839200	4.52938100
H	-8.62314600	-6.22911600	4.29426800
H	-7.65731600	-4.75613300	4.35738000
H	9.44090400	4.71810000	4.46953100
H	8.64390800	6.28890200	4.21886300
H	7.67363000	4.81945200	4.29498600
H	7.33366300	-4.13616400	0.76935600
H	7.55024700	-3.92750800	-0.96832900
H	8.62218600	-3.08343600	0.15013700
H	-8.61649600	3.10795900	0.14704100
H	-7.32231100	4.16899500	0.73955100
H	-7.55430400	3.93490200	-0.99287100

Compound 2

DFT B3LYP/6-31G+(d,p), gas phase, S_0

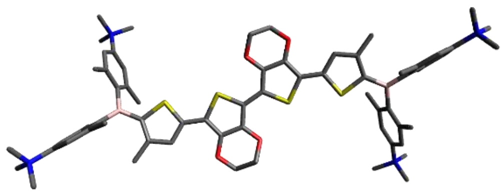

Point group: C_1

Total energy: -2966258.88 kcal mol^{-1}

Dipole moment: 0.16 D

Immaginary frequencies: 0

C	7.01040300	-0.83208000	-0.06783000
B	8.37768700	-0.16665900	-0.03514900
C	8.57097700	1.25789200	0.68797700
C	9.63951800	-0.88231800	-0.72687700
C	9.02339100	2.40691900	-0.01121100
C	9.24632700	3.62080400	0.65972000
C	9.03182100	3.71144800	2.03012700
C	8.57399000	2.60467600	2.73712900
C	8.32874400	1.38750800	2.08399600
C	10.78750400	-1.26411900	0.01164200
C	11.87909600	-1.88557800	-0.61967500
C	11.85320100	-2.12255600	-1.98849200
C	10.74032000	-1.74941700	-2.73782400
C	9.63287300	-1.14708000	-2.12481900
C	7.80529500	0.25817100	2.94949600
C	9.24989800	2.39792900	-1.50708500
C	10.87928300	-1.06709500	1.50954500
C	8.45446700	-0.81925900	-3.02077600
N	9.29545100	4.99652100	2.78958900
N	13.01716000	-2.78138900	-2.70073000
C	14.15230500	-3.13807100	-1.76858600
C	12.55333700	-4.06628000	-3.36358400
C	9.78528200	6.11580600	1.90042500
C	8.01800900	5.47096100	3.45666900
C	6.62708700	-2.17900600	-0.24668300
C	5.23662200	-2.38375100	-0.19873800
C	4.48362300	-1.22142000	-0.00851700
S	5.54760100	0.14803200	0.16970400
C	3.05104500	-1.12173800	0.02592800
S	2.19707400	0.41792500	0.07914000
C	0.61668300	-0.36613300	0.08815900
C	0.78227400	-1.74346700	0.05425300
C	2.13430900	-2.16876700	0.02070600
C	-0.61665800	0.36642100	0.08796900
C	-0.78225200	1.74372500	0.05296400
C	-2.13429200	2.16900800	0.01937500
C	-3.05103800	1.12198700	0.02562500
S	-2.19704600	-0.41763800	0.07996200
C	-4.48362400	1.22161100	-0.00861700
S	-5.54749600	-0.14795800	0.16934700
C	-7.01040600	0.83216900	-0.06763000
C	-6.62718700	2.17915300	-0.24614300
C	-5.23672000	2.38396000	-0.19838400
O	-0.26317000	-2.61753900	0.05542700
C	0.11202400	-3.94127300	-0.37826900
C	1.39288700	-4.37690300	0.31040600
O	2.47816800	-3.48264100	-0.02189700
O	0.26319600	2.61779800	0.05315900
C	-0.11208900	3.94111000	-0.38173900
C	-1.39276200	4.37742500	0.30685300
O	-2.47814900	3.48284300	-0.02425800
B	-8.37762800	0.16662900	-0.03507900
C	-8.57081500	-1.25820700	0.68748800
C	-9.63953300	0.88251500	-0.72644000
C	-9.02330200	-2.40694300	-0.01212100
C	-9.24622800	-3.62109300	0.65833800
C	-9.03160800	-3.71229300	2.02868800
C	-8.57366300	-2.60582100	2.73609200
C	-8.32843600	-1.38839700	2.08343300
C	-10.78746400	1.26396700	0.01236000
C	-11.87915100	1.88562000	-0.61859500
C	-11.85341700	2.12312800	-1.98732700
C	-10.74060000	1.75034300	-2.73692300
C	-9.63305600	1.14782200	-2.12426800
C	-7.80484500	-0.25946100	2.94937300
C	-9.24989700	-2.39734400	-1.50798000
C	-10.87908000	1.06629300	1.51018600
C	-8.45475100	0.82041200	-3.02051100
N	-9.29526400	-4.99764400	2.78767000
N	-13.01749900	2.78215900	-2.69918300
C	-14.15257500	3.13838300	-1.76678100
C	-12.55383900	4.06734900	-3.36157200
C	-9.78516000	-6.11657400	1.89809300
C	-8.01782900	-5.47239600	3.45454000
C	13.57303700	-1.84250300	-3.75629000
C	10.35789000	4.75858200	3.84777000
C	-13.57341600	1.84365200	-3.75505900
C	-10.35766600	-4.76005700	3.84596700
C	-7.56364800	3.34868800	-0.43068400
C	7.56344300	-3.34855000	-0.43171100
H	9.59059500	4.46399600	0.07601500
H	8.39757400	2.65015500	3.80695100
H	12.72465400	-2.16525700	-0.00560100
H	10.69845800	-1.91920300	-3.80868300
H	8.23890000	0.30151900	3.95311000
H	6.71709900	0.33188000	3.05569500
H	8.01179100	-0.72647200	2.53165400
H	8.30783000	2.24971100	-2.04584100
H	9.67412000	3.34426500	-1.85137300
H	9.93166700	1.59822000	-1.80692400
H	10.11664700	-1.65164500	2.03520100
H	11.85158800	-1.38445400	1.89391700
H	10.73616900	-0.01923100	1.78593100
H	8.77461400	-0.70009300	-4.05962500
H	7.70927700	-1.62176400	-2.99721300
H	7.93745200	0.09252900	-2.72144200
H	14.93682900	-3.60473800	-2.36360800
H	14.53693200	-2.23253200	-1.30184400
H	13.79699800	-3.84035100	-1.01596900
H	13.40837100	-4.53057800	-3.85643300
H	11.78248200	-3.83764600	-4.09598200
H	12.15277400	-4.72470800	-2.59330400
H	9.94604100	6.99258500	2.52716400
H	10.72418400	5.82323500	1.43293200
H	9.03122200	6.33680700	1.14651600
H	8.22706800	6.40214200	3.98476400
H	7.67472600	4.71510200	4.15929000
H	7.26649200	5.62954700	2.68376200
H	4.77482800	-3.35612800	-0.30353900
H	-4.77501000	3.35640300	-0.30290400
H	-0.71875600	-4.59451700	-0.10649100
H	0.23474500	-3.94005100	-1.46802900
H	1.70607700	-5.36525100	-0.03022200
H	1.26538600	-4.38870500	1.39919400
H	0.71876800	4.59461100	-0.11082000
H	-0.23509700	3.93882300	-1.47146400
H	-1.70604200	5.36543600	-0.03466800
H	-1.26496200	4.39030200	1.39559300

H	-9.59055700	-4.46403800	0.07431300
H	-8.39714000	-2.65174600	3.80587900
H	-12.72465100	2.16503500	-0.00432000
H	-10.69886400	1.92053300	-3.80772400
H	-8.23889900	-0.30281000	3.95279500
H	-6.71673000	-0.33372000	3.05603200
H	-8.01068100	0.72535800	2.53163200
H	-8.30788300	-2.24876800	-2.04673000
H	-9.67402300	-3.34358600	-1.85264800
H	-9.93178900	-1.59759500	-1.80743400
H	-10.11605600	1.65018700	2.03600000
H	-11.85116200	1.38395500	1.89487200
H	-10.73645100	0.01821800	1.78604600
H	-7.71002600	1.62336900	-2.99741000
H	-7.93710200	-0.09098300	-2.72109700
H	-8.77515500	0.70079800	-4.05923200
H	-14.93718300	3.60525500	-2.36153300
H	-14.53710200	2.23262500	-1.30038200
H	-13.79724000	3.84036900	-1.01390400
H	-13.40896000	4.53178900	-3.85413400
H	-11.78305300	3.83906000	-4.09415000
H	-12.15322800	4.72549000	-2.59107300
H	-9.94587200	-6.99360400	2.52449100
H	-10.72409700	-5.82381700	1.43078800
H	-9.03115500	-6.33726700	1.14403800
H	-8.22691700	-6.40377500	3.98227400
H	-7.67450100	-4.71683100	4.15745500
H	-7.26633300	-5.63071100	2.68155800
H	13.89656300	-0.92569500	-3.26424200
H	14.41624300	-2.33102000	-4.24612700
H	12.79823100	-1.61994000	-4.48639400
H	11.26488500	4.41418200	3.35177600
H	10.54229700	5.69691700	4.37238300
H	10.00757400	4.00389700	4.54820700
H	-13.89683800	0.92663100	-3.26334000
H	-14.41669900	2.33230700	-4.24462600
H	-12.79866700	1.62142400	-4.48532500
H	-11.26466300	-4.41544600	3.35012400
H	-10.54209100	-5.69857700	4.37024300
H	-10.00730200	-4.00563700	4.54666600
H	-7.07334300	4.27537800	-0.12156400
H	-7.85043900	3.46935400	-1.48173400
H	-8.48234400	3.24165700	0.14853600
H	8.48199700	-3.24195500	0.14782200
H	7.07294600	-4.27535800	-0.12325200
H	7.85050300	-3.46862400	-1.48274800

Compound 3N

DFT B3LYP/6-31G+(d,p), gas phase, S_0

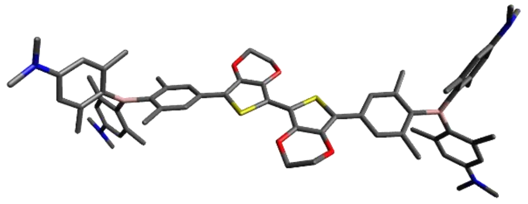

Point group: C_1

Total energy: -2513546.50 kcal mol^{-1}

Dipole moment: 0.05 D

Immaginary frequencies: 0

C	-4.63710100	-0.04056500	0.55481000
C	-5.49390400	0.33113100	1.60619900
C	-6.88260100	0.36420100	1.44878600
C	-7.48728100	-0.00889800	0.21672700
C	-6.62157000	-0.40155700	-0.84255900
C	-5.23672700	-0.39844600	-0.66593000
B	-9.06688700	0.01184600	0.02958000
C	-9.80871500	-1.26955800	-0.52300000
C	-9.88177700	1.31489300	0.39790800
C	-10.76461900	-1.17782200	-1.57497200
C	-11.39753600	-2.31532500	-2.08161000
C	-11.16397000	-3.60065200	-1.55007400
C	-10.21969200	-3.69405500	-0.50667600
C	-9.54741900	-2.57360600	-0.01376100
C	-11.05831400	1.25266100	1.19833600
C	-11.76071200	2.40859000	1.54709100
C	-11.37516100	3.68527200	1.08821000
C	-10.21180400	3.74952200	0.29351600
C	-9.47408400	2.60957400	-0.03302200
C	-8.55689900	-2.82226200	1.11003300
C	-11.12275200	0.14172800	-2.23703900
C	-11.59603100	-0.05446100	1.75513300
C	-8.24367900	2.82720600	-0.89592800
C	-7.14431800	-0.81856700	-2.20541700
C	-7.69957900	0.79424400	2.65437000
C	-3.18369100	-0.04550500	0.71442300
N	-11.84068700	-4.71979300	-2.02234500
N	-12.11274600	4.82391200	1.39309000
C	-13.16859900	4.74330600	2.39039400
C	-11.54083700	6.13509800	1.12690900
C	-12.63211200	-4.61354500	-3.23824100
C	-11.38162400	-6.04519300	-1.63531500
C	-2.41568100	-0.05223200	1.86342800
C	-1.01206100	-0.06191800	1.62363900
C	-0.65971200	-0.06587700	0.28685800
S	-2.12393100	-0.04527200	-0.69123200
C	4.63711400	-0.04055600	-0.55482900
C	5.49391300	0.33115500	-1.60621600
C	6.88261100	0.36422700	-1.44880700

C	7.48729400	-0.00888500	-0.21675400
C	6.62158700	-0.40155900	0.84252900
C	5.23674400	-0.39844900	0.66590500
B	9.06690500	0.01184200	-0.02961200
C	9.80870300	-1.26957100	0.52298100
C	9.88179900	1.31489000	-0.39791900
C	10.76456600	-1.17785900	1.57499300
C	11.39744600	-2.31537500	2.08164500
C	11.16388600	-3.60069300	1.55008600
C	10.21964600	-3.69407300	0.50665000
C	9.54740700	-2.57361200	0.01372000
C	11.05836700	1.25265600	-1.19830100
C	11.76077100	2.40858400	-1.54704800
C	11.37519000	3.68527000	-1.08820100
C	10.21181800	3.74952000	-0.29352800
C	9.47409400	2.60957200	0.03299900
C	8.55692500	-2.82224400	-1.11011400
C	11.12267200	0.14167800	2.23710000
C	11.59612500	-0.05447400	-1.75504100
C	8.24368600	2.82720900	0.89589900
C	7.14434000	-0.81858800	2.20537800
C	7.69957800	0.79430100	-2.65438800
C	3.18370500	-0.04550000	-0.71444000
N	11.84056500	-4.71984600	2.02237300
N	12.11277200	4.82390800	-1.39307300
C	13.16871400	4.74328600	-2.39028000
C	11.54087800	6.13509400	-1.12686800
C	12.63198700	-4.61360700	3.23827200
C	11.38149200	-6.04523800	1.63532600
C	2.41569500	-0.05222800	-1.86344600
C	1.01207500	-0.06191900	-1.62365700
C	0.65972600	-0.06587700	-0.28687600
S	2.12394400	-0.04526900	0.69121400
O	-2.92479000	-0.04980800	3.13916200
C	-1.96700800	-0.45609300	4.12713800
C	-0.64258800	0.25737600	3.91230700
O	-0.08906800	-0.09428000	2.63668300
O	2.92480600	-0.04980100	-3.13917900
C	1.96702500	-0.45608900	-4.12715500
C	0.64260200	0.25737400	-3.91232500
O	0.08908200	-0.09428200	-2.63670100
H	-5.06718700	0.60990400	2.56204600
H	-4.60915800	-0.70331100	-1.49942600
H	-12.08636300	-2.18718300	-2.90790300
H	-9.99588900	-4.65656800	-0.06257000
H	-12.62621500	2.30196500	2.18989700
H	-9.86490400	4.70405000	-0.08368200
H	-7.52427700	-2.67316400	0.78085500
H	-8.71629300	-2.15106000	1.95952400
H	-8.64654900	-3.84792800	1.48046400
H	-10.23785100	0.72187300	-2.51551100
H	-11.70310600	-0.03300800	-3.14821300
H	-11.71821100	0.77844300	-1.57583700
H	-10.81660400	-0.65575000	2.23284600
H	-12.36960700	0.13883900	2.50465400
H	-12.03545200	-0.67851200	0.97119000
H	-7.31998100	2.64797800	-0.33788700
H	-8.22214800	2.15984100	-1.76304400
H	-8.21521700	3.85442700	-1.27169300
H	-7.85471900	-0.09415900	-2.61692900
H	-6.31996400	-0.91202400	-2.91883900
H	-7.66574200	-1.77958300	-2.16308700
H	-7.06341800	0.85826900	3.54218100
H	-8.16559100	1.77235800	2.50059300
H	-8.50968800	0.09300400	2.87960000
H	-13.66739200	5.71159900	2.45942700
H	-13.92345400	4.00464400	2.10155300
H	-12.79248200	4.47501800	3.39128600
H	-12.26782100	6.90214700	1.39926000
H	-11.31849100	6.25719200	0.06161000
H	-10.61344100	6.31919200	1.69310800
H	-13.12644900	-5.56838300	-3.42547000
H	-13.41374100	-3.85435000	-3.13081400
H	-12.02573400	-4.35787100	-4.12262300
H	-12.04572300	-6.79313100	-2.07189400
H	-11.41632300	-6.17032100	-0.54796800
H	-10.35332000	-6.25570100	-1.97118700
H	5.06719200	0.60993900	-2.56205800
H	4.60917800	-0.70332100	1.49940200
H	12.08623900	-2.18725200	2.90797100
H	9.99584700	-4.65658000	0.06252900
H	12.62630300	2.30195700	-2.18981400
H	9.86491500	4.70405100	0.08366500
H	7.52429300	-2.67312500	-0.78097600
H	8.71636500	-2.15104400	-1.95959800
H	8.64656800	-3.84791100	-1.48054200
H	10.23775500	0.72178300	2.51560700
H	11.70304300	-0.03307300	3.14825900
H	11.71809900	0.77843700	1.57591300
H	10.81673400	-0.65575700	-2.23282300
H	12.36977000	0.13881300	-2.50449200
H	12.03546500	-0.67852800	-0.97105600
H	7.31998800	2.64802400	0.33784200
H	8.22212700	2.15981600	1.76299300
H	8.21524800	3.85441900	1.27169700
H	7.85486300	-0.09426900	2.61683200
H	6.32000700	-0.91190800	2.91884100
H	7.66562700	-1.77968100	2.16305600
H	7.06343500	0.85820900	-3.54222100
H	8.16546500	1.77248200	-2.50064500
H	8.50978000	0.09315400	-2.87956100
H	13.66749400	5.71158500	-2.45930800
H	13.92355800	4.00464500	-2.10134900
H	12.79268800	4.47495500	-3.39119400
H	12.26786200	6.90214200	-1.39922200
H	11.31854200	6.25718000	-0.06156500
H	10.61347800	6.31919900	-1.69305500
H	13.12629800	-5.56845700	3.42551300
H	13.41363700	-3.85443400	3.13084100
H	12.02561300	-4.35790800	4.12264800
H	12.04556000	-6.79318800	2.07192900
H	11.41622500	-6.17036600	0.54797900
H	10.35317200	-6.25572700	1.97116100
H	-2.40166800	-0.19391500	5.09457000
H	-1.82626300	-1.54406600	4.07529900
H	0.09556100	-0.04811400	4.65740100
H	-0.78223000	1.34550300	3.96311500
H	2.40168500	-0.19391100	-5.09458700
H	1.82628400	-1.54406300	-4.07531500
H	-0.09554600	-0.04811800	-4.65741900
H	0.78224100	1.34550200	-3.96313400

Compound 3

DFT B3LYP/6-31G+(d,p), gas phase, S_0

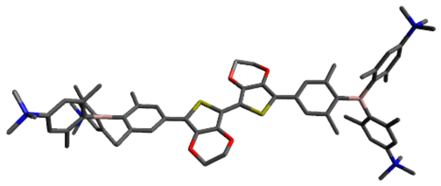

Point group: C_1

Total energy: -2613033.08 kcal mol^{-1}

Dipole moment: 0.27 D

Immaginary frequencies: 0

C	-4.63002100	-0.01484400	0.53283500
C	-5.49646400	0.24034700	1.61625500
C	-6.88224300	0.27305300	1.46823600
C	-7.49605900	-0.00070900	0.20302600
C	-6.61533200	-0.28316900	-0.89260300
C	-5.23435200	-0.26277300	-0.71741900
B	-9.04723100	0.00584300	0.02789600
C	-9.79152200	-1.12270700	-0.83957700
C	-9.95400400	1.14046000	0.71433500
C	-10.68193700	-0.80443800	-1.89514600
C	-11.32938800	-1.81801800	-2.62334600
C	-11.12147000	-3.15296500	-2.29983000
C	-10.25940000	-3.49113300	-1.25926700
C	-9.58394700	-2.49824200	-0.53746700
C	-11.05568500	0.82901100	1.54967900
C	-11.82977300	1.84783600	2.13245000
C	-11.53840100	3.18150600	1.87519700
C	-10.47032800	3.51311400	1.04472000
C	-9.66977200	2.51453200	0.47465300
C	-8.64131300	-2.96632900	0.55327200
C	-10.96196700	0.62389200	-2.31578900
C	-11.44121300	-0.59712300	1.88592700
C	-8.51006100	2.97468900	-0.38596100
C	-7.11107200	-0.56843000	-2.29930100
C	-7.67857500	0.56202800	2.72907300
C	-3.18394600	-0.01709200	0.69060300
N	-11.81174700	-4.26909200	-3.05726500
N	-12.35640100	4.30292700	2.48266400
C	-13.47997000	3.80332600	3.36146100
C	-11.46411100	5.18169600	3.34124800
C	-12.71512900	-3.76300100	-4.15808500
C	-10.77378200	-5.17181100	-3.69984500
C	-2.42071300	-0.01677900	1.85131400
C	-1.01884600	-0.03029000	1.61867400
C	-0.66206700	-0.04578700	0.27906800
S	-2.11595700	-0.03735900	-0.71193500
C	4.62990500	-0.01485100	-0.53287700
C	5.49638300	0.23979000	-1.61640500
C	6.88215200	0.27258500	-1.46836000
C	7.49591600	-0.00051500	-0.20298500
C	6.61517000	-0.28236600	0.89276600
C	5.23418600	-0.26207600	0.71753200
B	9.04709200	0.00595000	-0.02780200
C	9.79128000	-1.12239500	0.83997300
C	9.95398600	1.14027500	-0.71454100
C	10.68180700	-0.80383700	1.89535600
C	11.32932300	-1.81721300	2.62378200
C	11.12133100	-3.15225000	2.30067600
C	10.25910000	-3.49070400	1.26034100
C	9.58358000	-2.49801000	0.53832700
C	11.05584900	0.82835400	-1.54944800
C	11.83013500	1.84685500	-2.13255400
C	11.53878200	3.18065800	-1.87601400
C	10.47051500	3.51273400	-1.04595600
C	9.66976300	2.51448600	-0.47560900
C	8.64070200	-2.96642200	-0.55206400
C	10.96197000	0.62461700	2.31550100
C	11.44154600	-0.59796300	-1.88476900
C	8.50983500	2.97513500	0.38445100
C	7.11090600	-0.56674000	2.29964600
C	7.67855500	0.56093000	-2.72929400
C	3.18382500	-0.01719500	-0.69069000
N	11.81168600	-4.26817700	3.05833900
N	12.35698200	4.30174200	-2.48383600
C	13.48075400	3.80165900	-3.36209500
C	11.46494800	5.18011900	-3.34309000
C	12.71519400	-3.76179100	4.15891800
C	10.77379500	-5.17071900	3.70129200
C	2.42060500	-0.01743600	-1.85140600
C	1.01873100	-0.03092500	-1.61877200
C	0.66194300	-0.04586800	-0.27916400
S	2.11582200	-0.03694300	0.71184600
O	-2.93483600	-0.00678300	3.11480300
C	-1.97869900	-0.37932800	4.12971900
C	-0.65741100	0.33240900	3.90344200
O	-0.10218600	-0.04286400	2.62815500
O	2.93473000	-0.00792800	-3.11490200
C	1.97862000	-0.38094900	-4.12966500
C	0.65729200	0.33081100	-3.90369000
O	0.10208000	-0.04397500	-2.62825600
C	-12.97903700	5.14143800	1.38073800
C	-12.66793500	-5.08454000	-2.10475300
C	12.97936600	5.14077900	-1.38216700
C	12.66775200	-5.08388100	2.10594300
H	-5.07210900	0.42222900	2.59494700
H	-4.60617800	-0.46034200	-1.58120600
H	-11.98569400	-1.51970800	-3.42998400
H	-10.08248300	-4.52627600	-0.98735200
H	-12.64735200	1.55447500	2.77732500
H	-10.22519100	4.54716200	0.82691700
H	-7.59675300	-2.83017000	0.25721900
H	-8.77492300	-2.41447600	1.48685600
H	-8.79354800	-4.02642900	0.77204800
H	-10.03961800	1.18294700	-2.49880300
H	-11.54750100	0.65650200	-3.23784100
H	-11.52148600	1.16408300	-1.54647200
H	-10.58681100	-1.17620700	2.24789800
H	-12.20480200	-0.62579800	2.66706600
H	-11.83989700	-1.11916800	1.01108400
H	-7.55508900	2.82811400	0.12774500
H	-8.44520500	2.42500300	-1.32808800
H	-8.60183200	4.03646200	-0.62907900
H	-7.85945400	0.15429700	-2.63739600
H	-6.28139700	-0.52892100	-3.00937300
H	-7.56207800	-1.56264500	-2.37980100
H	-7.02939900	0.50664000	3.60610000
H	-8.12057200	1.56346200	2.71314600
H	-8.49494200	-0.14899200	2.88609200
H	-14.00903000	4.67266100	3.75087600
H	-14.16166400	3.19544400	2.76850600
H	-13.06596000	3.22663400	4.18719900
H	-12.07438600	5.97221400	3.77969700
H	-10.68199900	5.61623200	2.72288900
H	-11.02182600	4.56310000	4.12166600
H	-13.16466000	-4.62876300	-4.64355100
H	-13.49624900	-3.13800700	-3.72792000
H	-12.12593400	-3.20220200	-4.88209400
H	-11.29138000	-5.95839700	-4.25028800
H	-10.14761100	-5.61047100	-2.92630300

H	-10.16594900	-4.56967700	-4.37460600
H	5.07205800	0.42120700	-2.59519600
H	4.60599300	-0.45913000	1.58142200
H	11.98569400	-1.51868000	3.43028600
H	10.08210200	-4.52592600	0.98877600
H	12.64785300	1.55313500	-2.77708900
H	10.22538800	4.54690300	-0.82871900
H	7.59621200	-2.83036000	-0.25570800
H	8.77391100	-2.41470900	-1.48578600
H	8.79302300	-4.02654000	-0.77068600
H	10.03969800	1.18428500	2.49692400
H	11.54631100	0.65749200	3.23830300
H	11.52282200	1.16403200	1.54658800
H	10.58690700	-1.17798400	-2.24461100
H	12.20385200	-0.62707400	-2.66714800
H	11.84203400	-1.11876800	-1.00998800
H	7.55501800	2.82851300	-0.12953500
H	8.44455800	2.42581900	1.32676300
H	8.60167900	4.03698900	0.62719100
H	7.85905700	0.15640600	2.63738200
H	6.28118600	-0.52707700	3.00965600
H	7.56221000	-1.56076900	2.38073100
H	7.02938900	0.50531400	-3.60631500
H	8.12072700	1.56229000	-2.71374200
H	8.49481200	-0.15029000	-2.88601600
H	14.00996800	4.67078000	-3.75178300
H	14.16226100	3.19402400	-2.76867100
H	13.06693300	3.22459600	-4.18766800
H	12.07536800	5.97039300	-3.78177600

H	10.68269600	5.61499400	-2.72515000
H	11.02284200	4.56115000	-4.12331400
H	13.16480100	-4.62742200	4.64454800
H	13.49625500	-3.13689600	3.72850100
H	12.12607900	-3.20081600	4.88285400
H	11.29146200	-5.95715200	4.25188800
H	10.14753500	-5.60960100	2.92794900
H	10.16604400	-4.56839900	4.37595900
H	-2.42459300	-0.08711800	5.08212600
H	-1.84194400	-1.46722000	4.10927400
H	0.07987500	0.03797500	4.65240000
H	-0.79168300	1.42074800	3.93123600
H	2.42450200	-0.08911200	-5.08219300
H	1.84192000	-1.46884000	-4.10876800
H	-0.07997200	0.03603500	-4.65253600
H	0.79151000	1.41914500	-3.93192500
H	-13.61213900	4.49516900	0.77342100
H	-13.57143100	5.93363000	1.84014800
H	-12.19239100	5.57482700	0.76736300
H	-13.40305400	-4.42105400	-1.65011600
H	-13.16432700	-5.87257600	-2.67234100
H	-12.03633400	-5.52305400	-1.33554800
H	13.61226600	4.49478400	-0.77434800
H	13.57192900	5.93270400	-1.84182000
H	12.19257500	5.57452300	-0.76922800
H	13.40282000	-4.42052300	1.65103800
H	13.16421200	-5.87177000	2.67367700
H	12.03605100	-5.52259000	1.33692900

References

[1] C.-W. Chiu, F. P. Gabbaï, *Organometallics* **2008**, *27*, 1657-1659.

[2] G. A. Sotzing, J. R. Reynolds, P. J. Steel, *Adv. Mater.* **1997**, *9*, 795-798.

[3] S. Griesbeck, M. Ferger, C. Czernetzi, C. Wang, R. Bertermann, A. Friedrich, M. Haehnel, D. Sieh, M. Taki, S. Yamaguchi, T. B. Marder, *Chem. Eur. J.* **2019**, *25*, 7679-7688.

[4] S. Griesbeck, Z. Zhang, M. Gutmann, T. Lühmann, R. M. Edkins, G. Clermont, A. N. Lazar, M. Haehnel, K. Edkins, A. Eichhorn, M. Blanchard-Desce, L. Meinel, T. B. Marder, *Chem. Eur. J.* **2016**, *22*, 14701-14706.

[5] S. S. Zalesskiy, V. P. Ananikov, *Organometallics* **2012**, *31*, 2302-2309.

[6] R. Uson, L. A. Oro, J. A. Cabeza, H. E. B. Bryndza, M. P. Stepro, *Inorg. Synth.* **1985**, *23*, 126-130.

[7] S.-F. Liu, Q. Wu, H. L. Schmider, H. Aziz, N.-X. Hu, Z. Popović, S. Wang, *J. Am. Chem. Soc.* **2000**, *122*, 3671-3678.

[8] G. Sheldrick, *Acta Crystallogr.* **2015**, *A71*, 3-8.

[9] G. Sheldrick, *Acta Crystallogr.* **2008**, *A64*, 112-122.

[10] C. B. Hübschle, G. M. Sheldrick, B. Dittrich, *J. Appl. Crystallogr.* **2011**, *44*, 1281-1284.

[11] K. Brandenburg, Diamond (version 4.4.0) - Crystal and Molecular Structure Visualization, Crystal Impact H. Putz & K. Brandenburg GbR, Bonn (Germany), **2017**.

[12] J. B. Chaires, N. Dattagupta, D. M. Crothers, *Biochem.* **1982**, *21*, 3933-3940.

[13] M. Hranjec, B. Lučić, I. Ratkaj, S. K. Pavelić, I. Piantanida, K. Pavelić, G. Karminski-Zamola, *Eur. J. Med. Chem.* **2011**, *46*, 2748-2758.

[14] J. D. McGhee, P. H. von Hippel, *J. Mol. Biol.* **1974**, *86*, 469-489.

[15] J.-L. Mergny, L. Lacroix, *Oligonucleotides* **2003**, *13*, 515-537.

[16] M. J. Frisch, G. W. Trucks, H. B. Schlegel, G. E. Scuseria, M. A. Robb, J. R. Cheeseman, G. Scalmani, V. Barone, B. Mennucci, G. A. Petersson, H. Nakatsuji, M. Caricato, X. Li, H. P. Hratchian, A. F. Izmaylov, J. Bloino, G. Zheng, J. L. Sonnenberg, M. Hada, M. Ehara, K. Toyota, R. Fukuda, J. Hasegawa, M. Ishida, T. Nakajima, Y. Honda, O. Kitao, H. Nakai, T. Vreven, J. A. Montgomery, J. E. Peralta, F. Ogliaro, M. Bearpark, J. J. Heyd, E. Brothers, K. N. Kudin, V. N. Staroverov, R. Kobayashi, J. Normand, K. Raghavachari, A. Rendell, J. C. Burant, S. S. Iyengar, J. Tomasi, M. Cossi, N. Rega, J. M. Millam, M. Klene, J. E. Knox, J. B. Cross, V. Bakken, C. Adamo, J. Jaramillo, R. Gomperts, R. E. Stratmann, O. Yazyev, A. J. Austin, R. Cammi, C. Pomelli, J. W. Ochterski, R. L. Martin, K. Morokuma, V. G. Zakrzewski, G. A. Voth, P. Salvador, J. J. Dannenberg, S. Dapprich, A. D. Daniels, Farkas, J. B. Foresman, J. V. Ortiz, J. Cioslowski, D. J. Fox, Revision A.03 ed., Gaussian Inc., Wallingford CT, **2016**.

[17] M. D. Hanwell, D. E. Curtis, D. C. Lonie, T. Vandermeersch, E. Zurek, G. R. Hutchison, *J. Cheminform.* **2012**, *4*, 17.

[18] T. Lu, F. Chen, *J. Comput. Chem.* **2012**, *33*, 580-592.

[19] C. Lee, W. Yang, R. G. Parr, *Phys. Rev. B* **1988**, *37*, 785-789.

[20] G. A. Petersson, A. Bennett, T. G. Tensfeldt, M. A. Al-Laham, W. A. Shirley, J. Mantzaris, *J. Chem. Phys.* **1988**, *89*, 2193-2218.

[21] G. A. Petersson, M. A. Al-Laham, *J. Chem. Phys.* **1991**, *94*, 6081-6090.

[22] S. Bolte, F. P. Cordelières, *J. Microsc.* **2006**, *224*, 213-232.

[23] J. Cao, J. W. Kampf, M. D. Curtis, *Chem. Mater.* **2003**, *15*, 404-411.

[24] W. Saenger, *Principles of nucleic acid structure*, Springer Science & Business Media, New York, **2013**.
